# Phylogenetic evidence that migratory behavior increases ungulate extinction rates

**DOI:** 10.1371/journal.pbio.3004072

**Published:** 2026-11-05

**Authors:** Joel O. Abraham, Kaedan O’Brien, Nathan S. Upham, Brett R. Jesmer, Faysal Bibi, J. Tyler Faith

**Affiliations:** 1 Department of Ecology and Evolutionary Biology, Princeton University, Princeton, New Jersey, United States of America; 2 Department of Anthropology, SUNY Oneonta, Oneonta, New York, United States of America; 3 School of Life Sciences, Arizona State University, Tempe, Arizona, United States of America; 4 Department of Fish and Wildlife Conservation, Virginia Tech, Blacksburg, Virginia, United States of America; 5 School of Biological Sciences, Illinois State University, Normal, Illinois, United States of America,; 6 Museum für Naturkunde, Leibniz Institute for Evolution and Biodiversity Science, Berlin, Germany; 7 Department of Anthropology, University of Utah, Salt Lake City, Utah, United States of America; 8 Natural History Museum of Utah, University of Utah, Salt Lake City, Utah, United States of America; Universidade de São Paulo, BRAZIL

## Abstract

Widespread instances of migratory collapse have been documented within the past several decades, indicating that migration may be a fragile strategy in the Anthropocene. Yet the fragility of migration as a strategy for navigating seasonally variable resource availability may extend deeper into evolutionary time. We used phylogenetic imputation to infer the migratory habits of 51 ungulate species that went extinct during the late Quaternary and evaluated whether migration enhances extinction rates on macroevolutionary timescales. We found that ~80% (41 of 51) of recently extinct species were migratory, whereas only 46% (95 of 207) of extant ungulates are migratory. Furthermore, Hidden State Speciation and Extinction modeling indicated that migratory species likely experienced higher speciation and extinction rates compared to nonmigratory species, resulting in elevated lineage turnover for migratory ungulates, though diversification parameter estimates depended strongly upon whether extinct species were included in analyses. The extinctions of these ungulates led to negligible declines in global ungulate functional richness (0.8%) but more substantial declines (2.7%) across Africa. Altogether, our findings indicate that migration has complex macroevolutionary consequences, enhancing both speciation and extinction rates, which may render migration an evolutionarily fragile strategy, particularly as increasingly widespread and intensive anthropogenic activity cause gradients of resource availability to become severed (*e.g*., via human infrastructure) and more unpredictable (*e.g*., via climate change).

## Abstract 2

Collapsing ungulate migrations over the last several decades suggest that migration may be uniquely risky in the Anthropocene, or may represent the acceleration of a long-term evolutionary trend wherein migration increases extinction rates. We find that extinct species were disproportionately migratory, with migratory lineages exhibiting faster extinction, speciation, and diversification rates-evidence that migration may be evolutionarily fragile, associated with heightened extinction risk.

## Introduction

Animal migrations are imperiled across the globe [1–3]. Many migrations have collapsed in recent decades, with detrimental consequences for migratory populations [1–3] and the ecosystems they inhabit [4–6]. Some of the best-known and most charismatic animal migrations are those of ungulates (*i.e.,* hooved mammals) [3,7], and it is widely recognized that the collapse of ungulate migrations is due in large part to intensifying human impacts [2,3], such as the construction of physical barriers (*i.e.,* roads and fences [8,9]), hunting and overharvesting [10–12], changes in land use [9,13], and anthropogenic climate change [14,15]. The collapses of these ungulate migrations have coincided with the declines and/or extirpations of populations and, in some cases, near-extinctions of entire species [8–14].

However, many of the factors that have contributed to the collapse of contemporary ungulate migrations have also played out over millions of years, such that elevated extinction rates may be a more general feature of migration: both climatic and geological changes may have impacted ancient migratory systems, altering spatiotemporal distributions of resources or presenting physical barriers to migration. Indeed, there is reason to believe that migration is a high-risk behavioral strategy in general, and that the declines in migratory ungulate species observed today may be part of a longer-term trend extending well before the Anthropocene [16], albeit one exacerbated by modern human impacts [8–14]. For example, numerous likely migratory ungulates have gone extinct over the past several thousand years [17]. Furthermore, the late Quaternary megafaunal extinctions saw the catastrophic loss of Earth’s large-bodied mammal species [16,18,19], with a growing body of evidence suggesting that many of these species were migratory [17,19–24]. Together, these lines of evidence suggest that contemporary declines of migratory species may reflect the acceleration of a longer-term trend, one that potentially extends back to the Pleistocene or perhaps even deeper in time.

As a behavioral adaptation for navigating seasonal bottlenecks in resource availability, migration offers many benefits but also poses risks. By tracking spatiotemporal variation in vegetation growth [15,25], migratory individuals are able to circumvent seasonal forage bottlenecks [26,27], thereby accruing more resources and increasing their fitness [28,29]. These benefits enable migratory species to sustain large populations (some of the most abundant ungulate populations to this day are migratory [6,26,27]), which might *reduce* extinction risk from the stochastic processes that dominate at small population sizes [30,31]. Although these benefits may explain why migration has evolved multiple times and proliferated across the ungulate phylogeny [17], migrating also involves intrinsic risks that may *elevate* extinction rates. Ungulates often experience substantial mortality during migrations, both from the direct physiological costs, which can be severe [32–34], and from indirect pressures such as increased thermal exposure, predation risk, and disease burdens [14,35–37]. Moreover, deriving benefits from migration requires that individuals accurately track spatiotemporal variation in forage availability [15,28]. When this coupling breaks down—either because individuals are prevented from tracking the availability of high-quality forage [3,8] or because the predictability of spatiotemporal gradients in forage availability diminishes [15,19,38]—populations can collapse and subsequent extirpation and extinction can follow [2,3,14].

Rapid population declines following migratory collapse may not only elevate extinction risk but also promote speciation within migratory lineages. Small, isolated populations that arise from such collapses are more likely to diverge genetically through drift, inbreeding, and further bottlenecks, potentially facilitating rapid speciation [39–41]. Migratory behavior itself may also foster divergence when populations vary in their migratory routes or phenology, creating opportunities for genetic isolation [42–48]. Indeed, such speciation pathways have been documented within migratory birds [42,45], but comparable examples from ungulates are lacking. If migration accelerates both speciation and extinction, migratory ungulates should experience faster lineage turnover, with the frequent emergence of new migratory species that persist only briefly before going extinct (termed ‘ephemeral speciation’ [49]). Together, these dynamics suggest that migration may be an evolutionarily fragile strategy—one that has long elevated extinction rates but is particularly precarious within the Anthropocene, as human activities rapidly and profoundly disrupt migratory systems.

Herein, we evaluate whether migration is a fragile strategy in general, exposing ungulates to enhanced rates of extinction even on macroevolutionary (≥10^4^ years) timescales. Drawing from fossil and present-day datasets, we use data on characters that are known to be strongly associated with migratory capacity (body size, grass dependence, and latitude [17]) to infer the migratory capabilities of 51 ungulates that have gone extinct through the late Quaternary (late Pleistocene and Holocene). We then compare the prevalence of migratory behavior between extinct and extant ungulate species. Furthermore, to test whether macroevolutionary rates have differed among migratory and nonmigratory lineages, we employ Hidden State Speciation and Extinction (HiSSE) modeling to compare evolutionary parameter reconstructions associated with these behaviors, both including and excluding extinct ungulates to assess the effects of their inclusion on parameter estimates. Finally, since migratory ungulates are functionally important within ecosystems because of their unique traits, we explore the consequences of losing extinct ungulate species for ungulate functional diversity. Throughout, we define migration inclusively as the capacity to migrate: we consider a species migratory if any of its populations, at any point in time, exhibit seasonal round-trip movements between discrete areas. This definition captures the evolutionary potential for migration, recognizing that its expression today varies across space and time and is confounded by anthropogenic influences [2,3,17,50,51].

## Results

### Migratory behavior in extinct ungulates

To evaluate the hypothesis that exhibiting migratory behavior makes ungulate species more susceptible to extinction in general, we first reconstructed the migratory habits of extinct ungulates. Extant migratory ungulates tend to be significantly larger, more grass-dependent, and live at higher latitudes (where they are exposed to greater spatiotemporal variation in resources) as compared to their nonmigratory counterparts [17], so we collated data on these three characters (body mass, dietary grass fraction, and latitudinal range centroid) for extinct ungulates (S1 Data). We then used phylogenetic imputation with 100 different tree conformations (to account for phylogenetic uncertainty; S2 Data) to reconstruct the migratory habits of 51 extinct ungulate species for which all variables were available (Fig 1a). We fit different models of trait evolution and found that lambda (λ) models of trait evolution best fit the data (*i.e.,* lower AIC_c_) for all 100 tree topologies (Table A in S1 Appendix). Estimates of λ ranged between 0.880 and 0.912 across trees (Table A in S1 Appendix), indicating slightly weaker phylogenetic signal than expected under Brownian motion. We then used these λ estimates for imputation.

**Fig 1:**
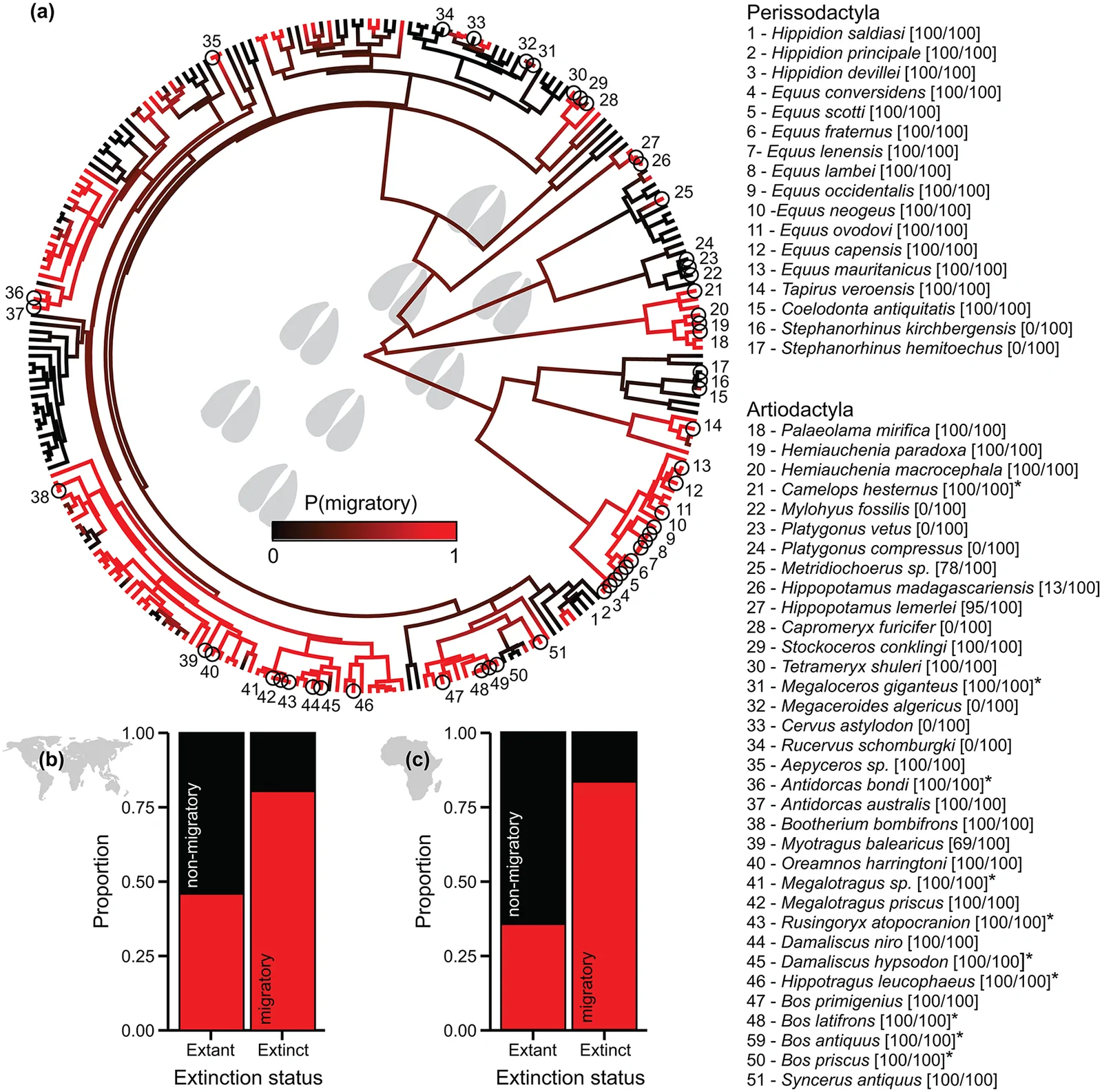
Reconstructions of migratory behavior in extinct ungulates. **(a)** Phylogenetic imputation-based reconstructions of migratory habits for 51 recently extinct ungulates from data on body mass, dietary grass fraction, and latitudinal range centroid across a set of 100 ungulate phylogenies. Shown in (a) is a representative phylogeny with branches colored by ancestral state reconstructions arising from phylogenetic imputation; branches *redder* in color correspond to a higher probability of migration. Tips corresponding to extinct taxa are circled and uniquely numbered, and the species names associated with each number are listed to the right of the phylogeny. Numbers in brackets after species names reflect the number of tree configurations for which a particular species was reconstructed as migratory, and asterisks denote species for which reliable fossil evidence corroborates our reconstructions that the species exhibited migratory behavior. Comparing the proportion of migratory species (*red bars*) between extant and extinct species, we found that extinct species were disproportionately migratory, considering **(b)** all extinct species and **(c)** just African species. Proportions in (b) and (c) reflect the median values across all 100 tree topologies. Map graphics were generated using the R package ‘maps’ v. 3.4.3 [94]. The data underlying this figure can be found in S1 Data, S2 Data, and at https://doi.org/10.5061/dryad.jm63xsjsp.

Approximately 80% (median = 41 out of 51, range = [39,42]) of extinct taxa were reconstructed as capable of migration (Fig 1b), whereas less than half (95 out of 207, 46%) of extant ungulate species exhibit evidence of migratory behavior. Across all 100 tree configurations, the incidence of migratory behavior was significantly higher amongst extinct ungulates relative to extant taxa (phyloGLM; Fig A in S1 Appendix), indicating that disproportionate representation of migratory behavior amongst extinct species is robust to phylogenetic uncertainty [52].

We conducted a literature review to validate the behavioral reconstructions resulting from our imputation approach (Table B in S1 Appendix). Because migratory behavior can vary widely among populations within a species [51], and most fossil inferences are based on remains from only one or two sites, absence of fossil evidence for migration from a given locality does not imply that the species as a whole was nonmigratory. Positive evidence, by contrast, is more definitive: if migratory behavior is identified within a fossil population, we can confidently infer that the species had the capacity to migrate. Of the 16 extinct species for which fossil-based evidence for or against migratory behavior exists, fossil evidence corroborated our imputation-based reconstructions of migratory behavior for 10 species (63%). For the remaining six species, our imputation approach indicated migratory behavior whereas fossil evidence did not. These apparent mismatches may simply reflect population-level variation or limited sampling for those species, rather than a failure of our imputation method. Notably, no species imputed as nonmigratory showed conclusive fossil evidence of migration.

Extinct African ungulates are well-represented in our dataset because relevant character data, particularly estimates of dietary grass fraction [53], are more readily available (see Materials and methods): altogether, our dataset includes the majority of African taxa known to have become extinct during the late Pleistocene and Holocene (18 of 24 ungulates [54,55]), with particularly good coverage for sub-Saharan Africa (11 of 13 ungulates). Given our greater confidence that the extinct African ungulate data are representative of continental-scale extinctions over the past ~100,000 years, we repeated our comparison on these data. The effect was even more pronounced within African ungulates (Fig 1c), where extinct taxa again exhibited a significantly higher likelihood of being migratory than extant taxa across all 100 tree topologies (Fig A in S1 Appendix). More specifically, 13–16 of the 18 extinct African species (72%–89%) in our dataset were likely to have been migratory, compared to 33 out of 93 extant species (36%).

The ungulates in our global dataset went extinct between 63 thousand years (ka) ago and the 20th century (the most recently extinct ungulate, *Rucervus schomburgki*, persisted until the mid- to late 1900s [56,57]). Most species in our dataset (29 of 51, 57%) disappeared from the fossil record between ~14 ka and ~10 ka (see S1 Data), reflecting the interval of greatest losses in megafaunal biodiversity, the late Quaternary megafaunal extinctions [58]. Of the 29 species that went extinct during this interval, 24 (83%) were reconstructed as capable of migration. Consistent with global patterns, Africa also saw numerous extinctions near the end of the Pleistocene, though they were more drawn out, spanning from ~14 ka through ~6 ka. Of the nine African taxa that went extinct during this interval, all but one (89%) were reconstructed as migratory.

### Evolutionary rates within ungulates

Migratory behavior might modify ungulate macroevolutionary dynamics, resulting in different extinction as well as speciation rates between migratory and nonmigratory lineages. To estimate evolutionary rates across the ungulate clade (the crown age of which is *ca.* 60 million years [Ma] old [52]), we used a HiSSE modeling approach (Fig 2). By including hidden states (which exhibit distinct diversification dynamics and transition rates from the observed states), HiSSE models specifically account for the presence of unmeasured or co-distributed factors that might also impact evolutionary rates [59,60], thus reducing the potentiality that any rate differences are the result of co-distributed factors (*e.g.*, grass dependence, body size, and latitude in this case, which are known to impact mammal macroevolutionary dynamics [52,61,62]). Because the effects of incorporating fossil taxa into state-dependent diversification analyses remain uncertain and have been demonstrated to potentially reduce [63,64] or exacerbate [65] false positives (*e.g.,* inferring that a trait impacts macroevolutionary dynamics when it does not), we fitted our HiSSE models including extinct taxa as well as only considering extant species, to assess the sensitivity of inferred diversification dynamics to the inclusion of fossils. For the same reason, we focus our interpretation on the direction and consistency of migration-associated differences across phylogenetic tree topologies rather than on the precise magnitude of parameter estimates, which can be sensitive to model specification and parameterization.

**Fig 2:**
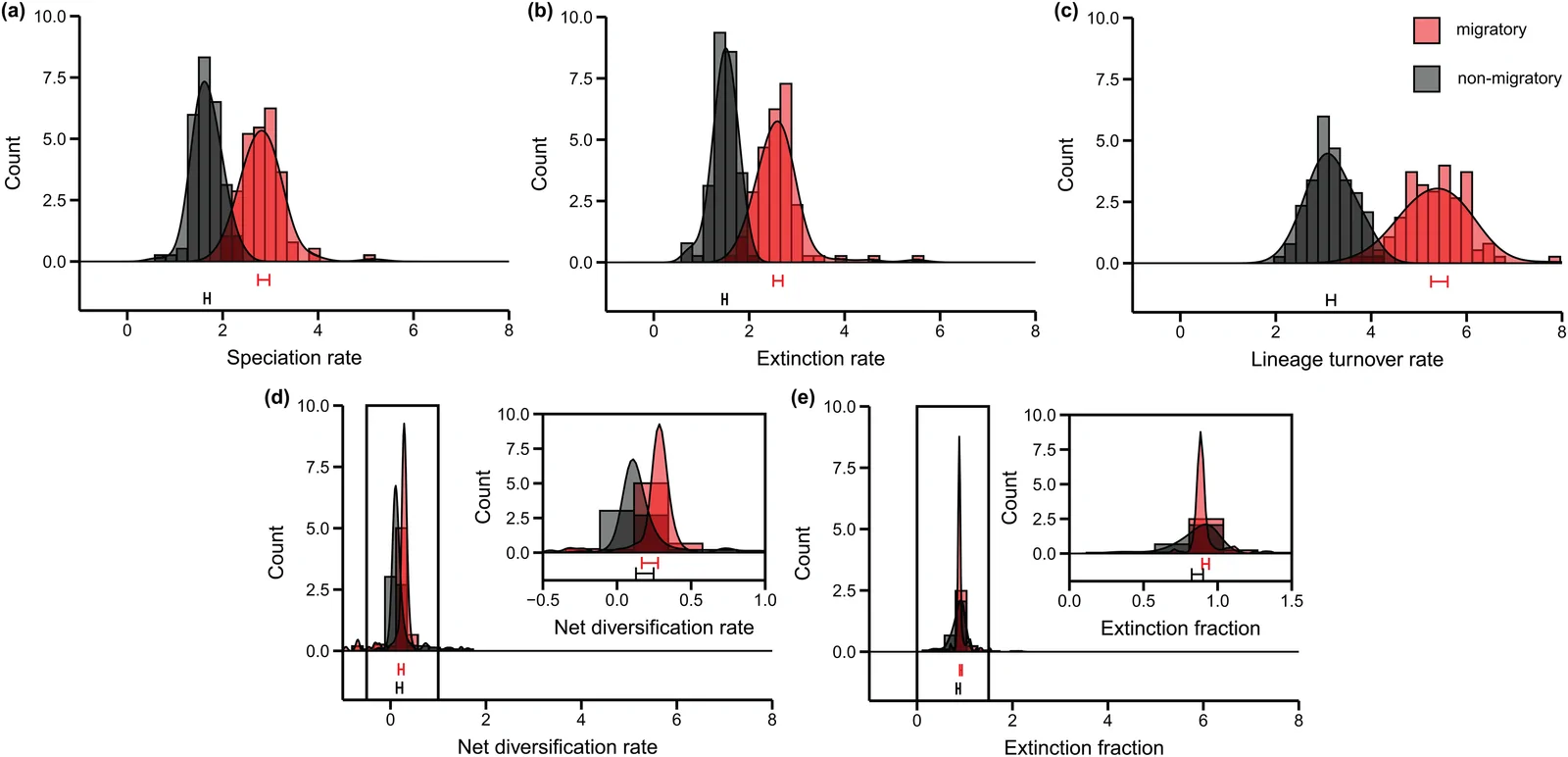
Fossil-inclusive Hidden State Speciation and Extinction (HiSSE) model estimates of evolutionary parameters for migratory and nonmigratory species across all 100 tree topologies. In nearly all topologies, migratory species exhibited significantly higher **(a)** extinction rates and **(b)** speciation rates than did nonmigratory species. Consequently, **(c)** lineage turnover rates were also elevated for migratory species, as were **(d)** net diversification rates to a lesser degree. However, **(e)** migration did not have a consistent effect on extinction fraction across tree topologies. *Red bars and curves* correspond to distributions of parameter means (within-tree averages) for migratory species for all 100 trees and *black bars and curves* correspond to distributions of parameter means for nonmigratory species. *Red and black lines* below the x-axes reflect 95% confidence intervals on mean parameter estimates across all 100 trees for migratory and nonmigratory lineages, respectively. Rates (a–d) are measured in units of species lineage^−1^ Ma^−1^, while extinction fraction (e) is unitless. Insets in (d) and (e) correspond to the boxed regions in the main panels. The data underlying this figure can be found at https://doi.org/10.5061/dryad.jm63xsjsp.

When extinct taxa were included, HiSSE models were preferred (*i.e.,* lower AIC_c_) to BiSSE (Binary State Speciation and Extinction; no hidden states) and CID-2 (character-independent diversification; only hidden states) models for 93 of 100 tree conformations (Table C in S1 Appendix), indicating that models featuring migration-dependent diversification generally explained parameter heterogeneity better than models lacking trait dependence, though the inclusion of hidden states suggested that additional factors contributed to rate heterogeneity as well; for the remaining seven trees, BiSSE models were preferred in two cases and CID-2 models were preferred in five cases (Table C in S1 Appendix). Furthermore, across all five macroevolutionary parameter classes, phyloGLMs with migration as a predictor generally outperformed phyloLMs with body size, grass dependence, or latitude as predictors, as assessed by AIC_c_ (Table D in S1 Appendix): in the eight cases in which migration phyloGLMs did not yield the lowest AIC_c_, grass dependence better predicted parameter variation (in two trees for speciation rate, in one tree for diversification rate, and in five trees for extinction fraction). Together, these findings suggest that, while other factors contribute to macroevolutionary parameter heterogeneity across lineages, migration is a stronger predictor of this heterogeneity than these correlated traits and plays a foremost role in structuring ungulate macroevolutionary dynamics.

Migratory species exhibited consistently elevated speciation and extinction rates relative to nonmigratory species across trees (phyloGLM; Fig 2a, 2b). Speciation rates were higher for migratory taxa in all tree configurations and differences were significant in 93 of 100 trees. Across trees, mean speciation rates averaged 2.825 species lineage^−1^ Ma^−1^ (95% confidence interval = [2.740, 2.981]) for migratory species and 1.673 species lineage^−1^ Ma^−1^ (95% CI = [1.617, 1.730]) for nonmigratory species, such that migratory species exhibited higher speciation rates by 1.152 species lineage^−1^ Ma^−1^ (95% CI = [1.052, 1.252]) on average (Fig 2a). Extinction rates were likewise significantly elevated within migratory lineages in all 100 tree topologies. Migratory lineages exhibited higher extinction rates by 1.117 species lineage^−1^ Ma^−1^ (95% CI = [1.008, 1.227]) across trees, averaging 2.603 species lineage^−1^ Ma^−1^ (95% CI = [2.505, 2.701]) for migratory lineages and 1.486 species lineage^−1^ Ma^−1^ (95% CI = [1.437, 1.535]) for nonmigratory lineages (Fig 2b).

Consequently, migratory lineages also exhibited consistently higher lineage turnover and generally higher net diversification rates relative to nonmigratory lineages (phyloGLM; Fig 2c, 2d). Lineage turnover rates, which were significantly elevated for migratory lineages in all 100 tree topologies, averaged 5.428 species lineage^−1^ Ma^−1^ (95% CI = [5.253, 5.604]) for migratory lineages and 3.159 species lineage^−1^ Ma^−1^ (95% CI = [3.073, 3.246]) for nonmigratory lineages, such that turnover rates were greater by 2.269 species lineage^−1^ Ma^−1^ (95% CI = [2.093, 2.446]) for migratory lineages on average across trees. Net diversification rates, while generally elevated in migratory lineages, were significantly elevated in just over half of the tree configurations tested (51 trees), and were nonsignificantly higher (in 32 trees) or significantly lower (in 17 trees) within migratory lineages in the remaining trees; net diversification rates averaged 0.222 species lineage^−1^ Ma^−1^ (95% CI = [0.167, 0.277]) for migratory lineages and 0.188 species lineage^−1^ Ma^−1^ (95% CI = [0.128, 0.247]) for nonmigratory lineages, with an average difference of 0.035 species lineage^−1^ Ma^−1^ (95% CI = [−0.079, 0.149]) across tree conformations.

In contrast to other diversification parameters, estimates of extinction fraction (*i.e.,* the rate of extinction standardized by the rate of speciation) exhibited pronounced variability across tree topologies (Fig 2e): though extinction fractions differed significantly between migratory and nonmigratory lineages for the majority of trees (66/100), the direction of this difference varied, with migratory lineages exhibiting significantly higher extinction fractions in 23 of 100 trees and significantly lower extinction fractions in 43 of 100 trees (Fig B in S1 Appendix). The average extinction fraction across trees was 0.919 [95% CI: 0.896, 0.942] for migratory lineages and 0.864 [95% CI: 0.825, 0.902] for nonmigratory lineages, such that the average difference was 0.056 [95% CI: −0.001, 0.113] (Fig 2e). Across fossil-inclusive diversification parameter estimates, confidence intervals were generally wider for migratory than for nonmigratory lineages (Fig 2), indicating greater variability in parameter reconstructions across tree configurations.

To assess whether these patterns depended on the inclusion of fossil taxa, we repeated all analyses with extant species only (Figs C, D in S1 Appendix). In these analyses, support for migration-dependent diversification dynamics was less consistent across phylogenetic tree conformations. Firstly, HiSSE models outperformed BiSSE and CID-2 models in 88 of 100 trees, while CID-2 models were preferred in the remaining 12 trees (Table E in S1 Appendix). Also, for the resultant rate estimates, migration best predicted variation in extinction rates for 68 of 100 trees, speciation rates for 52 of 100 trees, lineage turnover rates for 50 of 100 trees, net diversification rates for 96 of 100 trees, and extinction fraction for 66 of 100 trees relative to other traits (Table F in S1 Appendix). In the cases where migration was not the best predictor of rate variation, body mass was generally the top-performing predictor; in only two trees was grass dependence the strongest predictor, either for (1) both extinction and turnover rates or (2) for just turnover rates (Table F in S1 Appendix).

In extant-only analyses, migratory species continued to exhibit elevated speciation and diversification rates (Fig C in S1 Appendix), but patterns involving extinction rates, turnover rates, and extinction fraction differed from the fossil-inclusive analyses and varied considerably across tree topologies. Also, in contrast to fossil-inclusive analyses, confidence intervals for migratory lineages were narrower than those of nonmigratory lineages (Fig C in S1 Appendix).

In extant-only analyses, migratory lineages exhibited higher speciation rates in all trees and differences were significant in 94 of 100 trees: the average difference was 0.082 species lineage^−1^ Ma^−1^ (95% CI = [0.077, 0.087]) across trees, with speciation rates averaging 0.292 species lineage^−1^ Ma^−1^ (95% CI = [0.287, 0.298]) for migratory lineages and 0.210 species lineage^−1^ Ma^−1^ (95% CI = [0.204, 0.216]) for nonmigratory lineages (Fig C in S1 Appendix). Net diversification rates were likewise consistently elevated among migratory taxa in all 100 trees and significantly so in 59 of 100 trees: migratory lineages exhibited higher diversification rates by 0.187 species lineage^−1^ Ma^−1^ (95% CI = [0.175, 0.200]) on average, with diversification rates of 0.280 species lineage^−1^ Ma^−1^ (95% CI = [0.274, 0.285]) for migratory and 0.092 species lineage^−1^ Ma^−1^ (95% CI = [0.082, 0.103]) for nonmigratory lineages (Fig Cd in S1 Appendix).

In contrast, other parameters showed weaker and partially opposing patterns relative to fossil-inclusive analyses. Extinction rates and extinction fractions were generally lower among migratory lineages, significantly so in 70 of 100 and 78 of 100 trees, respectively, while lineage turnover rate differences varied in direction across trees, significantly higher for migratory lineages in 41 and significantly lower in 50 of 100 trees (Fig D in S1 Appendix). Extinction rates were 0.013 species lineage^−1^ Ma^−1^ (95% CI = [0.010, 0.015]) and 0.118 species lineage^−1^ Ma^−1^ (95% CI = [0.104, 0.131]) for migratory and nonmigratory lineages respectively, with an average difference of −0.105 species lineage^−1^ Ma^−1^ (95% CI = [−0.121, −0.090]) across trees (Fig Cb in S1 Appendix); extinction fractions were 0.082 (95% CI = [0.062, 0.101]) and 0.491 (95% CI = [0.452, 0.530]) for migratory and nonmigratory lineages, with an average difference of −0.409 (95% CI = [−0.461, −0.358]) (Fig Ce in S1 Appendix); and lineage turnover rates were 0.305 species lineage^−1^ Ma^−1^ (95% CI = [0.297, 0.312]) and 0.328 species lineage^−1^ Ma^−1^ (95% CI = [0.310, 0.346]) for migratory and nonmigratory lineages, with an average difference of −0.023 species lineage^−1^ Ma^−1^ (95% CI = [−0.043, −0.004]) (Fig Cc in S1 Appendix). Thus, while both analytical approaches indicate that migratory lineages exhibit elevated speciation rates and possibly also net diversification rates, inferences regarding extinction rates, extinction fraction, and lineage turnover were highly sensitive to inclusion of fossil taxa.

### Impacts of extinctions on ungulate functional richness

Migratory species and their unique combination of traits make them functionally important to the ecosystems they inhabit [4–6,20]. Yet it is unclear whether extinct migratory ungulates were functionally unique, characterized by distinct trait combinations, or were largely redundant with extant species. Thus, to assess the potential implications for ecological functioning, we quantified changes in ungulate functional diversity following the extinction of these species. From character data and behavioral reconstructions, we determined each species’ position within 4-dimensional trait space and then calculated the change in ungulate functional richness (*e.g.*, the volume of occupied trait space) when extinct species were lost (Fig 3). Globally, the extinctions of these ungulates resulted in small but consistent declines in the volume of trait space occupied (paired *t* test; *t* = −74.14, *df* = 99, *p-adj* < 0.001), of about 0.8% (median = −0.0082, range = [−0.0084, −0.0080]) across all tree configurations (Fig 3c). Africa experienced larger declines in ungulate functional richness (paired t *t*est; *t* = −252.71, *df* = 99, *p-adj* < 0.001), with the volume of occupied trait space declining by around 2.7% (median = −0.0275, range = [−0.0277, −0.0273]; Fig 3f). In both cases, these declines in functional richness were due to the losses of large-bodied, high latitude, migratory species (Fig 3).

**Fig 3:**
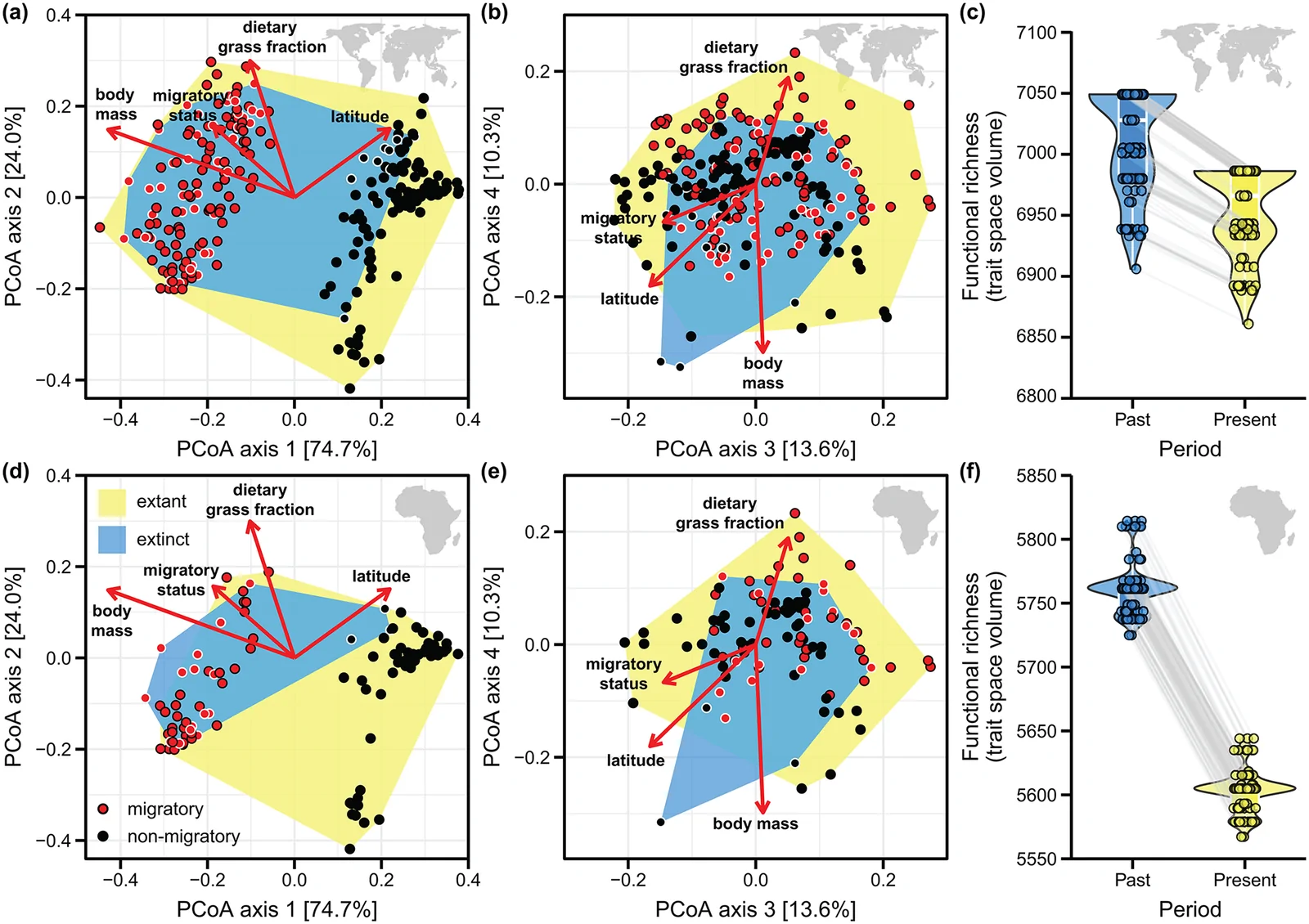
Consequences of losing extinct ungulates for ungulate functional diversity. Principal coordinates analysis (PCoA) results depicting multidimensional trait spaces occupied by **(a, b)** all ungulates globally and **(d, e)** African ungulates only. *Blue polygons* represent the trait space occupied by extinct taxa, while *yellow polygons* represent the trait space occupied by extant taxa. Point colors reflect a species’ migratory status, with *red points* corresponding to migratory species and *black points* corresponding to nonmigratory species. Point outlines reflect extinction status, with *white outlines* corresponding to extinct species and *black outlines* corresponding to extant species. We repeated this for all 100 phylogenetic reconstructions and then evaluated changes in functional richness (*e.g.,* volume of occupied trait space) across all reconstructions. **(c)** Though the extinction of these ungulates negligibly reduced functional richness globally, **(f)** Africa saw more pronounced losses of functional richness. In (c) and (f), g*ray lines* connect functional richness values with (*blue*) and without (*yellow*) extinct taxa for each reconstruction, (c) for all ungulates and (f) for only African species. *White bars* represent the median across reconstructions, *white boxes* encompass the interquartile range (IQR), and the *white whiskers* extend to ±1.5× IQR. Panels (a) and (d) share the same trait axes, as do panels (b) and (e), because African species (d-e) represent a subset of global ungulate species richness (a-b) within the same trait space. Note that the scale of the axes in (c) and (f) is the same, though the axis endpoints differ. Map graphics were generated using the R package ‘maps’ v. 3.4.3 [94]. The data underlying this figure can be found at https://doi.org/10.5061/dryad.jm63xsjsp.

## Discussion

In this study, we evaluated whether migration exposes ungulates to higher extinction risk across evolutionary timescales, thereby rendering it an evolutionarily fragile strategy. By reconstructing the migratory behavior of 51 ungulates species that went extinct through the late Quaternary, we found that (1) extinct ungulates were disproportionately migratory relative to extant ungulates, especially when considering only taxa from Africa, the continent providing the best representation of extinct species in our dataset (Fig 1). Then, using HiSSE modeling to reconstruct macroevolutionary parameters, we found: (2) fossil-inclusive macroevolutionary parameter reconstructions indicated that migratory lineages experienced elevated speciation and extinction rates, as well as elevated lineage turnover and net diversification (Fig 2), though the inclusion of hidden states suggested that other unaccounted-for factors also contributed to parameter heterogeneity (Table C in S1 Appendix); whereas (3) reconstructions based on extant-only phylogenies recovered substantially weaker and partially contradictory signals (Fig C in S1 Appendix). Finally, (4) losses in functional richness from the extinction of migratory ungulates were negligible on a global scale but were more pronounced in Africa (Fig 3).

We intentionally defined migration inclusively here, considering a species as migratory if any population, at any point in time, exhibited seasonal round-trip movements between discrete areas. We took this approach because human activities have substantially altered the expression and detectability of migration by fragmenting landscapes, modifying resource distributions, and in some cases suppressing migratory behavior altogether [8–14]. As a result, some species that are now weakly migratory may have been strongly migratory prior to human impacts and vice versa [3,66]. Defining migration inclusively thus captures the evolutionary capacity for migration rather than its contemporary expression, providing an objective and biologically meaningful framework for macroevolutionary comparison. We recognize that the occurrence of migration may be infrequent or variable in some populations: some species we coded as migratory express this behavior only rarely [17,67]. Nevertheless, rare or facultative behaviors can still influence evolutionary outcomes [67,68], particularly when they are adaptive responses to environmental extremes [69], as migration often is [70,71].

Both our behavioral reconstructions and fossil-inclusive diversification analyses indicated that migratory behavior may increase extinction susceptibility in ungulates. Migratory ungulates tend to be larger-bodied and live at higher latitudes relative to nonmigratory species [17], characteristics which have both been found to increase extinction risk in mammals [52,61,62]. Yet, our fossil-inclusive diversification analyses suggest that migration itself also appears to increase extinction rates, independently of these co-distributed factors [59] (Fig 2b, Table C in S1 Appendix). This elevated extinction risk likely derives from the fact that the benefits of migration are context-dependent: migration is beneficial only when ungulates are able to track predictable spatiotemporal variation in forage quality across landscapes [15,17,25,28]. If the predictability of vegetation growth decreases, or if populations are prevented from accessing needed seasonal resources, then migratory behavior could have net negative impacts on a population [3,14], due to the (often substantial) costs associated with migrating [14,32–36]. In a macroevolutionary context, reduced predictability or access to spatiotemporal variation in forage quality may stem from climate change or the formation of novel physical barriers (*e.g.*, lake formation, shifting river courses, or changing habitat suitability) [3,14,15,19,38]. Thus, our results support the possibility that reliance on migration puts ungulate species at generally greater extinction risk given the inherent variability of the Earth system.

That said, in our diversification analyses, the macroevolutionary signal associated with migration depended strongly on whether extinct taxa were included. Fossil-inclusive HiSSE models consistently supported elevated extinction, speciation, net diversification, and lineage turnover rates within migratory lineages (Fig 2), whereas extant-only analyses recovered weaker and, in some cases, opposing patterns, such as lower extinction rates and extinction fractions for migratory taxa (Fig C in S1 Appendix). These discrepancies likely reflect both the inherent difficulty of estimating extinction dynamics from extant-only phylogenies [64,72] and the fact that extant species represent a nonrandom subset of taxa, such that extinct species may confer critical information about long-term extinction dynamics that are otherwise obscured in extant-only analyses [63,64]. At the same time, incorporating fossils into state-dependent diversification analyses is controversial. Prior work suggests that fossil inclusion can either improve inference [63] or increase false positives [65] depending on the structure of the data and model. Overall, these contrasting results reinforce longstanding concerns about the complexities of estimating extinction dynamics from extant phylogenies alone and demonstrate that interpretations of state-dependent extinction rates depend strongly on the taxonomic completeness of the data analyzed [63,65,72]. Accordingly, we view the broad concordance between our behavioral reconstructions and fossil-inclusive diversification analyses as strong, though not definitive, evidence that migration may increase macroevolutionary vulnerability to extinction in ungulates, such that migration seemingly functions as a high risk-high reward evolutionary strategy overall [19,49].

While providing an additional line of evidence for elevated extinction rates in migratory taxa, our imputation-based reconstructions of migratory behavior are likewise subject to important assumptions and caveats. Because migration is difficult to infer directly from the fossil record (Table B in S1 Appendix), we reconstructed migratory propensity using phylogenetic imputation based on body mass, dietary grass dependence, and latitude, which are traits strongly associated with migration in extant ungulates [17,20]. Although migration was ultimately treated as a binary trait in downstream analyses, our imputation approach effectively assumes continuous variation in migratory propensity. We view this assumption as biologically reasonable because migratory behavior in ungulates exists along a continuum ranging from obligate migration to facultative migration to residency [51,67,73,74], and populations can vary substantially in migratory propensity across space and time [14,38,51]. Nevertheless, we converted imputed migratory propensities to binary values to match the resolution of available data for extant taxa, for which migration is often only documented as present or absent [17]. We recognize that this thresholding approach necessarily collapses uncertainty, although we attempted to retain some degree of uncertainty by replicating all analyses across 100 different tree topologies, which allowed the reconstructed states of some extinct taxa to vary across trees (Fig 1). Indeed, this phylogenetic uncertainty was reflected in our fossil-inclusive diversification analyses, where confidence intervals on parameter estimates were generally wider for migratory than nonmigratory lineages. This pattern likely reflects the fact that the migratory states of extinct taxa varied across tree topologies, whereas those of extant species were fixed: because extinct species were the only taxa whose migratory states varied across trees and were consistently reconstructed as disproportionately migratory, uncertainty about the reconstructed states of extinct species had an outsized influence on parameter reconstructions for migratory lineages. By contrast, when diversification analyses were restricted to extant taxa, migratory species exhibited narrower confidence intervals than nonmigratory lineages, possibly because they exhibit comparatively homogeneous traits relative to the greater character diversity of nonmigratory ungulates [17,20].

We acknowledge also that our imputation-based reconstructions may be influenced by fossil sampling biases. The fossil taxa in this study are unevenly distributed across the ungulate phylogeny (Fig 1a), potentially biasing phylogenetic imputation, and traits associated with migration, especially larger body size and broader geographic ranges, may increase the probability of fossil preservation or discovery [75]. Still, most major ungulate clades are represented in our extinct taxa set. Additionally, the quantitatively stronger signal recovered within African taxa, where fossil representation is comparatively complete [54,55], suggests that preservation differences alone cannot account for the observed relationship between migration and elevated extinction rates.

Although the extinction of these ungulates produced relatively modest reductions in global functional richness, functional losses were more pronounced at continental scales (Fig 3). Extinct migratory taxa thus occupied distinct regions of trait space, exhibiting character combinations not represented among locally occurring extant ungulates. Because migration covaries strongly with other traits [17,20], losing migratory species results in concomitant declines in these other functional dimensions. The resultant contractions of ungulate trait space have certainly had cascading consequences for a variety of ecosystem processes strongly associated with these traits [4,5]. Overall, while negligible global declines in functional richness suggest that global ungulate assemblages retain substantial redundancy [76], the stronger continental-scale losses indicate that local and regional ecosystems may be considerably more vulnerable to the disappearance of migratory species [6,12].

Across both fossil-inclusive (Fig 2) and extant-only (Fig C in S1 Appendix) diversification analyses, we found that migratory lineages consistently exhibited elevated speciation rates relative to nonmigratory lineages, suggesting that migration promotes lineage formation over macroevolutionary timescales. Net diversification rates likewise tended to be higher for migratory lineages, although this pattern was less consistent in both sets of analyses. Such speciation and diversification patterns may reflect the fact that disruptions of migratory pathways often coincide with major population crashes [8–14], and the resultant losses of genetic diversity can lead either to extinction [30,31] or to speciation if small populations can survive long enough to diverge genetically [42–45]. Migration might further promote speciation by introducing barriers to gene flow: differences in migratory routes or migration phenology between populations could act as reproductive barriers, genetically isolating populations and leading to speciation [42–48]. Thus, our results indicate that migration simultaneously exposes ungulates to higher extinction risks while promoting speciation. Consequently, migratory lineages may experience faster turnover of relatively ephemeral species, emerging frequently but persisting briefly before going extinct [49]. This precarious balance between heightened rates of extinction and speciation within migratory lineages underscores the fragility of migration as an evolutionary strategy in an unpredictable world.

While elevated speciation rates seem to have historically compensated for heightened extinction within migratory lineages, as evidenced by the generally positive effects of migration on net diversification, this balance between extinction and speciation may be shifting during the Anthropocene, due to intensifying human impacts and rapid climate change [1–3]. Not only are the same processes that have been disrupting migratory routes for millennia still at work today, but humans are disrupting migratory systems much more efficiently, thereby putting this already-precarious evolutionary strategy at even greater risk. Humans are rapidly altering the distribution, predictability, and accessibility of spatiotemporal gradients of plant growth by constructing physical barriers, converting land to agriculture, overexploiting migratory populations, and contributing to anthropogenic climate change [2,3,12,38]. For example, habitat degradation and the construction of fences in the Athi-Kaputiei Plains have obstructed local wildebeest (*Connochaetes taurinus*) migration routes, causing their population to plummet [9,12]; it is feared that road construction in the Serengeti ecosystem might similarly disrupt the multispecies migration therein [77], triggering collapses of those migratory populations [7]. Also, the iconically migratory saiga antelope (*Saiga tatarica*) declined by >95% after the collapse of the Soviet Union due to uncontrolled hunting for meat and horns and is only now beginning to recover [11,12]. If migratory lineages are indeed characterized by elevated turnover over macroevolutionary timescales, as our fossil-inclusive diversification analyses suggest, then contemporary anthropogenic pressures may increasingly disrupt the balance between lineage formation and extinction. Thus, based on the evidence we recover here, and echoing the advice of many others [12,50], we argue that investing in the conservation of migratory species now is more critical than ever.

Together, our results suggest that migration has diverse macroevolutionary consequences for ungulates, simultaneously promoting lineage formation while potentially exacerbating vulnerability to extinction. Additionally, the contrasting results obtained from fossil-inclusive and extant-only diversification analyses highlight the importance but also the difficulty of inferring extinction dynamics from fossil-inclusive phylogenies, particularly for behavioral traits that are difficult to reconstruct directly from the fossil record. Although uncertainty remains about the precise effects of migration on some diversification parameters, our results demonstrate that migration has played a central role in shaping ungulate evolution.

## Materials and methods

### Collating ungulate character data

We first identified extinct species suitable for inclusion in our analyses. We included any ungulate taxa (Perissodactyla and Artiodactyla, excluding Cetacea) that have persisted through the late Quaternary (late Pleistocene and Holocene) within extant families, as families without extant representatives are difficult to place phylogenetically. This limited the available species pool to taxa that arose within the Neogene (<23 Ma, the interval during which phylogenetic evidence suggests that migratory behavior in ungulates first evolved [17]).

Migratory behavior in extant ungulates is strongly positively associated with other characters, specifically body mass, dietary grass fraction, and latitude: these three characters together explain ~44% of variation in whether or not ungulate species are capable of migration [17]. We therefore used the correlations between migration and these characters within extant species to infer migratory behavior in extinct taxa (see Data analyses). Data on the migratory habits of extant ungulates, as well as their diets, body masses, and latitudinal niches, were available from [17]. Migratory behavior varies substantially within species and across populations (with some populations of a species being entirely nonmigratory and others almost completely migratory [51]) but is not well-catalogued at population scale. For this reason, and because anthropogenic factors such as physical barriers and habitat alterations have modified the distribution of migratory behavior in many taxa [2,3,50], migratory behavior was coded as binary: a species was considered migratory if it was explicitly described as such in published literature or if any population, past or present, exhibited seasonal round-trip movements between discrete ranges. While this inclusive classification may overestimate the importance of migration for some taxa or underestimate it for data-deficient ones, it provides a consistent framework for macroevolutionary comparison across taxa and time.

We then compiled data on the dietary grass fractions, body masses, and latitudinal range centroids of extinct ungulates. We estimated latitudinal range centroids from fossil locality data in the Paleobiology Database (http://paleodb.org), refined with expert knowledge from additional published sources. Latitude provides a relative and internally consistent proxy for the magnitude of seasonality to which species are exposed [20], particularly given that Earth’s climate has varied markedly through geological time and fine-scale paleoclimatic reconstructions sufficient to infer migratory behavior are unavailable [17,78]. Body mass estimates were derived from various sources via targeted literature searches (S1 Data); estimates were generally derived from lineage-specific extrapolations of body mass from the skeletal allometry of extant relatives. Dietary grass fraction data were likewise compiled from various published sources. These data were predominately estimated via stable isotope analysis of fossilized material (*e.g*., tooth enamel) or DNA metabarcoding of coprolites (see S1 Data for further details). Values range from 0 (a diet comprising no grass) to 1 (a diet comprising exclusively grass). Reliable quantitative diet data were available for a limited number of species (*n* = 51) and served as the biggest barrier to the inclusion of taxa.

One of the most widely used proxies for reconstructing herbivore dietary grass fraction, stable carbon isotope data, works best in the tropics and especially in tropical Africa, where nearly all grasses are C_4_-photosynthesizing and grasses consequently have a unique isotopic signature [53,79]; outside of Africa, especially at higher latitudes, both C_3_- and C_4_-photosynthesizing grasses are prevalent, so stable carbon isotopes cannot readily differentiate grasses from nongrasses [79]. Accordingly, dietary grass estimates were available for the majority (>75%) of extinct African ungulates that met our other inclusion criteria [53], such that African taxa are well-represented in this study. We therefore categorized both extinct and extant species as either African or nonAfrican: for extinct species, we determined whether a species occurred in Africa from the aforementioned fossil locality data from the Paleobiology Database; for extant species, we determined whether a species occurred in Africa from species range maps curated by the IUCN [80]. This allowed us to repeat all analyses on only the African taxa as a ‘best quality’ data subset (see Data analyses).

Finally, to validate our inferred behavioral reconstructions, we conducted a literature review to identify evidence of migratory behavior in extinct taxa (Table B in S1 Appendix). For each species, we searched its scientific name along with the term ‘migrat*’ in Google Scholar, then reviewed all returned sources for indications of migratory behavior and the nature of the supporting evidence. We found robust fossil evidence for or against the existence of migratory behavior for 16 of the 51 extinct species, and weaker evidence for an additional four species (Table B in S1 Appendix).

### Placing extinct taxa in the ungulate phylogeny

We then incorporated the 51 extinct ungulates with all available character data into a published set of mammal-wide phylogenies [52]. For our analyses, we used 100 different tree configurations from the published set of birth-death node-dated completed trees, in which divergence times among major lineages were estimated using fossil-calibrated backbones [52]. These phylogenies were constructed by combining a 31-gene supermatrix with Bayesian inference to generate time-scaled backbone and patch trees [52]. Species lacking genetic data were placed using taxonomic constraints and branch lengths inferred from local birth-death models [52]. The full trees contained 5,911 mammal species, including 13 recently extinct ungulate species for which genetic data were available [52]. For our analyses, we pruned each tree in the set down to only ungulates (13 extinct and 207 extant species).

The remaining 38 extinct taxa not already included in the phylogeny set were grafted onto all 100 phylogenetic trees using the ‘bind.tip’ function in the ‘phytools**’** R package [81], following the procedure described in [82]. For these 38 extinct species, we determined their phylogenetic placements using a multi-step approach specific to each species (Fig 1a; see S1 Data). First, we used Linnaean taxonomic classifications as a guiding framework. If a fossil species was confidently assigned to a particular genus, it was positioned within the crown or stem of that genus accordingly. Where available, we consulted the most recent morphological, molecular, or combined-data phylogenies for each extinct species to guide more precise placements. In such cases, extinct taxa were inserted on a case-by-case basis based on their inferred evolutionary relationships. For example, the three extinct antilocaprid species *Capromeryx furcifer*, *Stockoceros conklingi*, and *Tetrameryx shuleri*, all members of the extinct tribe Stockoceratini, were placed as a polytomy with respect to one another, and this polytomy was grafted as sister to their closest living relative, *Antilocapra americana*. In cases where finer-scale phylogenetic resolution was unavailable, taxa were conservatively placed as polytomies within the broader crown group of the relevant genus. For instance, no detailed phylogenetic information could be found for the extinct species *Antidorcas australis* and *A. bondi*, and so both were added as a polytomy with the extant *A. marsupialis*. Full details of fossil species placements, along with relevant citations, are provided in S1 Data.

Once placements were determined, we anchored each extinct taxon within the tree by defining its branch length as the difference between its first and last appearances in the fossil record: the first occurrence was used as the node age (representing the branching point from its closest known relative), and the last occurrence was treated as the tip age. This approach allowed us to consistently define branch lengths across species, though we recognize that using observed stratigraphic ranges of fossil species necessarily truncates branch lengths at both ends; despite the fact that our approach underestimates branch lengths, the error it introduces should be negligible relative to the substantial age of ungulates overall (~60 Ma crown age [17,52]).

Given that many placements produced polytomous clades, we used the ‘fix.poly’ function from the ‘RRphylo**’** R package to resolve these polytomies into dichotomies by assigning arbitrarily small branch lengths (0.0001) to newly created internodes [83]. This entire process was repeated across all 100 trees so we could propagate phylogenetic uncertainty through our subsequent analyses.

### Data analyses

Data analyses were performed in R v. 4.3.2 [84]. First, we phylogenetically imputed the migratory habits of extinct taxa from the character data we compiled, leveraging the robust correlations between those characters within extant taxa [17,20], using ‘phylopars’ in the R package ‘Rphylopars’ [85]. Prior to imputation, we compared various models of trait evolution (Brownian motion, lambda, kappa, early burst, and star) across all 100 phylogenetic trees using the small-sample-size-corrected Akaike Information Criterion (AIC_c_) (Table A in S1 Appendix). Lambda (λ) models of migratory evolution consistently yielded the best fit (*i.e.,* lowest AIC_c_) and were thus used for imputation for all 100 trees, with λ values estimated separately for each tree topology (Table A in S1 Appendix).

Migration was treated as a continuous variable for imputation purposes, with ‘1′ being migratory and ‘0′ being nonmigratory. Methodologically, recent simulation work suggests that treating discrete traits as continuous for the purposes of phylogenetic imputation generally yields low error rates [86], particularly when phylogenetic structure is strong, as it is here. Biologically, this approach also reflects the fact that migratory behavior within ungulates is widely recognized as existing along a continuum from obligate migration to facultative migration to residency [51,67,73,74]. Accordingly, the imputed values can be interpreted as estimates of migratory propensity in extinct taxa. To match the binary resolution of the available behavioral data for extant species, however, values from imputation were then converted to binary: values < 0.5 were transformed to 0s (probable nonmigrant) and values ≥ 0.5 were transformed to 1s (probable migrant). We did this across all 100 tree configurations, such that the migratory habits of some species with intermediate migration probabilities varied depending on the topology of the tree (Fig 1). To evaluate the accuracy of our imputed reconstructions, we compared them against fossil-based evidence of migratory behavior compiled from the literature (Table B in S1 Appendix).

We then compared the prevalence of migratory behavior between extinct and extant species across all 100 reconstructions using phylogenetic generalized linear modeling (phyloGLM; Fig 1b, Fig A in S1 Appendix). Because migratory status was binary, we used logistic regression, implemented via the ‘phylolm’ R package [87]. We repeated this comparison on only African species (Fig 1c, Fig A in S1 Appendix).

Next, we used model selection to determine the most appropriate framework for reconstructing macroevolutionary rates. For each tree, we compared the fits of three models: a Binary State Speciation and Extinction (BiSSE) model, a HiSSE model, and a character-independent diversification model with two hidden states (CID-2). All three models allow for the assessment of whether a binary trait (in this case, ‘migratory’ and ‘nonmigratory’) affects diversification parameters, while also accounting for transitions between character states [59,60]. HiSSE extends BiSSE by incorporating unobserved (hidden) states with potentially distinct evolutionary dynamics [59], reducing the risk of falsely attributing variation in diversification parameters to the observed trait (type I error) [59,60]. CID-2 models include only hidden states and assume that diversification is independent of observed traits [59,60].

We implemented all models using the R package ‘hisse’ [88]; it is the only currently available implementation of HiSSE modeling that allows for the presence of fossil (*i.e.,* extinct) taxa [88]. The effects of incorporating fossil taxa into state-dependent diversification analyses remain uncertain, however: some studies suggest that fossil inclusion can improve parameter estimation and reduce type I error by alleviating under-sampling bias inherent to extant-only phylogenies [63,64,72], whereas others have found little benefit or even increased false-positive rates under certain model structures and sampling conditions [65]. Consequently, to assess the sensitivity of inferred evolutionary dynamics to the inclusion of fossils, we replicated all diversification analyses both with (‘fossil-inclusive’) and without (‘extant-only’) extinct taxa.

For HiSSE, we specified a model with two hidden states, one per observed state, yielding four combined states (Figs E and F in S1 Appendix). For CID-2, we specified two hidden states, mirroring the structure of the HiSSE model. Across all model types, we allowed turnover rates and extinction fractions to vary by state but held sampling fractions constant across states. Though sampling fractions did not vary by state, we employed distinct sampling fractions for fossil-inclusive (0.1) and extant-only (0.75) analyses: for fossil-inclusive analyses, sampling fraction was calculated from the proportion of all ungulate taxa meeting our inclusion criteria (late Quaternary through present taxa within extant families) represented in our phylogeny (258/1923 species; 13%), based on unique species recognized in the NOW database [89]; for extant-only analyses, sampling fraction was calculated from the proportion of extant ungulate species recognized by the IUCN represented in the phylogeny (207/259 species [243 Artiodactyla, 16 Perissodactyla]; 80%) [80]. Per prior simulation work indicating that conservatively underestimating sampling fractions reduces false positives in state-dependent diversification analyses [72], we rounded both values down to 0.1 and 0.75, respectively.

The fits of BiSSE, HiSSE, and CID-2 models were then evaluated by comparing AIC_c_ values for each of our 100 phylogenies. For the majority of trees across both fossil-inclusive (93/100 trees) and extant-only (88/100 trees) analyses, HiSSE models outperformed both BiSSE and CID-2 models (*i.e.,* lower AIC_c_), indicating that HiSSE was generally the most appropriate model structure for reconstructing macroevolutionary dynamics (Tables C and E in S1 Appendix). We therefore used macroevolutionary parameter estimates derived from HiSSE models in all subsequent analyses, for both fossil-inclusive and extant-only analyses.

HiSSE models generated distinct estimates of speciation, extinction, lineage turnover, net diversification (in units of species per lineage per million years; species lineage^−1^ Ma^−1^) and extinction fraction (unitless) at each tip in the phylogeny, such that each estimate was associated with character data. Although we report resultant absolute parameter estimates here for completeness (Fig 2 and Fig C in S1 Appendix), these values can be sensitive to model specification and parameterization and should therefore be interpreted cautiously [59,63–65,72]. Accordingly, we focus our interpretation primarily on the direction and consistency of differences between migratory and nonmigratory lineages across phylogenetic tree topologies, rather than on the precise magnitude of reconstructed parameter values. We used logistic phyloGLMs to test whether macroevolutionary parameters differed significantly between migratory and nonmigratory lineages across all 100 tree configurations (Fig B in S1 Appendix). We also compared the distributions of mean rates (averaged within each tree) between migratory and nonmigratory species (Fig 2).

To assess whether migration itself, rather than a correlated trait, accounted for differences in diversification rates, we compared the AIC_c_ values of logistic phyloGLMs with migration as a predictor to those of phylogenetic linear models (phyloLMs) with body size, grass dependence, or latitude as a predictor (Tables D and F in S1 Appendix). All models were implemented using the ‘phylolm’ R package [87], as above. This comparison was conducted for all five macroevolutionary parameter classes (speciation, extinction, lineage turnover, net diversification, and extinction fraction) estimated by the HiSSE models.

Finally, we evaluated the consequences of losing extinct ungulate species for ungulate functional richness. Functional richness is a measure of the multidimensional volume of trait space occupied [76,90,91]. We calculated ungulate functional richness with and without extinct species, both globally and for Africa only, from species character data (dietary grass fraction, body mass, latitude, and migratory habit) (Fig 3). We calculated a Gower distance matrix of pairwise functional (dis-)similarity between all ungulate species (extinct and extant both), using the ‘gowdis’ function in the ‘FD’ R package [90]. Gower distance is a widely used and highly flexible distance metric that can incorporate a diversity of variable types [76,90,91]. Each character was given equal weight in our Gower distance calculations. As above, we repeated this across all 100 tree conformations, resulting in 100 Gower distance matrices, one for each tree.

For each Gower distance matrix, we then visualized Gower distances between species in multidimensional trait space (Fig 3) using ‘wcmdscale’ in the ‘vegan’ R package [92], which conducts principal coordinates analysis (PCoA) on the Gower distance matrix. We used the first four axes of the PCoA (Fig 3), because our Gower distance calculations were based on only four characters and because analyses of real and simulated data suggest that including more than four axes can distort functional relationships [76,93]. For each of the 100 Gower distance matrices, we calculated the functional richness (*i.e.*, volume of trait space occupied) of ungulates globally and for African ungulates specifically with and without extinct taxa, using ‘dbFD’ in the R package ‘FD’ [90]. We then compared functional richness with and without extinct taxa with separate paired *t*-tests for global (Fig 3c) and Africa-only (Fig 3f) functional richness data. Finally, we visualized the relationship between PCoA axes and the four characters used to generate Gower distance matrices with ‘envfit’ and 999 permutations in the R package ‘vegan’ [92].

## Supporting information

S1 Appendix All supplementary tables and figures referenced in the main text. Contains Tables A–F and Figs A–F.
(DOCX)

S1 Data Data on extinct ungulate species. Tab ‘Data’ contains the data used for phylogenetic placements (including sister taxa and dates of first and last fossil record occurrences) as well as data used for phylogenetically imputing migration status (including latitudinal range centroid, body mass, and dietary grass fraction); tab ‘Sources’ contains references to all literature from which data were compiled.
(XLSX)

S2 Data Ungulate phylogenetic tree set including extinct species. The tree set includes 100 different ungulate phylogenetic tree topologies employed in analyses.
(NEX)

## Abbreviations

BiSSE: Binary State Speciation and Extinction
HiSSE: Hidden State Speciation and Extinction
IQR: interquartile range
Ma: million years
PCoA: principal coordinates analysis.
