# Supplementary material for: Phylogenetic evidence that migratory behavior increases ungulate extinction rates
Source: PLoS Biol. 2026 Nov 5;24(11):e3004072. doi: 10.1371/journal.pbio.3004072 (PMC13739097; doi:10.1371/journal.pbio.3004072)
Supplement: Supplementary file 1 [file pbio.3004072.s001.docx]

**SUPPORTING INFORMATION**

**TITLE: Phylogenetic evidence that migratory behavior increases ungulate extinction rates**

**AUTHORS:** Joel O. Abraham^1*^, Kaedan O’Brien^2^, Nathan S. Upham^3^, Brett R. Jesmer^4^, Faysal Bibi^5^, J. Tyler Faith^6,7^

**AFFILIATIONS:**

^1^Department of Ecology and Evolutionary Biology, Princeton University, Princeton, New Jersey, United States of America

^2^Department of Anthropology, SUNY Oneonta, Oneonta, New York, United States of America

^3^School of Life Sciences, Arizona State University, Tempe, Arizona, United States of America

^4^Department of Fish and Wildlife Conservation, Virginia Tech, Blacksburg, Virginia, United States of America

^5^Museum für Naturkunde, Leibniz Institute for Evolution and Biodiversity Science, Berlin, Germany

^6^Department of Anthropology, University of Utah, Salt Lake City, Utah, United States of America

^7^Natural History Museum of Utah, University of Utah, Salt Lake City, Utah, United States of America

**^*^** joeloa@princeton.edu;

| **Table A. Model selection results for identifying the most appropriate model of migratory evolution.** For each tree, we compared the fits of Brownian motion, lambda (λ), kappa (κ), early burst, and star phylogeny (*e.g.,* lacking phylogenetic structure) models of trait evolution using the small-sample-size-corrected Akaike Information Criterion (AIC_c_). Lambda models of trait evolution were preferred for all 100 tree topologies, with λ estimates ranging between 0.880 and 0.912 across trees. The table reports ΔAIC_c_ values and λ estimates for each tree. | | | | | | |
| --- | --- | --- | --- | --- | --- | --- |
| Tree number | Brownian motion | Lambda (λ) | Kappa (κ) | Early burst | Star phylogeny | Estimated lambda (λ) value |
| 1 | 1298.5 | 0.0 | 60.9 | 1300.5 | 451.3 | 0.896 |
| 2 | 1282.3 | 0.0 | 63.6 | 1284.3 | 448.5 | 0.898 |
| 3 | 1277.5 | 0.0 | 52.8 | 1279.5 | 444.0 | 0.901 |
| 4 | 1332.7 | 0.0 | 65.8 | 1334.7 | 456.0 | 0.899 |
| 5 | 1228.1 | 0.0 | 59.3 | 1230.1 | 457.2 | 0.906 |
| 6 | 1319.9 | 0.0 | 68.9 | 1314.6 | 457.2 | 0.887 |
| 7 | 1185.9 | 0.0 | 59.5 | 1187.9 | 450.2 | 0.907 |
| 8 | 1272.9 | 0.0 | 61.0 | 1274.9 | 448.8 | 0.888 |
| 9 | 1243.1 | 0.0 | 63.0 | 1245.1 | 450.4 | 0.903 |
| 10 | 1305.2 | 0.0 | 63.7 | 1307.2 | 442.5 | 0.891 |
| 11 | 1388.3 | 0.0 | 63.5 | 1390.3 | 455.9 | 0.890 |
| 12 | 1246.4 | 0.0 | 66.6 | 1248.4 | 453.2 | 0.911 |
| 13 | 1167.9 | 0.0 | 58.7 | 1169.9 | 453.5 | 0.903 |
| 14 | 1309.6 | 0.0 | 60.3 | 1311.6 | 453.9 | 0.903 |
| 15 | 1242.2 | 0.0 | 60.4 | 1244.2 | 451.9 | 0.909 |
| 16 | 1268.3 | 0.0 | 66.8 | 1270.3 | 458.8 | 0.902 |
| 17 | 1266.6 | 0.0 | 49.3 | 1268.6 | 441.2 | 0.892 |
| 18 | 1240.6 | 0.0 | 53.0 | 1242.6 | 453.4 | 0.900 |
| 19 | 1321.3 | 0.0 | 73.8 | 1323.3 | 448.5 | 0.894 |
| 20 | 1294.0 | 0.0 | 57.2 | 1296.0 | 448.5 | 0.889 |
| 21 | 1293.1 | 0.0 | 62.5 | 1295.1 | 444.4 | 0.895 |
| 22 | 1192.6 | 0.0 | 62.4 | 1194.6 | 449.9 | 0.899 |
| 23 | 1198.7 | 0.0 | 62.1 | 1200.7 | 448.4 | 0.902 |
| 24 | 1227.7 | 0.0 | 69.5 | 1229.7 | 452.7 | 0.890 |
| 25 | 1255.3 | 0.0 | 69.4 | 1257.3 | 458.4 | 0.897 |
| 26 | 1366.8 | 0.0 | 66.8 | 1368.8 | 449.3 | 0.891 |
| 27 | 1300.3 | 0.0 | 73.4 | 1302.3 | 457.2 | 0.891 |
| 28 | 1237.1 | 0.0 | 66.5 | 1239.1 | 452.8 | 0.907 |
| 29 | 1189.2 | 0.0 | 63.2 | 1191.2 | 456.9 | 0.902 |
| 30 | 1245.9 | 0.0 | 70.7 | 1247.9 | 453.5 | 0.902 |
| 31 | 1261.5 | 0.0 | 50.3 | 1263.5 | 445.3 | 0.901 |
| 32 | 1390.4 | 0.0 | 54.7 | 1392.4 | 447.3 | 0.888 |
| 33 | 1215.5 | 0.0 | 60.5 | 1217.5 | 447.1 | 0.909 |
| 34 | 1328.4 | 0.0 | 70.6 | 1330.4 | 454.1 | 0.889 |
| 35 | 1311.3 | 0.0 | 66.7 | 1313.3 | 448.0 | 0.895 |
| 36 | 1221.2 | 0.0 | 61.9 | 1223.2 | 451.7 | 0.904 |
| 37 | 1164.4 | 0.0 | 69.2 | 1166.4 | 452.8 | 0.895 |
| 38 | 1288.9 | 0.0 | 60.9 | 1290.9 | 450.7 | 0.906 |
| 39 | 1142.5 | 0.0 | 53.1 | 1144.5 | 439.4 | 0.904 |
| 40 | 1112.0 | 0.0 | 66.8 | 1114.0 | 450.8 | 0.912 |
| 41 | 1290.1 | 0.0 | 65.0 | 1248.4 | 446.9 | 0.904 |
| 42 | 1247.5 | 0.0 | 61.1 | 1249.5 | 445.4 | 0.895 |
| 43 | 1303.8 | 0.0 | 59.1 | 1305.8 | 450.9 | 0.906 |
| 44 | 1378.3 | 0.0 | 62.8 | 1380.3 | 449.4 | 0.892 |
| 45 | 1182.1 | 0.0 | 60.7 | 1184.1 | 451.7 | 0.903 |
| 46 | 1320.8 | 0.0 | 65.1 | 1322.8 | 453.9 | 0.908 |
| 47 | 1220.4 | 0.0 | 59.2 | 1222.4 | 450.1 | 0.898 |
| 48 | 1173.8 | 0.0 | 63.0 | 1175.8 | 448.3 | 0.902 |
| 49 | 1374.0 | 0.0 | 62.8 | 1376.0 | 444.3 | 0.904 |
| 50 | 1199.1 | 0.0 | 60.6 | 1201.1 | 448.0 | 0.899 |
| 51 | 1196.8 | 0.0 | 65.3 | 1198.8 | 451.3 | 0.908 |
| 52 | 1381.2 | 0.0 | 66.3 | 1383.2 | 443.6 | 0.894 |
| 53 | 1289.7 | 0.0 | 64.5 | 1291.7 | 444.9 | 0.893 |
| 54 | 1241.6 | 0.0 | 60.5 | 1243.6 | 449.7 | 0.897 |
| 55 | 1336.6 | 0.0 | 72.3 | 1338.6 | 457.1 | 0.894 |
| 56 | 1235.3 | 0.0 | 65.1 | 1237.3 | 453.7 | 0.908 |
| 57 | 1227.0 | 0.0 | 67.9 | 1229.0 | 455.2 | 0.890 |
| 58 | 1281.9 | 0.0 | 68.4 | 1283.9 | 453.1 | 0.907 |
| 59 | 1168.5 | 0.0 | 55.2 | 1170.5 | 448.4 | 0.902 |
| 60 | 1307.2 | 0.0 | 74.5 | 1309.2 | 455.5 | 0.886 |
| 61 | 1219.5 | 0.0 | 59.4 | 1221.5 | 446.3 | 0.899 |
| 62 | 1397.3 | 0.0 | 66.7 | 1399.3 | 449.5 | 0.892 |
| 63 | 1269.6 | 0.0 | 74.1 | 1271.6 | 453.9 | 0.897 |
| 64 | 1159.3 | 0.0 | 61.4 | 1161.3 | 450.2 | 0.895 |
| 65 | 1269.5 | 0.0 | 63.9 | 1271.5 | 456.6 | 0.906 |
| 66 | 1268.8 | 0.0 | 65.0 | 1270.8 | 447.9 | 0.897 |
| 67 | 1236.2 | 0.0 | 61.9 | 1238.2 | 442.5 | 0.900 |
| 68 | 1322.2 | 0.0 | 57.8 | 1324.2 | 450.3 | 0.899 |
| 69 | 1256.5 | 0.0 | 64.2 | 1258.5 | 444.0 | 0.894 |
| 70 | 1154.8 | 0.0 | 61.9 | 1156.8 | 452.3 | 0.906 |
| 71 | 1296.4 | 0.0 | 52.7 | 1298.4 | 448.9 | 0.895 |
| 72 | 1258.4 | 0.0 | 61.3 | 1260.4 | 448.1 | 0.892 |
| 73 | 1213.7 | 0.0 | 65.8 | 1215.7 | 455.9 | 0.897 |
| 74 | 1372.6 | 0.0 | 58.2 | 1374.6 | 446.7 | 0.897 |
| 75 | 1294.2 | 0.0 | 63.9 | 1296.2 | 448.1 | 0.899 |
| 76 | 1344.1 | 0.0 | 63.9 | 1346.1 | 446.4 | 0.892 |
| 77 | 1298.6 | 0.0 | 73.1 | 1300.6 | 455.9 | 0.881 |
| 78 | 1326.0 | 0.0 | 58.6 | 1328.0 | 452.4 | 0.894 |
| 79 | 1379.6 | 0.0 | 60.1 | 1381.4 | 446.4 | 0.893 |
| 80 | 1774.0 | 0.0 | 78.7 | 1776.0 | 449.8 | 0.888 |
| 81 | 1296.9 | 0.0 | 69.7 | 1298.9 | 449.4 | 0.905 |
| 82 | 1328.0 | 0.0 | 60.0 | 1330.0 | 447.7 | 0.900 |
| 83 | 1250.5 | 0.0 | 62.0 | 1252.5 | 454.9 | 0.895 |
| 84 | 1261.0 | 0.0 | 64.1 | 1263.0 | 444.5 | 0.891 |
| 85 | 1261.4 | 0.0 | 65.1 | 1263.4 | 447.4 | 0.887 |
| 86 | 1299.0 | 0.0 | 58.9 | 1303.3 | 447.2 | 0.900 |
| 87 | 1280.4 | 0.0 | 66.4 | 1282.4 | 460.2 | 0.906 |
| 88 | 1206.0 | 0.0 | 53.2 | 1208.0 | 444.9 | 0.898 |
| 89 | 1309.3 | 0.0 | 67.0 | 1311.3 | 444.9 | 0.894 |
| 90 | 1271.0 | 0.0 | 63.8 | 1273.0 | 443.0 | 0.895 |
| 91 | 1232.7 | 0.0 | 71.1 | 1234.4 | 455.1 | 0.896 |
| 92 | 1271.2 | 0.0 | 62.9 | 1273.2 | 452.0 | 0.891 |
| 93 | 1323.5 | 0.0 | 72.8 | 1325.5 | 454.0 | 0.898 |
| 94 | 1228.3 | 0.0 | 62.4 | 1230.3 | 447.2 | 0.899 |
| 95 | 1321.6 | 0.0 | 69.1 | 1323.6 | 452.7 | 0.885 |
| 96 | 1311.1 | 0.0 | 68.1 | 1313.2 | 451.6 | 0.897 |
| 97 | 1209.8 | 0.0 | 62.4 | 1211.8 | 457.0 | 0.905 |
| 98 | 1301.7 | 0.0 | 62.6 | 1303.7 | 457.3 | 0.880 |
| 99 | 1291.2 | 0.0 | 52.6 | 1293.2 | 444.9 | 0.895 |
| 100 | 1276.1 | 0.0 | 51.1 | 1278.1 | 444.3 | 0.902 |
| **Median**  **[Range]** | **1271.1**  **[1112.0,**  **1774.0]** | **0.0**  **[0, 0]** | **63.0**  **[49.3, 78.7]** | **1272.3**  **[1114.0,**  **1776.0]** | **450.2**  **[439.4,**  **460.2]** | **0.898**  **[0.880, 0.912]** |

| **Table B. Comparison of migratory behavioral reconstructions based on phylogenetic imputation and fossil evidence.** Numbers in brackets indicate the number of tree topologies for which migratory behavior was inferred. Species with NA values lacked literature investigating their migratory behavior. Instances with reliable fossil evidence are **bolded.** Instances where fossil evidence does not align with imputed migratory statuses are underlined. | | | | |
| --- | --- | --- | --- | --- |
| Species | Inferred migratory habit | Evidence of migration? | Nature of evidence | Source |
| *Aepyceros* sp. | Migratory [100/100] | **No*** | Serially sampled strontium stable isotopes from tooth enamel | [1] |
| *Antidorcas australis* | Migratory [100/100] | NA | NA | NA |
| *Antidorcas bondi* | Migratory [100/100] | **Yes** | Demography reconstructions from fossil remains | [2] |
| *Bos antiquus* | Migratory [100/100] | **Yes** | Demography reconstructions from fossil remains | [3] |
|  |  | **Yes** | Serially sampled carbon and oxygen stable isotopes from tooth enamel | [4] |
| *Bos latifrons* | Migratory [100/100] | **Yes** | Serially sampled oxygen stable isotopes from tooth enamel | [5] |
| *Bootherium bombifrons* | Migratory [100/100] | NA | NA | NA |
| *Bos primigenius* | Migratory [100/100] | NA | NA | NA |
| *Bos priscus* | Migratory [100/100] | **Yes** | Serially sampled strontium, oxygen, and carbon stable isotopes from tooth enamel | [6] |
| *Camelops hesternus* | Migratory [100/100] | **Yes** | Serially sampled strontium and oxygen stable isotopes from tooth enamel | [7] |
| *Capromeryx furicifer* | Non-migratory [0/100] | NA | NA | NA |
| *Cervus astylodon* | Non-migratory [0/100] | NA | NA | NA |
| *Coelodonta antiquitatis* | Migratory [100/100] | **No*** | Distribution of fossil localities | [8] |
| *Damaliscus hypsodon* | Migratory [100/100] | Possible | Serially sampled strontium stable isotopes from tooth enamel | [1] |
|  |  | **Yes** | Demography reconstructions from fossil remains | [9,10] |
| *Damaliscus niro* | Migratory [100/100] | NA | NA | NA |
| *Equus capensis* | Migratory [100/100] | NA | NA | NA |
| *Equus conversidens* | Migratory [100/100] | Possible (species uncertain) | Serially sampled strontium stable isotopes from tooth enamel | [11] |
|  |  | **No*** | Serially sampled oxygen and strontium stable isotopes from tooth enamel | [12] |
| *Equus fraternus* | Migratory [100/100] | Possible (species uncertain) | Serially sampled strontium stable isotopes from tooth enamel | [11] |
| *Equus lambei* | Migratory [100/100] | Possible (species uncertain) | Serially sampled strontium stable isotopes from tooth enamel | [11] |
| *Equus lenensis* | Migratory [100/100] | NA | NA | NA |
| *Equus mauritanicus* | Migratory [100/100] | NA | NA | NA |
| *Equus neogeus* | Migratory [100/100] | NA | NA | NA |
| *Equus occidentalis* | Migratory [100/100] | Possible (species uncertain) | Serially sampled strontium stable isotopes from tooth enamel | [11] |
|  |  | **No*** | Serially sampled carbon and oxygen stable isotopes from tooth enamel | [4] |
| *Equus ovodovi* | Migratory [100/100] | NA | NA | NA |
| *Equus scotti* | Migratory [100/100] | NA | NA | NA |
| *Hemiauchenia macrocephala* | Migratory [100/100] | NA | NA | NA |
| *Hemiauchenia paradoxa* | Migratory [100/100] | NA | NA | NA |
| *Hippidion devillei* | Migratory [100/100] | NA | NA | NA |
| *Hippidion principale* | Migratory [100/100] | NA | NA | NA |
| *Hippidion saldiasi* | Migratory [100/100] | NA | NA | NA |
| *Hippopotamus lemerlei* | Migratory [95/100] | NA | NA | NA |
| *Hippopotamus madagascariensis* | Non-migratory [13/100] | NA | NA | NA |
| *Hippotragus leucophaeus* | Migratory [100/100] | **Yes** | Demography reconstructions from fossil remains | [13] |
| *Megaceroides algericus* | Non-migratory [0/100] | NA | NA | NA |
| *Megaloceros giganteus* | Migratory [100/100] | **Yes** | Serially sampled strontium stable isotopes from tooth enamel | [14] |
| *Megalotragus priscus* | Migratory [100/100] | NA | NA | NA |
| *Megalotragus* sp. | Migratory [100/100] | **Yes** | Serially sampled strontium stable isotopes from tooth enamel | [1] |
| *Metridiochoerus* sp. | Migratory [78/100] | Unlikely | Serially sampled carbon and oxygen stable isotopes from tooth enamel | [15] |
| *Mylohyus fossilis* | Non-migratory [0/100] | NA | NA | NA |
| *Myotragus balearicus* | Migratory [69/100] | NA | NA | NA |
| *Oreamnos harringtoni* | Migratory [100/100] | NA | NA | NA |
| *Palaeolama mirifica* | Migratory [100/100] | NA | NA | NA |
| *Platygonus compressus* | Non-migratory [0/100] | Possible | Demography reconstructions from fossil remains | [16] |
| *Platygonus vetus* | Non-migratory [0/100] | NA | NA | NA |
| *Rucervus schomburgki* | Non-migratory [0/100] | NA | NA | NA |
| *Rusingoryx atopocranion* | Migratory [100/100] | **Yes** | Serially sampled strontium stable isotopes from tooth enamel | [1] |
| *Stephanorhinus hemitoechus* | Non-migratory [0/100] | NA | NA | NA |
| *Stephanorhinus kirchbergensis* | Non-migratory [0/100] | NA | NA | NA |
| *Stockoceros conklingi* | Migratory [100/100] | NA | NA | NA |
| *Syncerus antiquus* | Migratory [100/100] | Possible | Serially sampled strontium stable isotopes from tooth enamel | [1] |
|  |  | **No*** | Serially sampled strontium stable isotopes from tooth enamel | [17] |
| *Tapirus veroensis* | Migratory [100/100] | **No*** | Serially sampled strontium stable isotopes from tooth enamel | [11] |
| *Tetrameryx shuleri* | Migratory [100/100] | NA | NA | NA |

* Because migratory behavior can vary widely among populations of the same species [18], the absence of evidence for migration in a single population does not preclude its occurrence in other populations of the species.

**Table C. Model selection results for identifying the most appropriate framework for reconstructing macroevolutionary parameters including extinct taxa.** For each tree, we compared the fits of Binary State Speciation and Extinction (BiSSE), Hidden State Speciation and Extinction (HiSSE), and two-state character-independent diversification (CID-2) models using the small-sample-size-corrected Akaike Information Criterion (AIC_c_). The table reports ΔAIC_c_ values; HiSSE models were preferred for 93 of 100 tree topologies, BiSSE models were preferred for 2 trees, and CID-2 models were preferred for the remaining 5 trees.

| Tree number | HiSSE | BiSSE | CID-2 |
| --- | --- | --- | --- |
| 1 | 0.00 | 28.37 | 29.51 |
| 2 | 0.00 | 14.97 | 14.80 |
| 3 | 0.00 | 22.84 | 10.78 |
| 4 | 0.00 | 18.66 | 23.17 |
| 5 | 0.00 | 2.12 | 1.06 |
| 6 | 0.00 | 17.40 | 24.97 |
| 7 | 0.00 | 22.80 | 24.08 |
| 8 | 0.00 | 16.20 | 22.88 |
| 9 | 6.95 | 16.16 | 0.00 |
| 10 | 0.00 | 28.75 | 39.68 |
| 11 | 0.00 | 29.72 | 33.97 |
| 12 | 0.00 | 21.01 | 12.77 |
| 13 | 0.00 | 15.63 | 24.39 |
| 14 | 0.00 | 15.65 | 16.72 |
| 15 | 0.00 | 20.68 | 29.02 |
| 16 | 0.00 | 16.31 | 24.95 |
| 17 | 0.00 | 16.56 | 29.95 |
| 18 | 0.00 | 17.23 | 22.72 |
| 19 | 0.00 | 14.19 | 26.08 |
| 20 | 0.00 | 18.09 | 26.99 |
| 21 | 0.00 | 15.99 | 32.13 |
| 22 | 0.00 | 15.16 | 4.23 |
| 23 | 0.00 | 15.42 | 4.16 |
| 24 | 0.00 | 11.83 | 16.37 |
| 25 | 0.00 | 10.47 | 19.70 |
| 26 | 0.00 | 18.44 | 7.46 |
| 27 | 0.00 | 13.51 | 11.31 |
| 28 | 1.68 | 5.69 | 0.00 |
| 29 | 0.00 | 19.55 | 28.59 |
| 30 | 0.00 | 19.98 | 36.78 |
| 31 | 0.00 | 21.50 | 25.98 |
| 32 | 0.00 | 8.83 | 20.00 |
| 33 | 0.00 | 21.59 | 28.44 |
| 34 | 0.59 | 0.00 | 7.91 |
| 35 | 0.00 | 18.38 | 30.64 |
| 36 | 0.00 | 14.73 | 18.41 |
| 37 | 0.00 | 9.77 | 20.84 |
| 38 | 0.00 | 10.84 | 13.00 |
| 39 | 0.00 | 21.21 | 5.32 |
| 40 | 0.00 | 25.84 | 35.20 |
| 41 | 0.00 | 19.28 | 16.41 |
| 42 | 0.00 | 21.87 | 29.57 |
| 43 | 0.00 | 21.39 | 12.70 |
| 44 | 0.00 | 14.18 | 26.59 |
| 45 | 0.02 | 12.92 | 0.00 |
| 46 | 0.54 | 8.81 | 0.00 |
| 47 | 0.00 | 3.40 | 10.46 |
| 48 | 0.00 | 23.90 | 32.70 |
| 49 | 0.00 | 33.83 | 61.91 |
| 50 | 2.21 | 10.29 | 0.00 |
| 51 | 0.00 | 13.80 | 25.56 |
| 52 | 0.00 | 38.06 | 31.54 |
| 53 | 0.00 | 15.86 | 27.25 |
| 54 | 0.00 | 35.46 | 40.93 |
| 55 | 0.00 | 28.44 | 36.96 |
| 56 | 0.00 | 21.22 | 27.53 |
| 57 | 0.00 | 19.02 | 10.97 |
| 58 | 0.00 | 24.28 | 34.39 |
| 59 | 0.00 | 20.38 | 24.97 |
| 60 | 0.00 | 10.06 | 16.85 |
| 61 | 0.00 | 25.74 | 26.16 |
| 62 | 0.00 | 17.85 | 25.34 |
| 63 | 0.00 | 19.11 | 11.55 |
| 64 | 0.00 | 21.35 | 27.82 |
| 65 | 0.00 | 6.71 | 10.08 |
| 66 | 0.00 | 7.85 | 17.55 |
| 67 | 0.00 | 35.94 | 38.74 |
| 68 | 0.00 | 17.91 | 21.70 |
| 69 | 0.00 | 23.44 | 17.21 |
| 70 | 0.00 | 18.05 | 25.93 |
| 71 | 0.00 | 34.80 | 55.23 |
| 72 | 0.00 | 35.45 | 39.11 |
| 73 | 0.00 | 16.60 | 27.88 |
| 74 | 0.00 | 17.82 | 29.90 |
| 75 | 1.89 | 0.00 | 33.69 |
| 76 | 0.00 | 39.07 | 33.92 |
| 77 | 0.00 | 9.10 | 18.77 |
| 78 | 0.00 | 16.63 | 31.09 |
| 79 | 0.00 | 17.24 | 27.44 |
| 80 | 0.00 | 17.71 | 8.95 |
| 81 | 0.00 | 15.61 | 26.97 |
| 82 | 0.00 | 9.02 | 13.28 |
| 83 | 0.00 | 12.81 | 18.92 |
| 84 | 0.00 | 16.40 | 25.48 |
| 85 | 0.00 | 29.39 | 36.90 |
| 86 | 0.00 | 0.04 | 1.72 |
| 87 | 0.00 | 15.97 | 24.61 |
| 88 | 0.00 | 16.36 | 32.63 |
| 89 | 0.00 | 16.24 | 4.90 |
| 90 | 0.00 | 19.92 | 26.56 |
| 91 | 0.00 | 14.75 | 19.30 |
| 92 | 0.00 | 13.68 | 19.07 |
| 93 | 0.00 | 11.90 | 8.10 |
| 94 | 0.00 | 23.43 | 8.06 |
| 95 | 0.00 | 14.46 | 6.57 |
| 96 | 0.00 | 1.55 | 10.33 |
| 97 | 0.00 | 19.35 | 22.00 |
| 98 | 0.00 | 13.59 | 16.07 |
| 99 | 0.00 | 18.89 | 27.66 |
| 100 | 0.00 | 15.85 | 9.02 |
| **Median**  **[Range]** | **0.00**  **[0.00, 6.95]** | **16.93**  **[0.00, 39.07]** | **23.62**  **[0.00, 61.91]** |

| **Table D. Model selection results for identifying the best predictor of macroevolutionary parameter variation including extinct taxa.** For each tree, we compared small-sample-size-corrected Akaike Information Criterion (AIC_c_) values from logistic phylogenetic generalized linear models (phyloGLMs) with migration as a predictor to those from phylogenetic linear models (phyloLMs) with body size, grass dependence, or latitude as predictors. Analyses were conducted for all five model parameter classes (speciation, extinction, lineage turnover, net diversification, and extinction fraction). The table reports ΔAIC_c_ values; migration best predicted parameter variation relative to other traits for nearly all trees (100/100 trees for extinction rate and turnover rate, 98/100 trees for speciation rate, 99/100 trees for diversification rate, and 95/100 trees for extinction fraction). In the 8 other cases, grass dependence best predicted parameter variation. | | | | | | | | | | | | | | | | | | | | |
| --- | --- | --- | --- | --- | --- | --- | --- | --- | --- | --- | --- | --- | --- | --- | --- | --- | --- | --- | --- | --- |
|  | Extinction | | | | Speciation | | | | Lineage turnover | | | | Net diversification | | | | Extinction fraction | | | |
| Tree number | Migr-ation | Body mass | Grass | Lati-tude | Migr-ation | Body mass | Grass | Lati-tude | Migr-ation | Body mass | Grass | Lati-tude | Migr-ation | Body mass | Grass | Lati-tude | Migr-ation | Body mass | Grass | Lati-tude |
| 1 | 0.0 | 399.0 | 288.0 | 2335.3 | 0.0 | 435.1 | 305.6 | 2366.5 | 0.0 | 416.2 | 295.9 | 2350.0 | 0.0 | 155.5 | 493.1 | 2309.7 | 0.0 | 170.9 | 412.2 | 2261.4 |
| 2 | 0.0 | 324.3 | 130.2 | 2256.5 | 0.0 | 425.6 | 210.5 | 2358.1 | 0.0 | 374.7 | 165.3 | 2307.2 | 0.0 | 500.2 | 276.3 | 2433.1 | 0.0 | 287.5 | 69.8 | 2221.4 |
| 3 | 0.0 | 370.8 | 482.7 | 2363.2 | 0.0 | 375.8 | 509.8 | 2398.2 | 0.0 | 369.5 | 492.7 | 2377.8 | 0.0 | 248.8 | 476.6 | 2359.3 | 0.0 | 237.1 | 467.3 | 2347.4 |
| 4 | 0.0 | 314.0 | 487.4 | 2393.5 | 0.0 | 318.8 | 492.1 | 2398.7 | 0.0 | 315.1 | 488.4 | 2394.8 | 0.0 | 350.0 | 521.2 | 2429.2 | 0.0 | 91.5 | 235.2 | 2138.0 |
| 5 | 0.0 | 457.0 | 220.7 | 2354.1 | 0.0 | 443.3 | 209.1 | 2340.2 | 0.0 | 450.3 | 214.9 | 2347.3 | 0.0 | 328.4 | 112.8 | 2227.2 | 0.0 | 397.7 | 341.7 | 2297.0 |
| 6 | 0.0 | 405.6 | 291.1 | 2358.8 | 0.0 | 454.1 | 312.6 | 2400.4 | 0.0 | 429.0 | 300.7 | 2378.6 | 0.0 | 308.5 | 394.3 | 2343.8 | 0.0 | 168.8 | 419.1 | 2291.0 |
| 7 | 0.0 | 324.7 | 431.4 | 2252.0 | 0.0 | 339.5 | 477.2 | 2311.9 | 0.0 | 335.0 | 456.7 | 2285.1 | 0.0 | 222.4 | 442.0 | 2288.0 | 0.0 | 202.4 | 421.5 | 2266.4 |
| 8 | 0.0 | 312.5 | 475.1 | 2330.3 | 0.0 | 316.3 | 488.2 | 2351.3 | 0.0 | 308.5 | 477.5 | 2337.8 | 0.0 | 348.2 | 523.6 | 2394.7 | 0.0 | 88.1 | 229.7 | 2097.4 |
| 9 | 0.0 | 365.1 | 361.2 | 2305.4 | 0.0 | 396.8 | 394.9 | 2356.2 | 0.0 | 382.0 | 378.7 | 2331.8 | 0.0 | 265.5 | 418.1 | 2315.9 | 0.0 | 185.6 | 420.3 | 2278.7 |
| 10 | 0.0 | 413.8 | 253.6 | 2354.1 | 0.0 | 470.6 | 283.5 | 2408.4 | 0.0 | 442.6 | 265.9 | 2381.3 | 0.0 | 474.3 | 248.5 | 2408.7 | 0.0 | 466.5 | 239.8 | 2400.8 |
| 11 | 0.0 | 444.2 | 232.2 | 2382.5 | 0.0 | 455.3 | 239.5 | 2393.8 | 0.0 | 450.7 | 236.4 | 2389.1 | 0.0 | 481.2 | 259.8 | 2420.5 | 26.1 | 201.3 | 0.0 | 2143.2 |
| 12 | 0.0 | 320.5 | 408.0 | 2297.7 | 0.0 | 335.8 | 438.4 | 2340.3 | 0.0 | 336.1 | 430.8 | 2327.2 | 0.0 | 232.4 | 405.6 | 2316.8 | 0.0 | 187.9 | 361.4 | 2270.7 |
| 13 | 0.0 | 152.1 | 112.3 | 2089.9 | 0.0 | 270.3 | 300.5 | 2234.0 | 0.0 | 101.9 | 73.4 | 2043.5 | 0.0 | 192.3 | 135.8 | 2124.5 | 0.0 | 233.4 | 171.1 | 2163.7 |
| 14 | 0.0 | 438.0 | 292.9 | 2383.6 | 0.0 | 475.7 | 303.2 | 2414.9 | 0.0 | 454.6 | 295.2 | 2396.8 | 0.0 | 347.3 | 387.4 | 2359.9 | 0.0 | 272.7 | 424.8 | 2336.7 |
| 15 | 0.0 | 401.3 | 165.5 | 2304.0 | 0.0 | 456.5 | 213.0 | 2359.5 | 0.0 | 425.8 | 185.0 | 2328.7 | 0.0 | 508.6 | 258.7 | 2412.2 | 6.6 | 211.3 | 0.0 | 2118.5 |
| 16 | 0.0 | 470.5 | 254.0 | 2362.8 | 0.0 | 200.5 | 16.8 | 2096.0 | 0.0 | 466.6 | 252.3 | 2358.6 | 0.0 | 475.6 | 258.8 | 2368.3 | 0.0 | 470.8 | 254.1 | 2363.4 |
| 17 | 0.0 | 382.4 | 258.6 | 2312.0 | 0.0 | 444.1 | 284.9 | 2365.4 | 0.0 | 413.2 | 270.5 | 2338.3 | 0.0 | 415.6 | 229.9 | 2334.0 | 0.0 | 396.5 | 252.4 | 2324.6 |
| 18 | 0.0 | 450.1 | 200.8 | 2351.4 | 0.0 | 458.4 | 207.5 | 2359.8 | 0.0 | 454.8 | 204.5 | 2356.1 | 0.0 | 482.5 | 228.4 | 2384.3 | 0.0 | 346.8 | 92.4 | 2249.0 |
| 19 | 0.0 | 367.8 | 274.6 | 2327.9 | 0.0 | 425.4 | 304.3 | 2377.8 | 0.0 | 395.5 | 288.1 | 2351.7 | 0.0 | 312.3 | 417.4 | 2347.3 | 0.0 | 264.6 | 442.6 | 2336.3 |
| 20 | 0.0 | 358.0 | 459.7 | 2298.6 | 0.0 | 366.6 | 483.1 | 2330.4 | 0.0 | 358.3 | 467.4 | 2310.9 | 0.0 | 265.5 | 453.5 | 2306.8 | 0.0 | 221.9 | 409.6 | 2260.3 |
| 21 | 0.0 | 458.3 | 317.6 | 2399.9 | 0.0 | 488.0 | 330.5 | 2425.7 | 0.0 | 469.6 | 320.4 | 2409.2 | 0.0 | 203.9 | 392.4 | 2291.3 | 0.0 | 78.3 | 272.7 | 2155.8 |
| 22 | 0.0 | 365.1 | 244.8 | 2283.6 | 0.0 | 426.7 | 282.1 | 2338.6 | 0.0 | 392.0 | 259.3 | 2307.0 | 0.0 | 252.7 | 409.3 | 2288.0 | 0.0 | 164.5 | 456.5 | 2280.8 |
| 23 | 0.0 | 377.7 | 245.4 | 2323.4 | 0.0 | 413.7 | 261.9 | 2354.8 | 0.0 | 394.6 | 252.2 | 2337.9 | 0.0 | 345.4 | 255.5 | 2312.2 | 0.0 | 184.7 | 330.9 | 2246.8 |
| 24 | 0.0 | 456.1 | 204.4 | 2356.8 | 14.7 | 229.9 | 0.0 | 2133.4 | 0.0 | 391.6 | 171.5 | 2291.5 | 0.0 | 465.4 | 210.6 | 2366.8 | 0.0 | 458.0 | 203.8 | 2359.0 |
| 25 | 0.0 | 370.1 | 126.2 | 2270.4 | 0.0 | 376.0 | 131.1 | 2276.3 | 0.0 | 374.2 | 129.8 | 2274.6 | 0.0 | 395.6 | 146.7 | 2296.2 | 0.0 | 245.4 | 304.7 | 2151.9 |
| 26 | 0.0 | 367.9 | 507.1 | 2357.6 | 0.0 | 380.2 | 535.6 | 2392.2 | 0.0 | 370.2 | 518.3 | 2372.4 | 0.0 | 308.7 | 500.2 | 2360.1 | 0.0 | 284.9 | 476.4 | 2335.3 |
| 27 | 0.0 | 351.5 | 270.3 | 2314.7 | 0.0 | 408.0 | 303.3 | 2364.6 | 0.0 | 379.4 | 286.4 | 2339.2 | 0.0 | 180.3 | 490.5 | 2333.3 | 0.0 | 155.8 | 485.6 | 2320.2 |
| 28 | 0.0 | 428.5 | 205.6 | 2356.6 | 0.0 | 221.7 | 14.7 | 2152.8 | 0.0 | 313.2 | 183.1 | 2240.4 | 0.0 | 459.3 | 230.9 | 2388.3 | 0.0 | 453.7 | 225.9 | 2382.3 |
| 29 | 0.0 | 193.9 | 50.2 | 2140.7 | 0.0 | 179.6 | 10.7 | 2125.2 | 0.0 | 187.9 | 29.5 | 2134.0 | 78.8 | 217.3 | 0.0 | 2159.8 | 0.0 | 325.9 | 276.5 | 2266.9 |
| 30 | 0.0 | 324.8 | 438.7 | 2306.3 | 0.0 | 332.0 | 468.9 | 2346.4 | 0.0 | 325.7 | 451.5 | 2324.6 | 0.0 | 231.9 | 439.9 | 2329.6 | 0.0 | 189.6 | 397.3 | 2286.2 |
| 31 | 0.0 | 474.1 | 259.2 | 2412.2 | 0.0 | 475.2 | 256.6 | 2413.0 | 0.0 | 474.8 | 257.9 | 2412.7 | 0.0 | 423.7 | 401.1 | 2369.0 | 0.0 | 311.9 | 253.6 | 2261.4 |
| 32 | 0.0 | 362.4 | 193.8 | 2258.3 | 0.0 | 274.8 | 77.0 | 2173.0 | 0.0 | 271.9 | 207.9 | 2174.6 | 0.0 | 479.9 | 267.7 | 2376.9 | 0.0 | 523.2 | 312.5 | 2419.7 |
| 33 | 0.0 | 478.5 | 262.0 | 2411.3 | 0.0 | 479.0 | 262.1 | 2411.8 | 0.0 | 479.5 | 262.8 | 2412.3 | 0.0 | 358.0 | 138.9 | 2291.2 | 0.0 | 446.4 | 239.1 | 2378.5 |
| 34 | 0.0 | 464.5 | 393.3 | 2407.3 | 0.0 | 491.9 | 404.0 | 2429.8 | 0.0 | 479.6 | 399.7 | 2419.8 | 0.0 | 305.4 | 421.5 | 2323.9 | 0.0 | 73.7 | 434.7 | 2236.1 |
| 35 | 0.0 | 511.4 | 311.3 | 2401.0 | 0.0 | 517.9 | 314.8 | 2407.7 | 0.0 | 531.6 | 329.7 | 2421.3 | 0.0 | 510.3 | 304.0 | 2400.9 | 0.0 | 220.5 | 75.9 | 2116.5 |
| 36 | 0.0 | 415.3 | 275.6 | 2364.1 | 0.0 | 464.4 | 295.3 | 2406.3 | 0.0 | 443.4 | 288.3 | 2388.5 | 0.0 | 317.1 | 348.3 | 2327.4 | 0.0 | 188.2 | 410.1 | 2291.8 |
| 37 | 0.0 | 411.2 | 334.0 | 2341.0 | 0.0 | 420.8 | 310.5 | 2341.0 | 0.0 | 383.4 | 289.4 | 2308.2 | 0.0 | 163.6 | 453.9 | 2271.7 | 0.0 | 103.3 | 434.3 | 2237.4 |
| 38 | 0.0 | 420.5 | 227.9 | 2334.8 | 0.0 | 376.9 | 169.6 | 2292.4 | 0.0 | 375.7 | 235.3 | 2293.5 | 0.0 | 418.9 | 203.3 | 2334.1 | 0.0 | 484.8 | 275.8 | 2399.1 |
| 39 | 0.0 | 317.5 | 416.7 | 2261.5 | 0.0 | 329.2 | 458.6 | 2318.0 | 0.0 | 321.9 | 436.6 | 2290.3 | 0.0 | 229.7 | 427.3 | 2287.3 | 0.0 | 223.7 | 421.6 | 2280.3 |
| 40 | 0.0 | 309.9 | 106.3 | 2206.6 | 0.0 | 337.4 | 112.3 | 2234.4 | 0.0 | 295.1 | 75.9 | 2191.9 | 0.0 | 464.8 | 231.2 | 2362.3 | 17.4 | 187.9 | 0.0 | 2089.7 |
| 41 | 0.0 | 493.5 | 258.7 | 2417.1 | 28.9 | 215.1 | 0.0 | 2140.9 | 0.0 | 481.1 | 252.3 | 2404.2 | 0.0 | 474.7 | 239.1 | 2398.7 | 0.0 | 470.8 | 235.4 | 2394.6 |
| 42 | 0.0 | 454.6 | 248.8 | 2402.3 | 0.0 | 204.5 | 6.2 | 2154.0 | 0.0 | 469.2 | 266.3 | 2416.4 | 0.0 | 455.6 | 249.5 | 2403.6 | 0.0 | 442.7 | 236.6 | 2390.6 |
| 43 | 0.0 | 454.8 | 283.0 | 2421.6 | 0.0 | 489.4 | 295.5 | 2451.2 | 0.0 | 472.5 | 289.0 | 2436.6 | 0.0 | 388.1 | 297.4 | 2381.0 | 0.0 | 330.5 | 354.5 | 2365.1 |
| 44 | 0.0 | 494.0 | 257.0 | 2420.8 | 0.0 | 246.2 | 30.4 | 2175.1 | 0.0 | 490.0 | 254.9 | 2416.4 | 0.0 | 499.0 | 261.9 | 2426.0 | 0.0 | 497.8 | 260.7 | 2424.6 |
| 45 | 0.0 | 329.2 | 149.4 | 2243.4 | 0.0 | 410.9 | 185.9 | 2325.8 | 0.0 | 382.1 | 167.2 | 2296.7 | 0.0 | 468.7 | 232.7 | 2384.4 | 0.0 | 280.1 | 50.5 | 2197.0 |
| 46 | 0.0 | 308.9 | 326.5 | 2236.8 | 0.0 | 227.6 | 16.8 | 2154.6 | 0.0 | 209.4 | 43.7 | 2138.6 | 0.0 | 427.2 | 198.7 | 2352.7 | 0.0 | 468.9 | 243.0 | 2393.3 |
| 47 | 0.0 | 367.9 | 461.0 | 2308.0 | 0.0 | 377.5 | 506.1 | 2366.1 | 0.0 | 365.9 | 477.1 | 2332.1 | 0.0 | 241.3 | 449.4 | 2307.6 | 0.0 | 231.8 | 440.3 | 2296.7 |
| 48 | 0.0 | 373.3 | 177.4 | 2288.7 | 0.0 | 351.5 | 158.7 | 2266.9 | 0.0 | 367.6 | 173.1 | 2283.0 | 0.0 | 197.1 | 170.7 | 2117.1 | 0.0 | 362.8 | 374.3 | 2282.1 |
| 49 | 0.0 | 251.8 | 333.5 | 2184.5 | 0.0 | 245.7 | 339.4 | 2198.4 | 0.0 | 250.5 | 337.3 | 2191.9 | 0.0 | 239.2 | 414.4 | 2310.9 | 0.0 | 249.1 | 339.8 | 2191.8 |
| 50 | 0.0 | 319.0 | 238.0 | 2240.1 | 0.0 | 319.3 | 101.4 | 2240.2 | 0.0 | 295.8 | 105.5 | 2217.6 | 0.0 | 447.9 | 215.7 | 2368.6 | 0.0 | 374.5 | 146.8 | 2295.2 |
| 51 | 0.0 | 373.7 | 172.9 | 2300.5 | 0.0 | 386.1 | 184.8 | 2313.0 | 0.0 | 379.8 | 178.7 | 2306.6 | 0.0 | 455.4 | 250.9 | 2382.3 | 0.0 | 150.6 | 253.1 | 2094.0 |
| 52 | 0.0 | 422.1 | 305.2 | 2377.9 | 0.0 | 466.4 | 324.5 | 2415.8 | 0.0 | 443.0 | 313.5 | 2395.5 | 0.0 | 259.1 | 473.9 | 2362.0 | 0.0 | 172.8 | 505.9 | 2348.2 |
| 53 | 0.0 | 382.4 | 280.3 | 2354.5 | 0.0 | 428.9 | 302.9 | 2394.8 | 0.0 | 403.7 | 289.7 | 2372.6 | 0.0 | 170.2 | 487.6 | 2340.6 | 0.0 | 187.9 | 439.5 | 2314.9 |
| 54 | 0.0 | 396.7 | 279.2 | 2361.6 | 0.0 | 448.6 | 301.5 | 2406.2 | 0.0 | 421.8 | 288.8 | 2382.8 | 0.0 | 329.8 | 345.9 | 2343.7 | 0.0 | 246.2 | 387.6 | 2315.3 |
| 55 | 0.0 | 466.9 | 246.5 | 2389.6 | 0.0 | 474.9 | 253.7 | 2397.7 | 0.0 | 242.0 | 21.2 | 2164.8 | 0.0 | 508.3 | 284.0 | 2431.0 | 0.0 | 301.2 | 232.7 | 2230.9 |
| 56 | 0.0 | 292.0 | 373.3 | 2234.7 | 0.0 | 337.5 | 447.0 | 2332.4 | 0.0 | 347.7 | 438.5 | 2312.6 | 0.0 | 240.7 | 440.4 | 2337.8 | 0.0 | 232.5 | 432.4 | 2329.4 |
| 57 | 0.0 | 444.2 | 299.8 | 2358.5 | 0.0 | 486.8 | 316.9 | 2395.1 | 0.0 | 463.8 | 306.4 | 2374.9 | 0.0 | 131.2 | 448.1 | 2276.2 | 0.0 | 93.1 | 386.4 | 2212.0 |
| 58 | 0.0 | 461.8 | 242.8 | 2386.6 | 0.0 | 478.7 | 252.6 | 2403.9 | 0.0 | 506.6 | 283.1 | 2431.6 | 0.0 | 495.9 | 262.5 | 2421.9 | 41.6 | 221.7 | 0.0 | 2149.5 |
| 59 | 0.0 | 463.1 | 236.6 | 2368.2 | 0.0 | 451.3 | 224.7 | 2356.4 | 0.0 | 461.5 | 235.0 | 2366.6 | 0.0 | 384.8 | 158.4 | 2290.0 | 0.0 | 387.2 | 274.7 | 2291.4 |
| 60 | 0.0 | 341.5 | 437.9 | 2319.1 | 0.0 | 348.3 | 462.8 | 2354.2 | 0.0 | 341.9 | 447.1 | 2333.7 | 0.0 | 245.5 | 446.2 | 2352.6 | 0.0 | 220.2 | 419.8 | 2324.2 |
| 61 | 0.0 | 465.0 | 241.5 | 2378.9 | 0.0 | 463.1 | 239.2 | 2377.0 | 0.0 | 351.8 | 128.1 | 2265.7 | 0.0 | 446.5 | 221.2 | 2360.5 | 0.0 | 344.2 | 253.7 | 2261.1 |
| 62 | 0.0 | 392.9 | 368.8 | 2338.8 | 0.0 | 417.6 | 389.8 | 2374.7 | 0.0 | 402.8 | 376.7 | 2354.3 | 0.0 | 188.4 | 447.2 | 2314.4 | 0.0 | 156.7 | 403.1 | 2268.1 |
| 63 | 0.0 | 388.1 | 203.0 | 2321.0 | 0.0 | 360.3 | 182.8 | 2293.2 | 0.0 | 375.2 | 193.6 | 2308.1 | 0.0 | 278.1 | 112.4 | 2212.4 | 0.0 | 412.9 | 407.5 | 2348.8 |
| 64 | 0.0 | 387.9 | 216.6 | 2301.7 | 0.0 | 373.6 | 200.4 | 2287.6 | 0.0 | 390.0 | 217.5 | 2304.0 | 0.0 | 402.0 | 224.4 | 2316.2 | 0.0 | 349.5 | 177.6 | 2264.0 |
| 65 | 0.0 | 320.3 | 262.1 | 2283.1 | 0.0 | 459.0 | 293.2 | 2388.0 | 0.0 | 402.3 | 310.4 | 2349.9 | 0.0 | 275.0 | 348.5 | 2293.1 | 0.0 | 157.3 | 359.2 | 2249.3 |
| 66 | 0.0 | 389.6 | 481.7 | 2345.0 | 0.0 | 399.4 | 509.4 | 2379.4 | 0.0 | 391.2 | 492.1 | 2359.5 | 0.0 | 236.6 | 457.7 | 2305.0 | 0.0 | 167.4 | 393.8 | 2234.4 |
| 67 | 0.0 | 483.9 | 263.7 | 2402.6 | 0.0 | 488.1 | 266.3 | 2406.9 | 0.0 | 496.0 | 274.9 | 2414.8 | 0.0 | 479.4 | 254.3 | 2399.0 | 34.0 | 209.5 | 0.0 | 2131.8 |
| 68 | 0.0 | 384.0 | 275.9 | 2339.7 | 0.0 | 428.4 | 300.9 | 2379.0 | 0.0 | 402.5 | 284.7 | 2355.6 | 0.0 | 177.8 | 460.1 | 2312.7 | 0.0 | 159.1 | 398.2 | 2265.7 |
| 69 | 0.0 | 358.8 | 258.5 | 2319.0 | 0.0 | 407.4 | 289.4 | 2362.7 | 0.0 | 380.1 | 271.0 | 2337.8 | 0.0 | 147.1 | 463.5 | 2311.6 | 0.0 | 184.9 | 374.4 | 2268.2 |
| 70 | 0.0 | 300.4 | 399.3 | 2242.1 | 0.0 | 322.2 | 440.8 | 2292.6 | 0.0 | 305.0 | 413.9 | 2261.9 | 0.0 | 238.3 | 433.6 | 2288.3 | 0.0 | 202.9 | 398.8 | 2252.1 |
| 71 | 0.0 | 409.2 | 293.3 | 2360.5 | 0.0 | 449.4 | 312.4 | 2395.3 | 0.0 | 425.0 | 298.4 | 2373.5 | 0.0 | 208.6 | 456.7 | 2313.8 | 0.0 | 133.4 | 422.0 | 2263.2 |
| 72 | 0.0 | 236.1 | 324.1 | 2168.8 | 0.0 | 234.6 | 324.0 | 2166.0 | 0.0 | 235.3 | 324.9 | 2167.4 | 0.0 | 215.7 | 145.4 | 2140.5 | 0.0 | 394.9 | 488.5 | 2331.1 |
| 73 | 0.0 | 34.7 | 163.6 | 2044.4 | 0.0 | 468.7 | 233.9 | 2360.3 | 0.0 | 309.0 | 336.9 | 2266.6 | 0.0 | 243.3 | 138.0 | 2166.5 | 0.0 | 240.4 | 137.0 | 2164.1 |
| 74 | 0.0 | 277.4 | 134.1 | 2202.2 | 0.0 | 269.6 | 127.0 | 2194.5 | 0.0 | 273.9 | 130.9 | 2198.8 | 0.0 | 311.8 | 165.3 | 2236.1 | 0.0 | 511.1 | 348.2 | 2432.5 |
| 75 | 0.0 | 498.5 | 299.1 | 2401.3 | 0.0 | 477.6 | 273.9 | 2380.8 | 0.0 | 506.1 | 304.1 | 2409.2 | 0.0 | 492.1 | 283.6 | 2396.3 | 0.0 | 187.9 | 7.1 | 2095.5 |
| 76 | 0.0 | 491.6 | 349.6 | 2426.3 | 0.0 | 493.2 | 350.9 | 2428.0 | 0.0 | 494.2 | 352.1 | 2429.0 | 0.0 | 459.0 | 314.2 | 2394.1 | 0.0 | 184.7 | 309.4 | 2129.9 |
| 77 | 0.0 | 264.8 | 198.7 | 2244.3 | 0.0 | 394.7 | 282.7 | 2361.1 | 0.0 | 360.6 | 270.6 | 2333.0 | 0.0 | 375.8 | 342.1 | 2378.5 | 0.0 | 354.3 | 363.1 | 2372.9 |
| 78 | 0.0 | 334.7 | 254.0 | 2304.2 | 0.0 | 407.2 | 293.6 | 2367.9 | 0.0 | 419.2 | 322.0 | 2384.1 | 0.0 | 270.3 | 454.3 | 2357.1 | 0.0 | 230.1 | 476.4 | 2351.1 |
| 79 | 0.0 | 458.2 | 301.1 | 2397.0 | 0.0 | 488.5 | 315.9 | 2424.0 | 0.0 | 469.2 | 304.1 | 2406.3 | 0.0 | 298.6 | 420.6 | 2334.9 | 0.0 | 112.6 | 373.5 | 2220.8 |
| 80 | 0.0 | 456.1 | 637.1 | 2746.6 | 0.0 | 475.5 | 670.1 | 2783.0 | 0.0 | 464.7 | 652.9 | 2764.4 | 0.0 | 377.7 | 618.5 | 2730.0 | 0.0 | 344.0 | 585.8 | 2694.9 |
| 81 | 0.0 | 415.5 | 294.9 | 2362.6 | 0.0 | 455.8 | 313.0 | 2397.3 | 0.0 | 433.4 | 301.5 | 2377.6 | 0.0 | 285.6 | 418.6 | 2335.3 | 0.0 | 129.6 | 433.2 | 2277.6 |
| 82 | 0.0 | 225.1 | 229.3 | 2203.5 | 0.0 | 462.2 | 273.5 | 2378.8 | 0.0 | 371.2 | 319.2 | 2322.1 | 0.0 | 264.4 | 278.3 | 2250.9 | 0.0 | 185.0 | 285.1 | 2220.9 |
| 83 | 0.0 | 264.8 | 421.3 | 2311.3 | 0.0 | 286.5 | 451.3 | 2351.0 | 0.0 | 261.7 | 424.4 | 2321.2 | 0.0 | 329.1 | 493.7 | 2399.0 | 0.0 | 90.7 | 217.2 | 2111.9 |
| 84 | 0.0 | 439.8 | 307.6 | 2379.6 | 0.0 | 470.2 | 321.6 | 2406.0 | 0.0 | 455.6 | 314.7 | 2393.3 | 0.0 | 380.5 | 298.3 | 2334.7 | 0.0 | 235.8 | 336.2 | 2257.9 |
| 85 | 0.0 | 382.0 | 287.9 | 2336.1 | 0.0 | 406.8 | 282.5 | 2352.3 | 0.0 | 370.6 | 261.2 | 2320.2 | 0.0 | 186.5 | 465.0 | 2327.0 | 0.0 | 127.6 | 470.1 | 2309.8 |
| 86 | 0.0 | 324.9 | 235.1 | 2278.9 | 0.0 | 375.7 | 261.3 | 2322.6 | 0.0 | 325.7 | 223.6 | 2276.1 | 0.0 | 162.2 | 475.6 | 2324.1 | 0.0 | 152.7 | 457.0 | 2306.2 |
| 87 | 0.0 | 267.8 | 182.3 | 2221.8 | 0.0 | 344.7 | 240.1 | 2293.0 | 0.0 | 280.9 | 186.0 | 2232.0 | 0.0 | 137.8 | 486.2 | 2321.3 | 0.0 | 177.7 | 444.0 | 2302.6 |
| 88 | 0.0 | 418.5 | 193.6 | 2319.6 | 0.0 | 417.0 | 190.9 | 2318.3 | 0.0 | 389.6 | 164.0 | 2290.8 | 0.0 | 435.8 | 204.6 | 2337.8 | 0.0 | 178.5 | 73.4 | 2090.4 |
| 89 | 0.0 | 418.4 | 264.2 | 2346.5 | 0.0 | 488.6 | 297.5 | 2413.1 | 0.0 | 453.4 | 276.3 | 2379.2 | 0.0 | 483.5 | 245.1 | 2403.9 | 0.0 | 464.1 | 224.4 | 2384.4 |
| 90 | 0.0 | 410.8 | 305.3 | 2358.5 | 0.0 | 476.0 | 335.1 | 2415.3 | 0.0 | 442.3 | 318.6 | 2385.5 | 0.0 | 302.9 | 389.6 | 2334.0 | 0.0 | 208.1 | 449.2 | 2317.2 |
| 91 | 0.0 | 409.7 | 364.4 | 2334.9 | 0.0 | 205.7 | 32.1 | 2135.3 | 0.0 | 335.6 | 304.8 | 2273.1 | 0.0 | 405.6 | 184.0 | 2330.8 | 0.0 | 473.4 | 255.6 | 2397.6 |
| 92 | 0.0 | 465.2 | 238.8 | 2356.6 | 0.0 | 236.7 | 29.0 | 2131.1 | 0.0 | 375.6 | 182.8 | 2265.8 | 0.0 | 484.3 | 254.3 | 2376.4 | 0.0 | 472.2 | 242.3 | 2364.0 |
| 93 | 0.0 | 507.6 | 298.4 | 2428.4 | 0.0 | 499.6 | 292.5 | 2420.8 | 0.0 | 511.9 | 303.8 | 2432.9 | 0.0 | 382.4 | 198.9 | 2308.3 | 0.0 | 218.3 | 66.4 | 2150.3 |
| 94 | 0.0 | 312.2 | 125.0 | 2252.6 | 0.0 | 303.6 | 118.4 | 2244.1 | 0.0 | 311.0 | 124.4 | 2251.4 | 0.0 | 311.6 | 122.6 | 2251.9 | 0.0 | 438.4 | 243.2 | 2377.8 |
| 95 | 0.0 | 532.1 | 320.1 | 2419.0 | 0.0 | 536.2 | 294.1 | 2424.6 | 0.0 | 535.5 | 298.6 | 2423.3 | 0.0 | 493.9 | 249.3 | 2383.6 | 0.0 | 455.8 | 212.1 | 2345.7 |
| 96 | 0.0 | 258.9 | 213.9 | 2194.1 | 0.0 | 271.1 | 150.9 | 2205.5 | 0.0 | 265.3 | 175.7 | 2200.0 | 0.0 | 368.8 | 146.8 | 2303.8 | 0.0 | 198.6 | 124.0 | 2143.6 |
| 97 | 0.0 | 426.5 | 285.0 | 2356.6 | 0.0 | 459.8 | 298.8 | 2385.5 | 0.0 | 442.7 | 291.4 | 2370.6 | 0.0 | 86.0 | 407.8 | 2234.4 | 0.0 | 97.8 | 164.4 | 2097.3 |
| 98 | 0.0 | 388.8 | 480.6 | 2351.9 | 0.0 | 346.8 | 457.3 | 2342.5 | 0.0 | 335.9 | 436.4 | 2315.3 | 0.0 | 255.2 | 448.2 | 2347.2 | 0.0 | 235.4 | 428.6 | 2326.5 |
| 99 | 0.0 | 502.7 | 248.5 | 2391.9 | 0.0 | 509.7 | 254.8 | 2399.0 | 0.0 | 507.3 | 252.6 | 2396.5 | 0.0 | 521.8 | 264.6 | 2411.5 | 0.0 | 203.1 | 34.9 | 2099.0 |
| 100 | 0.0 | 224.8 | 0.3 | 2159.4 | 0.0 | 474.6 | 241.6 | 2407.5 | 0.0 | 499.2 | 267.9 | 2431.6 | 0.0 | 468.5 | 235.5 | 2401.7 | 0.0 | 442.3 | 209.4 | 2375.4 |
| **Median [Range]** | **0.0**  **[0, 0]** | **388.5**  **[34, 532]** | **267.2**  **[0.3, 637]** | **2337 [2044, 2747]** | **0.0 [0, 29]** | **412.3 [180, 536]** | **290.9 [0, 670]** | **2360.0 [2096, 2783]** | **0.0 [0, 0]** | **390.6 [102, 536]** | **287.2 [21, 653]** | **2338.1 [2044, 2764]** | **0.0 [0, 79]** | **328.8 [85, 522]** | **301.1 [0, 619]** | **2335.1 [2117, 2730]** | **0.0 [0, 42]** | **226.9 [73, 523]** | **321.7 [0, 586]** | **2279.5 [2089, 2695]** |

**Table E. Model selection results for identifying the most appropriate framework for reconstructing macroevolutionary parameters for only extant taxa.** For each tree, we compared the fits of Binary State Speciation and Extinction (BiSSE), Hidden State Speciation and Extinction (HiSSE), and two-state character-independent diversification (CID-2) models using the small-sample-size-corrected Akaike Information Criterion (AIC_c_). The table reports ΔAIC_c_ values; HiSSE models were preferred for 88 of 100 tree topologies, and CID-2 models were preferred for the remaining 12 trees.

| Tree number | HiSSE | BiSSE | CID2 |
| --- | --- | --- | --- |
| 1 | 0.00 | 12.13 | 21.22 |
| 2 | 0.00 | 10.83 | 20.23 |
| 3 | 0.00 | 16.27 | 26.72 |
| 4 | 0.00 | 15.97 | 24.05 |
| 5 | 3.02 | 16.41 | 0.00 |
| 6 | 0.00 | 10.68 | 28.32 |
| 7 | 0.00 | 16.85 | 20.35 |
| 8 | 0.00 | 16.15 | 18.85 |
| 9 | 0.00 | 17.26 | 22.08 |
| 10 | 0.00 | 13.38 | 19.51 |
| 11 | 0.00 | 10.13 | 15.04 |
| 12 | 0.00 | 16.87 | 16.97 |
| 13 | 0.00 | 17.31 | 25.08 |
| 14 | 0.00 | 16.53 | 16.83 |
| 15 | 0.00 | 19.17 | 21.28 |
| 16 | 0.00 | 12.61 | 21.06 |
| 17 | 0.00 | 14.15 | 24.06 |
| 18 | 0.00 | 14.86 | 16.98 |
| 19 | 0.00 | 14.18 | 17.49 |
| 20 | 2.17 | 14.36 | 0.00 |
| 21 | 0.00 | 15.16 | 33.89 |
| 22 | 0.00 | 14.41 | 26.72 |
| 23 | 0.00 | 13.21 | 17.83 |
| 24 | 0.00 | 10.67 | 16.62 |
| 25 | 0.00 | 13.91 | 23.31 |
| 26 | 0.00 | 14.54 | 17.36 |
| 27 | 0.00 | 10.55 | 26.13 |
| 28 | 0.00 | 16.18 | 21.44 |
| 29 | 0.00 | 18.97 | 22.30 |
| 30 | 0.00 | 12.39 | 24.53 |
| 31 | 0.00 | 17.01 | 12.75 |
| 32 | 0.00 | 13.64 | 17.92 |
| 33 | 0.00 | 18.02 | 22.76 |
| 34 | 0.00 | 14.37 | 20.70 |
| 35 | 0.00 | 13.00 | 25.45 |
| 36 | 0.00 | 15.68 | 23.64 |
| 37 | 4.70 | 17.16 | 0.00 |
| 38 | 0.00 | 16.88 | 22.16 |
| 39 | 0.00 | 16.42 | 25.49 |
| 40 | 0.00 | 15.08 | 19.19 |
| 41 | 0.00 | 16.36 | 19.64 |
| 42 | 0.40 | 17.35 | 0.00 |
| 43 | 0.00 | 12.95 | 28.12 |
| 44 | 0.00 | 12.13 | 17.68 |
| 45 | 0.00 | 15.57 | 25.43 |
| 46 | 0.00 | 9.53 | 20.51 |
| 47 | 0.00 | 16.89 | 22.51 |
| 48 | 0.00 | 19.80 | 21.93 |
| 49 | 0.00 | 13.66 | 21.15 |
| 50 | 0.00 | 13.32 | 24.77 |
| 51 | 0.00 | 13.31 | 13.66 |
| 52 | 0.00 | 17.20 | 16.23 |
| 53 | 0.00 | 14.52 | 2.96 |
| 54 | 0.00 | 12.06 | 23.33 |
| 55 | 5.12 | 16.46 | 0.00 |
| 56 | 0.00 | 17.33 | 26.74 |
| 57 | 4.48 | 17.72 | 0.00 |
| 58 | 0.00 | 23.67 | 20.71 |
| 59 | 0.00 | 17.96 | 19.89 |
| 60 | 0.00 | 6.46 | 25.05 |
| 61 | 0.00 | 17.43 | 20.39 |
| 62 | 0.00 | 13.82 | 16.72 |
| 63 | 0.00 | 18.74 | 23.55 |
| 64 | 0.00 | 20.45 | 20.98 |
| 65 | 0.00 | 13.50 | 19.74 |
| 66 | 0.00 | 10.10 | 20.36 |
| 67 | 0.00 | 17.10 | 22.10 |
| 68 | 0.00 | 12.20 | 17.63 |
| 69 | 0.00 | 14.51 | 25.35 |
| 70 | 0.00 | 14.41 | 25.88 |
| 71 | 0.00 | 14.78 | 16.56 |
| 72 | 0.00 | 17.40 | 20.34 |
| 73 | 0.44 | 14.97 | 0.00 |
| 74 | 0.47 | 12.31 | 0.00 |
| 75 | 3.56 | 10.37 | 0.00 |
| 76 | 0.00 | 18.55 | 15.93 |
| 77 | 0.00 | 8.59 | 25.12 |
| 78 | 0.00 | 15.11 | 24.14 |
| 79 | 0.00 | 15.00 | 16.67 |
| 80 | 0.00 | 13.17 | 23.18 |
| 81 | 0.00 | 15.71 | 24.73 |
| 82 | 0.00 | 15.16 | 20.97 |
| 83 | 0.00 | 16.42 | 29.13 |
| 84 | 0.00 | 13.48 | 18.04 |
| 85 | 7.39 | 18.61 | 0.00 |
| 86 | 0.00 | 9.80 | 17.40 |
| 87 | 0.00 | 16.25 | 31.39 |
| 88 | 0.00 | 15.20 | 27.12 |
| 89 | 0.00 | 20.96 | 26.43 |
| 90 | 0.00 | 14.31 | 18.62 |
| 91 | 0.00 | 17.91 | 20.91 |
| 92 | 0.00 | 12.10 | 22.15 |
| 93 | 0.00 | 14.13 | 24.47 |
| 94 | 3.22 | 20.12 | 0.00 |
| 95 | 0.00 | 13.02 | 20.74 |
| 96 | 3.18 | 17.06 | 0.00 |
| 97 | 0.00 | 16.71 | 18.21 |
| 98 | 0.00 | 12.02 | 20.24 |
| 99 | 0.00 | 10.37 | 17.24 |
| 100 | 0.00 | 13.26 | 23.59 |
| **Median**  **[Range]** | **0.38**  **[0.00, 7.39]** | **14.93**  **[6.46, 23.67]** | **18.83**  **[0.00, 33.89]** |

| **Table F. Model selection results for identifying the best predictor of macroevolutionary parameter variation for only extant taxa.** For each tree, we compared small-sample-size-corrected Akaike Information Criterion (AIC_c_) values from logistic phylogenetic generalized linear models (phyloGLMs) with migration as a predictor to those from phylogenetic linear models (phyloLMs) with body size, grass dependence, or latitude as predictors. Analyses were conducted for all five model parameters (speciation, extinction, lineage turnover, net diversification, and extinction fraction). The table reports ΔAIC_c_ values; migration best predicted parameter variation relative to other traits for the majority of trees (68/100 trees for extinction rate, 52/100 trees for speciation rate, 50/100 trees for turnover rate, 96/100 trees for diversification rate, and 66/100 trees for extinction fraction). In other cases, body mass generally best predicted parameter variation, except in two cases in which grass dependence best predicted variation. | | | | | | | | | | | | | | | | | | | | |
| --- | --- | --- | --- | --- | --- | --- | --- | --- | --- | --- | --- | --- | --- | --- | --- | --- | --- | --- | --- | --- |
|  | Extinction | | | | Speciation | | | | Lineage turnover | | | | Net diversification | | | | Extinction fraction | | | |
| Tree number | Migr-ation | Body mass | Grass | Lati-tude | Migr-ation | Body mass | Grass | Lati-tude | Migr-ation | Body mass | Grass | Lati-tude | Migr-ation | Body mass | Grass | Lati-tude | Migr-ation | Body mass | Grass | Lati-tude |
| 1 | 0.0 | 102.8 | 133.7 | 1742.1 | 45.4 | 0.0 | 28.3 | 1636.5 | 44.7 | 0.0 | 48.7 | 1640.3 | 0.0 | 95.8 | 123.2 | 1734.1 | 0.0 | 94.3 | 123.4 | 1732.5 |
| 2 | 0.0 | 103.7 | 145.3 | 1756.3 | 24.6 | 0.0 | 42.2 | 1649.6 | 73.5 | 0.0 | 72.1 | 1650.5 | 0.0 | 85.7 | 124.9 | 1737.4 | 0.0 | 107.1 | 148.5 | 1759.8 |
| 3 | 0.0 | 117.8 | 159.6 | 1749.6 | 0.0 | 13.0 | 53.1 | 1643.1 | 80.1 | 0.0 | 80.0 | 1621.5 | 0.0 | 92.7 | 132.0 | 1724.1 | 0.0 | 124.0 | 163.1 | 1756.1 |
| 4 | 0.0 | 40.6 | 129.4 | 1659.9 | 0.0 | 54.2 | 115.5 | 1672.7 | 56.2 | 0.0 | 96.9 | 1614.4 | 0.0 | 73.6 | 151.7 | 1692.8 | 0.0 | 61.9 | 146.0 | 1681.1 |
| 5 | 117.7 | 0.0 | 52.7 | 1614.0 | 0.0 | 88.4 | 124.2 | 1719.2 | 0.0 | 106.2 | 133.4 | 1737.0 | 10.8 | 0.0 | 44.4 | 1629.2 | 123.6 | 0.0 | 55.6 | 1614.8 |
| 6 | 0.0 | 97.1 | 135.3 | 1752.2 | 5.4 | 0.0 | 51.8 | 1652.7 | 84.0 | 0.0 | 39.8 | 1648.1 | 0.0 | 74.4 | 118.7 | 1728.7 | 0.0 | 107.2 | 130.5 | 1761.8 |
| 7 | 0.0 | 5.9 | 43.5 | 1633.5 | 0.0 | 79.8 | 95.9 | 1710.7 | 114.5 | 0.0 | 34.5 | 1608.7 | 0.0 | 44.4 | 72.3 | 1674.9 | 0.0 | 13.1 | 46.4 | 1642.2 |
| 8 | 90.1 | 0.0 | 42.5 | 1602.3 | 0.0 | 89.7 | 119.0 | 1702.4 | 0.0 | 124.9 | 148.2 | 1738.3 | 0.0 | 2.3 | 38.4 | 1614.0 | 97.7 | 0.0 | 58.4 | 1604.1 |
| 9 | 42.3 | 21.6 | 0.0 | 1658.9 | 0.0 | 23.2 | 70.8 | 1666.6 | 114.0 | 2.3 | 0.0 | 1637.9 | 0.0 | 108.8 | 96.9 | 1750.9 | 0.0 | 82.7 | 63.1 | 1722.8 |
| 10 | 0.0 | 95.0 | 103.6 | 1724.0 | 73.1 | 0.0 | 18.5 | 1623.6 | 1.2 | 0.0 | 26.2 | 1628.9 | 0.0 | 104.8 | 110.1 | 1732.8 | 0.0 | 119.0 | 128.6 | 1748.7 |
| 11 | 89.3 | 0.0 | 78.4 | 1630.2 | 0.0 | 105.4 | 150.4 | 1743.6 | 0.0 | 94.5 | 124.0 | 1738.9 | 0.0 | 12.5 | 68.7 | 1647.2 | 90.0 | 0.0 | 65.5 | 1631.8 |
| 12 | 0.0 | 75.7 | 102.1 | 1737.6 | 0.0 | 74.1 | 94.4 | 1734.9 | 104.3 | 0.0 | 33.4 | 1657.2 | 0.0 | 110.3 | 131.7 | 1771.6 | 0.0 | 109.1 | 135.5 | 1770.9 |
| 13 | 22.6 | 0.0 | 83.4 | 1611.2 | 0.0 | 88.8 | 139.9 | 1703.6 | 0.0 | 121.8 | 143.9 | 1735.2 | 0.0 | 47.2 | 110.5 | 1661.7 | 31.4 | 0.0 | 65.3 | 1612.6 |
| 14 | 11.8 | 0.0 | 50.3 | 1630.1 | 0.0 | 59.7 | 101.1 | 1691.2 | 0.0 | 105.7 | 141.1 | 1737.7 | 0.0 | 36.5 | 80.5 | 1667.6 | 55.2 | 0.0 | 41.3 | 1630.3 |
| 15 | 0.0 | 88.0 | 120.2 | 1711.3 | 48.8 | 0.0 | 30.7 | 1619.4 | 55.5 | 0.0 | 55.1 | 1618.9 | 0.0 | 91.9 | 120.3 | 1714.7 | 0.0 | 98.8 | 129.2 | 1721.3 |
| 16 | 0.0 | 91.5 | 149.7 | 1728.6 | 15.4 | 0.0 | 59.9 | 1635.4 | 135.2 | 0.0 | 94.6 | 1624.9 | 0.0 | 60.7 | 118.6 | 1697.3 | 0.0 | 101.5 | 153.4 | 1739.0 |
| 17 | 0.0 | 92.4 | 126.6 | 1728.3 | 69.8 | 0.0 | 39.6 | 1632.2 | 0.0 | 0.8 | 45.1 | 1637.4 | 0.0 | 117.7 | 149.5 | 1752.8 | 0.0 | 107.8 | 140.3 | 1742.7 |
| 18 | 0.0 | 92.5 | 104.6 | 1726.4 | 58.0 | 0.0 | 13.3 | 1629.9 | 11.9 | 0.0 | 28.1 | 1634.7 | 0.0 | 112.3 | 120.8 | 1745.2 | 0.0 | 121.2 | 134.0 | 1755.8 |
| 19 | 41.0 | 0.0 | 90.8 | 1625.1 | 0.0 | 78.9 | 146.3 | 1708.4 | 0.0 | 109.2 | 158.7 | 1738.9 | 0.0 | 37.2 | 113.0 | 1665.8 | 64.8 | 0.0 | 83.1 | 1626.9 |
| 20 | 95.3 | 0.0 | 71.7 | 1627.2 | 0.0 | 98.9 | 127.3 | 1735.3 | 0.0 | 114.4 | 127.8 | 1749.8 | 0.0 | 14.0 | 55.0 | 1649.6 | 100.4 | 0.0 | 53.9 | 1631.3 |
| 21 | 0.0 | 112.8 | 134.7 | 1748.9 | 27.0 | 0.0 | 20.5 | 1633.7 | 23.7 | 0.0 | 30.3 | 1632.9 | 0.0 | 92.4 | 112.5 | 1728.5 | 0.0 | 120.7 | 141.5 | 1757.7 |
| 22 | 81.8 | 0.0 | 35.1 | 1606.8 | 0.0 | 105.0 | 150.4 | 1724.4 | 0.0 | 112.8 | 149.2 | 1731.5 | 0.0 | 4.7 | 59.9 | 1622.1 | 90.5 | 0.0 | 36.5 | 1606.9 |
| 23 | 69.7 | 0.0 | 50.6 | 1594.5 | 0.0 | 113.1 | 132.3 | 1719.2 | 0.0 | 111.8 | 116.9 | 1716.2 | 0.0 | 33.1 | 63.3 | 1637.7 | 70.2 | 0.0 | 39.3 | 1599.5 |
| 24 | 85.5 | 0.0 | 52.3 | 1612.6 | 0.0 | 105.2 | 129.6 | 1730.7 | 0.0 | 108.7 | 121.3 | 1729.4 | 0.0 | 14.4 | 52.5 | 1637.3 | 79.9 | 0.0 | 47.2 | 1618.3 |
| 25 | 0.0 | 110.6 | 95.0 | 1722.9 | 113.7 | 0.0 | 21.4 | 1609.3 | 41.1 | 1.1 | 0.0 | 1611.1 | 0.0 | 50.1 | 67.5 | 1665.2 | 0.0 | 131.2 | 133.8 | 1749.2 |
| 26 | 0.0 | 114.1 | 134.6 | 1738.3 | 0.0 | 45.3 | 63.2 | 1668.9 | 105.8 | 0.0 | 42.3 | 1617.7 | 0.0 | 94.8 | 113.1 | 1718.8 | 0.0 | 119.7 | 138.6 | 1744.5 |
| 27 | 0.0 | 113.8 | 150.6 | 1757.5 | 31.1 | 0.0 | 36.3 | 1642.4 | 58.6 | 0.0 | 75.6 | 1643.0 | 0.0 | 99.9 | 132.8 | 1743.2 | 0.0 | 100.5 | 134.9 | 1743.4 |
| 28 | 57.6 | 0.0 | 54.6 | 1611.8 | 0.0 | 90.9 | 117.4 | 1711.9 | 0.0 | 111.8 | 121.5 | 1729.8 | 0.0 | 21.0 | 59.0 | 1640.8 | 69.4 | 0.0 | 47.8 | 1615.2 |
| 29 | 63.6 | 0.0 | 75.7 | 1622.6 | 0.0 | 63.3 | 104.7 | 1693.7 | 0.0 | 114.3 | 135.8 | 1742.5 | 0.0 | 15.9 | 69.0 | 1645.7 | 87.4 | 0.0 | 65.1 | 1624.5 |
| 30 | 0.0 | 107.3 | 133.1 | 1736.1 | 26.7 | 0.0 | 65.9 | 1630.1 | 109.0 | 0.0 | 48.8 | 1624.9 | 0.0 | 45.6 | 93.7 | 1684.1 | 0.0 | 114.0 | 147.6 | 1751.6 |
| 31 | 0.0 | 25.3 | 85.5 | 1666.7 | 0.0 | 88.0 | 120.8 | 1730.2 | 0.0 | 115.1 | 139.0 | 1743.6 | 0.0 | 51.8 | 95.7 | 1694.4 | 4.2 | 0.0 | 49.1 | 1641.8 |
| 32 | 78.6 | 0.0 | 119.6 | 1643.7 | 0.0 | 115.5 | 180.7 | 1772.7 | 0.0 | 128.2 | 176.7 | 1780.6 | 0.0 | 31.7 | 113.9 | 1689.2 | 81.9 | 0.0 | 108.0 | 1650.2 |
| 33 | 0.0 | 75.9 | 84.8 | 1708.5 | 104.0 | 0.0 | 25.6 | 1626.5 | 0.0 | 4.6 | 22.2 | 1636.6 | 0.0 | 104.6 | 110.2 | 1737.0 | 0.0 | 107.7 | 116.5 | 1740.3 |
| 34 | 50.3 | 0.0 | 102.9 | 1619.3 | 0.0 | 98.4 | 147.3 | 1722.1 | 0.0 | 105.9 | 120.8 | 1724.7 | 0.0 | 42.7 | 123.4 | 1666.7 | 45.6 | 0.0 | 103.0 | 1620.2 |
| 35 | 0.0 | 119.9 | 144.8 | 1726.5 | 0.0 | 26.5 | 49.3 | 1632.3 | 89.0 | 0.0 | 35.3 | 1600.0 | 0.0 | 90.5 | 113.1 | 1697.0 | 0.0 | 97.1 | 121.9 | 1703.8 |
| 36 | 95.0 | 0.0 | 70.6 | 1613.5 | 0.0 | 97.0 | 127.0 | 1722.9 | 0.0 | 116.2 | 125.0 | 1737.5 | 10.5 | 0.0 | 52.2 | 1624.5 | 101.7 | 0.0 | 68.0 | 1617.5 |
| 37 | 60.4 | 0.0 | 60.2 | 1623.5 | 0.0 | 54.2 | 83.2 | 1682.3 | 0.0 | 106.1 | 119.0 | 1733.6 | 0.0 | 15.8 | 54.2 | 1643.5 | 88.7 | 0.0 | 37.4 | 1624.2 |
| 38 | 0.0 | 6.3 | 68.0 | 1636.1 | 0.0 | 50.2 | 100.7 | 1680.9 | 0.0 | 96.0 | 137.4 | 1725.9 | 0.0 | 31.2 | 85.3 | 1662.0 | 31.2 | 0.0 | 54.1 | 1628.9 |
| 39 | 0.0 | 13.4 | 51.0 | 1649.3 | 0.0 | 59.7 | 74.3 | 1697.3 | 0.0 | 128.0 | 114.5 | 1757.8 | 0.0 | 34.3 | 59.0 | 1671.9 | 24.7 | 0.0 | 26.1 | 1636.4 |
| 40 | 0.0 | 86.2 | 81.2 | 1697.3 | 66.6 | 0.0 | 2.3 | 1607.5 | 25.7 | 0.0 | 5.3 | 1609.6 | 0.0 | 105.1 | 99.3 | 1715.7 | 0.0 | 106.2 | 100.9 | 1717.0 |
| 41 | 0.0 | 108.4 | 141.2 | 1740.5 | 0.0 | 34.3 | 70.2 | 1665.1 | 100.9 | 0.0 | 65.1 | 1623.5 | 0.0 | 88.7 | 122.3 | 1720.3 | 0.0 | 119.6 | 147.7 | 1752.2 |
| 42 | 0.0 | 80.5 | 135.8 | 1745.9 | 58.0 | 0.0 | 53.2 | 1661.0 | 36.9 | 0.0 | 66.0 | 1665.5 | 0.0 | 105.6 | 158.2 | 1769.8 | 0.0 | 108.0 | 163.2 | 1773.3 |
| 43 | 0.0 | 66.4 | 117.5 | 1700.3 | 3.9 | 0.0 | 47.7 | 1632.5 | 134.1 | 0.0 | 36.1 | 1624.2 | 0.0 | 35.2 | 84.5 | 1668.3 | 0.0 | 15.9 | 59.5 | 1648.7 |
| 44 | 0.0 | 101.5 | 152.8 | 1729.9 | 0.0 | 19.6 | 74.6 | 1647.5 | 111.5 | 0.0 | 70.3 | 1621.5 | 0.0 | 69.8 | 122.9 | 1698.1 | 0.0 | 104.8 | 147.0 | 1733.0 |
| 45 | 0.0 | 108.6 | 127.2 | 1745.2 | 37.5 | 0.0 | 19.7 | 1636.3 | 23.0 | 0.0 | 30.6 | 1635.0 | 0.0 | 104.6 | 121.2 | 1741.4 | 0.0 | 95.6 | 112.6 | 1731.3 |
| 46 | 0.0 | 110.3 | 165.3 | 1737.7 | 0.0 | 11.8 | 66.0 | 1637.6 | 106.4 | 0.0 | 60.4 | 1621.6 | 0.0 | 67.0 | 121.1 | 1693.6 | 0.0 | 107.8 | 157.9 | 1736.3 |
| 47 | 0.0 | 110.5 | 121.1 | 1714.5 | 15.4 | 0.0 | 6.7 | 1599.8 | 55.2 | 0.0 | 36.7 | 1604.7 | 0.0 | 102.8 | 109.1 | 1705.3 | 0.0 | 116.1 | 125.3 | 1719.6 |
| 48 | 0.0 | 70.7 | 102.2 | 1729.8 | 53.3 | 0.0 | 91.4 | 1661.5 | 96.3 | 0.0 | 57.7 | 1654.1 | 0.0 | 3.3 | 113.8 | 1678.1 | 127.6 | 0.0 | 87.3 | 1657.5 |
| 49 | 0.0 | 98.3 | 109.8 | 1710.0 | 57.4 | 0.0 | 11.6 | 1608.5 | 0.0 | 0.3 | 20.4 | 1611.0 | 0.0 | 118.4 | 127.7 | 1729.7 | 0.0 | 130.0 | 142.4 | 1742.6 |
| 50 | 0.0 | 101.6 | 118.6 | 1738.7 | 22.8 | 0.0 | 14.7 | 1634.8 | 60.9 | 0.0 | 26.7 | 1634.3 | 0.0 | 82.9 | 98.3 | 1719.5 | 0.0 | 115.6 | 130.4 | 1753.7 |
| 51 | 44.1 | 0.0 | 92.1 | 1604.2 | 0.0 | 99.8 | 131.5 | 1709.9 | 0.0 | 95.5 | 80.4 | 1697.9 | 0.0 | 38.8 | 118.7 | 1650.1 | 41.1 | 0.0 | 97.5 | 1606.0 |
| 52 | 0.0 | 102.4 | 132.3 | 1743.6 | 70.8 | 0.0 | 34.8 | 1638.2 | 21.0 | 0.0 | 40.9 | 1640.9 | 0.0 | 95.0 | 122.7 | 1735.8 | 0.0 | 101.9 | 131.8 | 1743.1 |
| 53 | 0.0 | 5.3 | 90.8 | 1675.1 | 0.0 | 72.7 | 122.4 | 1743.2 | 0.0 | 73.7 | 95.2 | 1736.4 | 0.0 | 60.3 | 127.9 | 1731.1 | 0.0 | 24.4 | 105.4 | 1694.3 |
| 54 | 65.5 | 0.0 | 89.2 | 1673.3 | 0.0 | 67.2 | 131.9 | 1745.9 | 0.0 | 118.5 | 172.7 | 1796.5 | 0.0 | 22.2 | 94.2 | 1700.5 | 86.1 | 0.0 | 81.5 | 1674.0 |
| 55 | 0.0 | 108.5 | 154.4 | 1727.7 | 0.0 | 20.5 | 65.3 | 1638.0 | 104.5 | 0.0 | 70.1 | 1601.8 | 0.0 | 84.9 | 129.5 | 1703.6 | 0.0 | 111.5 | 154.9 | 1731.9 |
| 56 | 0.0 | 79.2 | 104.2 | 1721.2 | 55.7 | 0.0 | 23.7 | 1638.3 | 21.9 | 0.0 | 35.5 | 1642.3 | 0.0 | 103.5 | 125.7 | 1744.7 | 0.0 | 106.5 | 131.2 | 1748.3 |
| 57 | 0.0 | 97.9 | 123.6 | 1738.3 | 0.0 | 29.9 | 58.7 | 1667.9 | 112.9 | 0.0 | 44.3 | 1624.7 | 0.0 | 68.2 | 95.3 | 1707.6 | 0.0 | 101.2 | 118.5 | 1741.2 |
| 58 | 0.0 | 80.6 | 104.2 | 1722.7 | 20.8 | 0.0 | 21.0 | 1639.5 | 74.1 | 0.0 | 50.4 | 1637.5 | 0.0 | 93.3 | 114.0 | 1734.6 | 0.0 | 106.8 | 129.4 | 1748.5 |
| 59 | 7.2 | 0.0 | 58.1 | 1631.4 | 0.0 | 85.9 | 127.1 | 1721.1 | 0.0 | 110.0 | 134.5 | 1741.9 | 0.0 | 64.4 | 112.4 | 1699.0 | 0.0 | 3.4 | 60.2 | 1635.2 |
| 60 | 28.9 | 0.0 | 53.1 | 1653.1 | 0.0 | 59.8 | 78.4 | 1718.6 | 12.2 | 0.0 | 1.4 | 1655.6 | 0.0 | 40.1 | 75.2 | 1697.2 | 48.1 | 0.0 | 55.3 | 1651.7 |
| 61 | 0.0 | 105.2 | 118.8 | 1733.1 | 67.9 | 0.0 | 47.0 | 1617.4 | 28.8 | 0.0 | 20.9 | 1617.0 | 0.0 | 27.0 | 64.5 | 1658.1 | 0.0 | 103.0 | 132.5 | 1737.4 |
| 62 | 55.3 | 0.0 | 51.1 | 1639.1 | 0.0 | 90.2 | 111.1 | 1733.4 | 0.0 | 114.9 | 121.9 | 1758.5 | 0.0 | 29.2 | 58.8 | 1671.5 | 70.6 | 0.0 | 38.5 | 1640.3 |
| 63 | 0.0 | 69.1 | 97.3 | 1706.4 | 83.6 | 0.0 | 32.9 | 1633.4 | 26.0 | 0.0 | 38.6 | 1636.4 | 0.0 | 95.1 | 121.0 | 1732.0 | 0.0 | 102.0 | 130.3 | 1739.4 |
| 64 | 0.0 | 91.6 | 113.0 | 1702.9 | 66.4 | 0.0 | 26.7 | 1602.2 | 0.0 | 7.7 | 44.7 | 1616.9 | 0.0 | 119.9 | 135.8 | 1730.8 | 0.0 | 111.4 | 129.9 | 1721.6 |
| 65 | 141.1 | 0.0 | 65.3 | 1612.0 | 0.0 | 60.4 | 112.1 | 1682.6 | 0.0 | 101.4 | 151.7 | 1724.6 | 0.0 | 0.6 | 53.5 | 1621.7 | 129.3 | 0.0 | 48.3 | 1614.0 |
| 66 | 18.6 | 0.0 | 152.6 | 1659.9 | 0.0 | 75.8 | 151.5 | 1733.0 | 0.0 | 96.6 | 111.0 | 1742.5 | 0.0 | 52.2 | 172.9 | 1713.2 | 5.4 | 0.0 | 151.8 | 1659.8 |
| 67 | 0.0 | 83.7 | 103.0 | 1701.2 | 91.0 | 0.0 | 29.4 | 1610.9 | 0.0 | 7.5 | 36.1 | 1623.5 | 0.0 | 112.5 | 128.2 | 1729.9 | 0.0 | 111.5 | 130.8 | 1728.9 |
| 68 | 75.2 | 0.0 | 106.8 | 1623.4 | 0.0 | 79.5 | 133.4 | 1708.1 | 0.0 | 122.3 | 131.9 | 1747.7 | 0.0 | 30.5 | 117.3 | 1660.0 | 90.8 | 0.0 | 113.5 | 1624.6 |
| 69 | 0.0 | 112.5 | 130.2 | 1738.9 | 0.0 | 24.5 | 38.7 | 1649.7 | 88.4 | 0.0 | 21.9 | 1620.5 | 0.0 | 75.1 | 89.7 | 1701.0 | 0.0 | 87.5 | 105.0 | 1714.2 |
| 70 | 0.0 | 84.4 | 103.8 | 1706.3 | 91.6 | 0.0 | 31.5 | 1617.5 | 0.0 | 4.7 | 35.0 | 1625.7 | 0.0 | 106.0 | 121.4 | 1727.7 | 0.0 | 105.0 | 124.4 | 1726.9 |
| 71 | 0.0 | 102.1 | 140.3 | 1727.0 | 34.7 | 0.0 | 38.7 | 1624.7 | 46.1 | 0.0 | 49.5 | 1623.2 | 0.0 | 90.0 | 126.9 | 1715.1 | 0.0 | 114.4 | 152.1 | 1739.7 |
| 72 | 3.6 | 0.0 | 50.6 | 1632.9 | 0.0 | 67.2 | 93.5 | 1702.2 | 0.0 | 124.8 | 136.5 | 1756.3 | 0.0 | 33.3 | 68.0 | 1668.2 | 46.2 | 0.0 | 43.5 | 1633.1 |
| 73 | 0.0 | 117.3 | 122.6 | 1724.3 | 78.4 | 0.0 | 15.2 | 1599.9 | 0.0 | 4.8 | 19.8 | 1612.1 | 0.0 | 118.3 | 121.0 | 1724.2 | 0.0 | 117.9 | 123.3 | 1724.9 |
| 74 | 0.0 | 137.1 | 183.3 | 1746.8 | 0.0 | 50.3 | 95.1 | 1658.0 | 65.5 | 0.0 | 58.9 | 1601.3 | 0.0 | 103.1 | 147.3 | 1711.9 | 0.0 | 135.8 | 181.8 | 1746.3 |
| 75 | 0.0 | 75.2 | 121.6 | 1719.9 | 101.9 | 0.0 | 61.7 | 1642.0 | 0.0 | 13.5 | 65.4 | 1658.0 | 0.0 | 110.1 | 154.9 | 1754.5 | 0.0 | 109.9 | 156.3 | 1754.6 |
| 76 | 74.8 | 0.0 | 75.4 | 1622.9 | 0.0 | 97.6 | 122.9 | 1727.0 | 0.0 | 112.5 | 120.2 | 1738.5 | 0.0 | 20.8 | 60.8 | 1650.3 | 83.6 | 0.0 | 55.2 | 1626.5 |
| 77 | 0.0 | 112.7 | 119.9 | 1766.8 | 0.0 | 6.7 | 69.6 | 1664.6 | 2.9 | 0.0 | 21.1 | 1649.5 | 0.0 | 109.0 | 120.9 | 1767.2 | 0.0 | 72.3 | 81.3 | 1729.2 |
| 78 | 0.0 | 117.7 | 129.4 | 1758.5 | 84.0 | 0.0 | 27.8 | 1638.2 | 0.0 | 31.9 | 49.2 | 1672.0 | 0.0 | 119.7 | 129.4 | 1760.6 | 0.0 | 105.6 | 116.9 | 1745.9 |
| 79 | 20.7 | 0.0 | 47.7 | 1625.7 | 0.0 | 73.1 | 111.7 | 1701.6 | 0.0 | 104.9 | 135.8 | 1735.1 | 0.0 | 35.7 | 77.3 | 1663.3 | 45.2 | 0.0 | 39.9 | 1626.7 |
| 80 | 0.0 | 114.6 | 138.0 | 1749.6 | 3.1 | 0.0 | 21.9 | 1632.0 | 83.1 | 0.0 | 67.2 | 1629.0 | 0.0 | 98.9 | 119.0 | 1732.9 | 0.0 | 117.9 | 141.4 | 1753.0 |
| 81 | 0.0 | 101.8 | 131.4 | 1730.3 | 31.5 | 0.0 | 30.3 | 1628.1 | 24.4 | 0.0 | 37.0 | 1627.1 | 0.0 | 107.4 | 135.6 | 1736.1 | 0.0 | 74.8 | 102.6 | 1702.2 |
| 82 | 61.2 | 0.0 | 79.1 | 1620.6 | 0.0 | 100.8 | 151.6 | 1727.2 | 0.0 | 134.1 | 169.7 | 1762.6 | 0.0 | 41.6 | 101.2 | 1666.6 | 69.0 | 0.0 | 62.3 | 1623.1 |
| 83 | 0.0 | 129.0 | 142.1 | 1792.7 | 54.7 | 0.0 | 16.6 | 1658.8 | 0.0 | 2.6 | 37.3 | 1665.7 | 0.0 | 122.9 | 131.3 | 1785.6 | 0.0 | 127.7 | 141.5 | 1791.7 |
| 84 | 124.3 | 0.0 | 59.8 | 1609.1 | 0.0 | 110.5 | 147.2 | 1732.3 | 0.0 | 116.3 | 144.8 | 1739.1 | 0.0 | 35.2 | 79.1 | 1655.6 | 112.8 | 0.0 | 56.8 | 1613.5 |
| 85 | 0.0 | 111.5 | 122.2 | 1731.2 | 60.0 | 0.0 | 10.7 | 1615.4 | 0.0 | 6.7 | 28.3 | 1625.6 | 0.0 | 102.8 | 110.6 | 1721.9 | 0.0 | 115.7 | 127.2 | 1736.3 |
| 86 | 110.9 | 0.0 | 55.2 | 1606.5 | 0.0 | 71.8 | 120.1 | 1692.0 | 0.0 | 120.6 | 147.0 | 1740.0 | 8.2 | 0.0 | 68.8 | 1618.7 | 113.4 | 0.0 | 68.5 | 1606.4 |
| 87 | 131.7 | 0.0 | 32.8 | 1610.4 | 0.0 | 61.0 | 75.4 | 1686.0 | 0.0 | 101.5 | 108.5 | 1725.5 | 36.5 | 0.0 | 22.3 | 1622.3 | 118.4 | 0.0 | 29.5 | 1612.0 |
| 88 | 0.0 | 104.6 | 134.8 | 1745.4 | 101.6 | 0.0 | 38.0 | 1635.8 | 0.0 | 12.3 | 46.9 | 1651.3 | 0.0 | 104.3 | 132.7 | 1745.5 | 0.0 | 104.5 | 134.7 | 1745.2 |
| 89 | 0.0 | 99.2 | 127.6 | 1706.1 | 83.7 | 0.0 | 35.5 | 1599.1 | 1.0 | 0.0 | 36.2 | 1606.4 | 0.0 | 106.0 | 132.0 | 1712.3 | 0.0 | 100.9 | 128.1 | 1707.3 |
| 90 | 0.0 | 102.5 | 123.7 | 1722.7 | 92.0 | 0.0 | 39.6 | 1610.7 | 0.0 | 4.4 | 44.6 | 1623.9 | 0.0 | 109.8 | 124.7 | 1729.0 | 0.0 | 120.2 | 142.0 | 1740.8 |
| 91 | 0.0 | 104.0 | 129.9 | 1711.6 | 0.0 | 18.3 | 42.2 | 1623.9 | 97.8 | 0.0 | 40.2 | 1595.0 | 0.0 | 85.5 | 109.8 | 1692.3 | 0.0 | 121.7 | 146.8 | 1730.3 |
| 92 | 0.0 | 122.2 | 160.7 | 1720.4 | 4.0 | 0.0 | 37.7 | 1599.9 | 0.0 | 7.5 | 54.7 | 1603.2 | 0.0 | 119.8 | 156.3 | 1718.9 | 0.0 | 64.9 | 105.6 | 1662.5 |
| 93 | 0.0 | 107.3 | 130.5 | 1713.6 | 89.2 | 0.0 | 29.3 | 1602.0 | 0.0 | 8.4 | 41.9 | 1614.9 | 0.0 | 110.2 | 129.8 | 1715.7 | 0.0 | 108.9 | 131.4 | 1714.8 |
| 94 | 0.0 | 81.4 | 111.7 | 1723.6 | 75.0 | 0.0 | 35.7 | 1636.8 | 0.0 | 6.1 | 46.2 | 1648.5 | 0.0 | 114.8 | 142.0 | 1756.1 | 0.0 | 118.5 | 149.1 | 1761.0 |
| 95 | 0.0 | 60.9 | 71.9 | 1690.8 | 30.5 | 0.0 | 62.9 | 1631.3 | 83.8 | 0.0 | 30.0 | 1628.3 | 0.0 | 91.6 | 130.0 | 1730.2 | 0.0 | 111.3 | 139.7 | 1749.2 |
| 96 | 0.0 | 113.1 | 145.3 | 1749.6 | 64.7 | 0.0 | 41.4 | 1634.1 | 15.8 | 0.0 | 48.2 | 1635.3 | 0.0 | 105.3 | 135.1 | 1741.6 | 0.0 | 115.2 | 147.6 | 1751.8 |
| 97 | 0.0 | 108.9 | 122.1 | 1728.0 | 18.4 | 0.0 | 70.1 | 1623.0 | 55.8 | 0.0 | 42.0 | 1610.5 | 0.0 | 82.6 | 106.6 | 1712.1 | 0.0 | 103.6 | 125.3 | 1732.8 |
| 98 | 0.0 | 85.7 | 105.5 | 1709.7 | 45.2 | 0.0 | 21.0 | 1618.4 | 28.6 | 0.0 | 42.1 | 1625.4 | 0.0 | 113.5 | 129.3 | 1735.8 | 0.0 | 119.3 | 139.0 | 1743.3 |
| 99 | 0.0 | 119.7 | 130.4 | 1746.3 | 0.0 | 20.5 | 30.3 | 1645.6 | 104.5 | 0.0 | 66.1 | 1618.8 | 0.0 | 80.0 | 88.3 | 1706.1 | 0.0 | 122.4 | 132.7 | 1749.5 |
| 100 | 0.0 | 119.3 | 135.9 | 1734.0 | 38.4 | 0.0 | 19.5 | 1611.6 | 44.1 | 0.0 | 42.3 | 1612.0 | 0.0 | 105.4 | 119.0 | 1719.4 | 0.0 | 113.8 | 129.9 | 1728.2 |
| **Median [Range]** | **21.2 [0, 141]** | **63.4 [0, 137]** | **104.3 [0, 183]** | **1691.7 [1595, 1793]** | **26.0**  **[0, 114]** | **34.8 [0, 115.5]** | **70.5 [2, 181]** | **1663.9 [1599, 1773]** | **32.0**  **[0, 135]** | **39.0**  **[0, 134]** | **73.5 [0, 177]** | **1666.5 [1595, 1797]** | **0.7 [0, 37]** | **69.8 [0, 123]** | **105.8 [22, 173]** | **1700.6 [1614, 1786]** | **25.3 [0, 129]** | **67.5 [0, 136]** | **106.6 [26, 182]** | **1696.9 [1599, 1792]** |

**Fig A. Statistical differences in migratory prevalence between extinct and extant ungulates.** Comparing the proportion of migratory species between extinct and extant ungulates, we found that extinct species were disproportionately migratory, considering (a-c) all ungulate species and (d-f) just African species. Extinct ungulates were significantly more migratory relative to extant taxa across all 100 tree topologies, both globally and within Africa (phyloGLM). Proportions in (a) and (d) reflect the median values across tree topologies, with bars colored by migratory status: *red bars* reflect the proportion of migratory species and *black bars* reflect the proportion of non-migratory species. Values in (b) and (e) reflect the difference in migratory prevalence between extant and extinct species, with the *black line* reflecting the median difference across all tree topologies. Note that values are always positive, indicating that migratory behavior was always more prevalent amongst extinct species. Values in (c) and (f) reflect the *p*-values associated with those differences; as in (b) and (e), the *black line* reflects the median across reconstructions, while the *dashed grey line* represents a *p*-value of 0.05, the conventional threshold for statistical significance. Map graphics were generated using the R package ‘maps’ v. 3.4.3 [19]. The data underlying this figure can be found at [doi.org/10.5061/dryad.jm63xsjsp](http://doi.org/10.xxx/dryad/zenodo.xxxxx.).

**Fig B. Statistical differences in macroevolutionary parameters between migratory and non-migratory ungulates including extinct ungulates.** Using phyloGLM to compare the parameter estimates that resulted from fossil-inclusive HiSSE modeling between migratory and non-migratory species, we found that migratory lineages exhibited significantly higher speciation and extinction rates in nearly all trees, and consequently higher lineage turnover and generally higher net diversification rates, than non-migratory lineages, though effects of migration on extinction fraction were inconsistent across tree topologies. In (a), (d), (g), (j), and (m), *red bars and curves* correspond to the distribution of parameter means (within-tree averages) for migratory species across all 100 trees and *black bars and curves* correspond to distributions of parameter means for non-migratory species. *Red and black lines* below the x-axes reflect 95% confidence intervals on mean parameter estimates across all 100 trees for migratory and non-migratory lineages respectively. Values in (b), (e), (h), (k), and (n) reflect the distribution of mean differences in parameter estimates between migratory and non-migratory species across all 100 trees, with the *black line* reflecting the median difference across all tree topologies and the *dashed grey line* representing a difference 0, where parameter values are equivalent between migratory and non-migratory lineages. Note that most values in (b), (e), (h), and (k) are positive, indicating that migration enhances speciation, extinction, lineage turnover, and net diversification rates, whereas values in (n) range from positive to negative, indicating that migration does not have a consistent effect on extinction fraction across tree topologies. Values in (c), (f), (i), (l), and (o) reflect the *p*-values associated with those differences; again, the *black line* reflects the median across reconstructions, while the *dashed grey line* represents a *p*-value of 0.05, the conventional threshold for statistical significance. The data underlying this figure can be found at [doi.org/10.5061/dryad.jm63xsjsp](http://doi.org/10.xxx/dryad/zenodo.xxxxx.).

**Fig C. Extant-only Hidden State Speciation and Extinction (HiSSE) model estimates of evolutionary parameters for migratory and non-migratory species across all 100 tree topologies.** In nearly all topologies, migratory species exhibited significantly higher (a) speciation rates and (d) net diversification rates but lower (b) extinction rates and (e) extinction fractions than did non-migratory species. (c) The effect of migration on lineage turnover rates varied across tree topologies. *Red bars and curves* correspond to distributions of parameter means (within-tree averages) for migratory species for all 100 trees and *black bars and curves* correspond to distributions of parameter means for non-migratory species. *Red and black lines* below the x-axes reflect 95% confidence intervals on mean parameter estimates across all 100 trees for migratory and non-migratory lineages respectively. Rates (a-d) are measured in units of species lineage^-1^ Ma^-1^, while extinction fraction (e) is unitless. The data underlying this figure can be found at [doi.org/10.5061/dryad.jm63xsjsp](http://doi.org/10.xxx/dryad/zenodo.xxxxx.).

**Fig D. Statistical differences in macroevolutionary parameters between migratory and non-migratory ungulates considering only extant ungulates.** Using phyloGLM to compare the parameter estimates that resulted from extant-only HiSSE modeling between migratory and non-migratory species, we found that migratory lineages continued to exhibit significantly higher speciation and net diversification rates in nearly all trees, whereas migration generally reduced extinction rates and extinction fractions and had inconsistent effects on lineage turnover rates in extant-only HiSSE models. In (a), (d), (g), (j), and (m), *red bars and curves* correspond to the distribution of parameter means (within-tree averages) for migratory species across all 100 trees and *black bars and curves* correspond to distributions of parameter means for non-migratory species. *Red and black lines* below the x-axes reflect 95% confidence intervals on mean parameter estimates across all 100 trees for migratory and non-migratory lineages respectively. Values in (b), (e), (h), (k), and (n) reflect the distribution of mean differences in parameter estimates between migratory and non-migratory species across all 100 trees, with the *black line* reflecting the median difference across all tree topologies and the *dashed grey line* representing a difference 0, where parameter values are equivalent between migratory and non-migratory lineages. Note that most values in (b) and (k) are positive and those in (e) and (n) are negative, indicating that migration enhances speciation and net diversification rates and reduces extinction rates and extinction fraction, whereas values in (h) range from positive to negative, indicating that migration does not have a consistent effect on lineage turnover rates across tree topologies. Values in (c), (f), (i), (l), and (o) reflect the *p*-values associated with those differences; again, the *black line* reflects the median across reconstructions, while the *dashed grey line* represents a *p*-value of 0.05, the conventional threshold for statistical significance. The data underlying this figure can be found at [doi.org/10.5061/dryad.jm63xsjsp](http://doi.org/10.xxx/dryad/zenodo.xxxxx.).

**Fig E. Fossil-inclusive Hidden State Speciation and Extinction (HiSSE) model estimates of evolutionary parameters for all four states (hidden and observed) across all 100 tree topologies.** *Solid curves* correspond to distributions of parameters estimates for the hidden state ‘A’ and *dashed curves* correspond to distributions of parameter estimates for the hidden state ‘B’; *red curves* correspond to distributions of parameter estimates for the migratory state and *black curves* correspond to distributions of parameter estimates for the non-migratory state across all 100 tree configurations. Parameter estimates for migratory and non-migratory lineages were generally more similar and less variable within hidden state B than within hidden state A, except for net diversification rates. Rates (a-d) are measured in units of species lineage^-1^ Ma^-1^, while extinction fraction (e) is unitless. Note the inconsistent x-axis scales across panels. The data underlying this figure can be found at [doi.org/10.5061/dryad.jm63xsjsp](http://doi.org/10.xxx/dryad/zenodo.xxxxx.).

**Fig F. Extant-only Hidden State Speciation and Extinction (HiSSE) model estimates of evolutionary parameters for all four states (hidden and observed) across all 100 tree topologies.** *Solid curves* correspond to distributions of parameters estimates for the hidden state ‘A’ and *dashed curves* correspond to distributions of parameter estimates for the hidden state ‘B’; *red curves* correspond to distributions of parameter estimates for the migratory state and *black curves* correspond to distributions of parameter estimates for the non-migratory state across all 100 tree configurations. Differences between all states tended to be less consistent in extant-only analyses. Rates (a-d) are measured in units of species lineage^-1^ Ma^-1^, while extinction fraction (e) is unitless. Note the inconsistent x-axis scales across panels. The data underlying this figure can be found at [doi.org/10.5061/dryad.jm63xsjsp](http://doi.org/10.xxx/dryad/zenodo.xxxxx.).

**REFERENCES**

1.	O’Brien K, Podkovyroff K, Fernandez DP, Tryon CA, Cerling TE, Ashioya L, et al. Limited herbivore migration during the Last Glacial Period of Kenya. Nat Ecol Evol. 2024;8: 1191–1198. doi:10.1038/s41559-024-02413-9

2.	Turner A. Preliminary evidence for seasonal deposition patterns from member 2 of the Swartkrans hominid site, South Africa. Journal of Archaeological Science. 1985;12: 163–175. doi:10.1016/0305-4403(85)90018-4

3.	Jefferson GT, Goldin JL. Seasonal Migration of *Bison antiquus* from Rancho La Brea, California. Quaternary Research. 1989;31: 107–112. doi:10.1016/0033-5894(89)90089-6

4.	Feranec RS, Hadly EA, Paytan A. Stable isotopes reveal seasonal competition for resources between late Pleistocene bison (*Bison*) and horse (*Equus*) from Rancho La Brea, southern California. Palaeogeography, Palaeoclimatology, Palaeoecology. 2009;271: 153–160. doi:10.1016/j.palaeo.2008.10.005

5.	Hardy F. Stable Isotope Analysis of *Bison latifrons* and Paleoecological Inferences. UNLV Theses, Dissertations, Professional Papers, and Capstones. 2015. doi:10.34917/7777306

6.	Glassburn CL. A reconstruction of steppe bison mobility in the Yukon-Tanana uplands and implications for prehistoric human behavior. Thesis. 2015. Available: https://scholarworks.alaska.edu/handle/11122/6192

7.	Pérez-Crespo VA, Schaaf P, Solís-Pichardo G, Arroyo-Cabrales J, Torres-Hernández JR. Mobility studies in fossil mammals from Laguna de las Cruces, San Luis Potosí, México: The strontium isotopic analyses. Journal of South American Earth Sciences. 2020;103: 102759. doi:10.1016/j.jsames.2020.102759

8.	Boeskorov G. Woolly rhino (*Coelodonta antiquitatis*) distribution in Northeast Asia. Deinsea. 2001;8: 15–20.

9.	Marean CW. Hunter–Gatherer Foraging Strategies in Tropical Grasslands: Model Building and Testing in the East African Middle and Later Stone Age. Journal of Anthropological Archaeology. 1997;16: 189–225. doi:10.1006/jaar.1997.0309

10.	Faith JT, Potts R, Plummer TW, Bishop LC, Marean CW, Tryon CA. New perspectives on middle Pleistocene change in the large mammal faunas of East Africa: *Damaliscus hypsodon* sp. nov. (Mammalia, Artiodactyla) from Lainyamok, Kenya. Palaeogeography, Palaeoclimatology, Palaeoecology. 2012;361–362: 84–93. doi:10.1016/j.palaeo.2012.08.005

11.	Hoppe KA, Koch PL. Reconstructing the migration patterns of late Pleistocene mammals from northern Florida, USA. Quaternary Research. 2007;68: 347–352. doi:10.1016/j.yqres.2007.08.001

12.	Pérez-Crespo AV, Cienfuegos-Alvarado E, Schaaf P, Solís-Pichardo G, Otero FJ, Torres-Hernández JR. Feeding habits and mobility of *Equus conversidens* and *E. mexicanus* from La Presita Blanca, State of San Luis Potosí, México. Historical Biology. 2022;34: 216–225. doi:10.1080/08912963.2021.1909008

13.	Faith JT, Thompson JC. Fossil evidence for seasonal calving and migration of extinct blue antelope (*Hippotragus leucophaeus*) in southern Africa. McGeoch M, editor. J Biogeogr. 2013;40: 2108–2118. doi:10.1111/jbi.12154

14.	Douw DS, Giltaij TJ, Kootker LM, Reumer JWF, Monaghan NT, Schulp AS. Investigating seasonal mobility in Irish giant deer *Megaloceros giganteus* (Blumenbach, 1799) through strontium isotope (87Sr/86Sr) analysis. Journal of Quaternary Science. 2022;37: 1348–1358. doi:10.1002/jqs.3447

15.	Lüdecke T, Mulch A, Kullmer O, Sandrock O, Thiemeyer H, Fiebig J, et al. Stable isotope dietary reconstructions of herbivore enamel reveal heterogeneous savanna ecosystems in the Plio-Pleistocene Malawi Rift. Palaeogeography, Palaeoclimatology, Palaeoecology. 2016;459: 170–181. doi:10.1016/j.palaeo.2016.07.010

16.	Woodruff AL, Schubert BW. Seasonal denning behavior and population dynamics of the late Pleistocene peccary *Platygonus compressus* (Artiodactyla: Tayassuidae) from Bat Cave, Missouri. PeerJ. 2019;7: e7161. doi:10.7717/peerj.7161

17.	Copeland SR, Cawthra HC, Fisher EC, Lee-Thorp JA, Cowling RM, le Roux PJ, et al. Strontium isotope investigation of ungulate movement patterns on the Pleistocene Paleo-Agulhas Plain of the Greater Cape Floristic Region, South Africa. Quaternary Science Reviews. 2016;141: 65–84. doi:10.1016/j.quascirev.2016.04.002

18.	Singh NJ, Börger L, Dettki H, Bunnefeld N, Ericsson G. From migration to nomadism: movement variability in a northern ungulate across its latitudinal range. Ecological Applications. 2012;22: 2007–2020. doi:10.1890/12-0245.1

19.	Becker RA, Wilks AR, Brownrigg R, Minka TP, Deckmyn A. Package “maps.” 2025. Available: https://github.com/adeckmyn/maps
