# Supplementary material for: Phylogenetic evidence that migratory behavior increases ungulate extinction rates
Source: PLoS Biol. 2026 Nov 5;24(11):e3004072. doi: 10.1371/journal.pbio.3004072 (PMC13739097; doi:10.1371/journal.pbio.3004072)
Supplement: Supplementary file 2 [file pbio.3004072.s005.docx]

10 November 2025

Dr. Nonia Pariente, Editor-in-Chief

*PLOS Biology*

Dear Dr. Pariente,

Thank you for the opportunity to submit the reviews we received from *PNAS* on our manuscript titled, “Migratory behavior increases extinction risk within ungulates” (*PNAS* Tracking #: 2025-22062). Our manuscript underwent two rounds of review at *PNAS* before being rejected; the reviews included below are from the second round of review.

One reviewer (reviewer 2) indicated that their concerns had been fully addressed and suggested only minor additional clarifications, which we have incorporated. The other two reviewers’ outstanding concerns primarily focused on (1) the novelty of the study and (2) our treatment of migration as a binary trait despite considerable intraspecific variation. In our responses and in the revised manuscript, we have addressed both of these points. We emphasize that treating migration explicitly as an evolving trait in a macroevolutionary framework is itself novel. Additionally, our principal findings—the broader distribution of migratory behavior among extinct ungulates, migration’s consequences for ungulate macroevolutionary dynamics, and the impacts of these extinctions for functional diversity—all represent new insights within the fields of ecology and evolution, with important implications for our understanding of both contemporary and historical ecosystem dynamics. We also clarify that coding migration as a binary trait is the most appropriate approach given its inconsistent and anthropogenically modified expression across populations, variation which is poorly documented. Our inclusive criteria thus provide an objective framework for characterizing species-level variation in migratory behavior, particularly in light of human influences. We have also clarified in our responses below our rationale for removing analyses of population trajectories in extant taxa, included in a prior iteration of this manuscript but removed from the present version, an omission about which Reviewer 1 expressed concerns. Importantly, we clarify that these analyses were excluded not because they produced non-significant results, but because we came to realize that they were not directly relevant to the central macroevolutionary questions addressed in the study. Their removal makes for a tighter, more focused manuscript. Reviewer 3 additionally suggested that we provide further methodological detail and greater transparency about our analytical decisions, which we have done in this submission.

We believe these revisions substantially improve the clarity of the manuscript. We are grateful to the reviewers at *PNAS* for their constructive feedback and hope our responses address their concerns. We appreciate your consideration of our manuscript for publication in *PLOS Biology*.

Sincerely,

Joel O. Abraham

(*on behalf of all authors*)

*Reviewer #1*

*Comments:*

*In this manuscript the authors primarily examine the impact of migratory behavior on extinction, speciation, and diversification in ungulate mammals using phylogenetic comparative methods. They find that being migratory appeared to increase both speciation and extinction rates of lineages, leading to a very small difference in diversification rates. I previously reviewed this manuscript for PNAS. I had somewhat mixed feelings about the original submission, primarily because some aspects of the comparative methods were not fully explained (and phylogenetic methods were not applied in key analyses). I think that in some ways the revised manuscript has improved considerably, but in other ways it has become substantially worse.*

We thank the reviewer for their continued thoughtful engagement with our manuscript. We are pleased that they found our revisions to have considerably improved some aspects of the work, and we appreciate their frank feedback on parts they felt had become less compelling. We address each of their concerns in detail below and have worked to further clarify several key decisions (including our categorization of migration as a binary trait and the classification of infrequent migrants as migratory) in this revised version.

*On the positive side, I think that my methodological concerns about the phylogenetic analyses have generally been well addressed, including the application of phylogenetic comparative methods to most analyses.*

We are very glad to hear that our revisions have addressed this reviewer’s methodological concerns. We agree that these modifications to our analyses have strengthened the paper, and we sincerely thank them for the constructive suggestions that led us to make these changes.

*On the negative side, the authors have removed the two sets of analyses that related to extinction risk among extant populations (the analyses of IUCN threat levels and of population trends over time). In some ways, removing these analyses might seem reasonable, because the results were largely negative (i.e. migration behavior did not predict IUCN threat level or population trends). In other ways, the removal of those analyses and results is problematic. Basically, the paper still implies (in the title, end of the abstract, end of the discussion, significance statement) that migratory behavior impacts current extinction risk. But the analyses that were removed failed to consistently support that idea, and so the authors removed them. Overall, this seems a bit misleading. I find it difficult to support publication of this manuscript under these circumstances.*

We thank this reviewer for raising this important concern. As we noted in our previous response letter, our decision to remove the IUCN threat level and population trajectory analyses was not because the results were non-significant, but because we came to question both the reliability of the underlying data and the relevance of those analyses to our study’s core focus. We are not trying to obfuscate or conceal from the reader any null results; we removed these analyses because we felt these null results were not reliable and were not in line with the core focus of the present study.

It is well-established that migration in the Anthropocene exposes ungulates to heightened risk, as we discuss extensively in the *Introduction*. The fact that this pattern was not reflected in the IUCN and population-trend analyses prompted us to reconsider what those analyses actually capture. In our initial submission to *PNAS*, we explicitly noted that the IUCN results likely reflected data limitations and interpretive issues rather than a true absence of effect. We explained this in detail in the prior version of the *Discussion* but removed that text when we decided to omit the IUCN analyses: “In seeming contradiction of our other results, we found that migratory ungulates are no more likely than non-migratory species to be at risk according to IUCN classifications (**Fig. 3**). However, this apparent discrepancy likely results from a combination of the fact that many migratory species have already gone extinct, as our reconstructions of migratory behavior in many extinct ungulates demonstrate (**Fig. 1**), as well as the coarseness of these categories. For instance, while extant migratory species may indeed be at greater risk of extinction relative to non-migratory species (**Fig. 2**), their species-level extinction may not be imminent. Indeed, the population densities of most ungulate species, migrants and non-migrants alike, appear to be fairly stable or even increasing (a testament to the efficacy of multiple decades of conservation efforts; 9, 59, 61) (**Fig. 3c**). Even those species that are in decline are experiencing fairly slow declines (**Fig. S3**), rather than precipitous declines that would warrant being listed as at high risk of extinction by the IUCN. In fact, extant migratory species continue to be some of the most abundant ungulates today (22, 23), and migratory species comprise nearly half of all extant ungulate species (14, 16). Thus, the instantaneous risk of extinction for extant migratory species may be fairly low, though still higher than that of non-migratory ungulates”.

Similarly, we chose to remove the population trajectory analyses because declining populations do not necessarily indicate elevated extinction risk. Population declines may result from short-term fluctuations or management changes rather than long-term vulnerability. As we stated in our previous response to reviewers, “a comparison of population trends among present-day species is not directly equivalent to our macroevolutionary analyses of speciation and extinction through time.” While population decline is a necessary precursor to extinction, it is not a sufficient predictor—many species experience temporary declines without going extinct (*e.g.*, Blois and Hadly 2009, *Annual Review of Earth and Planetary Science*).

Ultimately, we concluded that retaining these analyses risked conflating contemporary population dynamics with macroevolutionary processes, potentially obscuring our central question: whether migration influences extinction risk over deep time. Removing them allowed us to maintain a tighter and more coherent focus on macroevolutionary patterns, which are the primary scope of this paper. We stand by this decision, as we feel the present manuscript is more focused, cohesive, and precise without those analyses.

*Furthermore, it appears that the authors coded as migratory many species that are not generally considered migratory, based on using a very broad definition of migratory behavior. That is OK, but I think that the more reasonable thing to do would be to ensure that the conclusions do not hang on that particular definition of migratory behavior, and also include results based on a narrower, more traditional definition.*

We agree that definitions of migratory behavior vary widely across studies and that our approach could appear more inclusive or broader than some traditional definitions. We have therefore expanded and clarified our rationale throughout the manuscript.

As we have now elaborated in the *Introduction* (**lines 113-117**), we define migration inclusively as the capacity to migrate, considering a species migratory if any of its populations, at any point in time, exhibit seasonal round-trip movements between discrete areas. We take this approach because the present-day expression of migration across species and populations is shaped strongly by anthropogenic influences, a confound which we sought to circumvent: “Throughout, we define migration inclusively as the capacity to migrate; we consider a species migratory if any of its populations, at any point in time, exhibit seasonal round-trip movements between discrete areas. This definition captures the evolutionary potential for migration, recognizing that its expression today varies across space and time and is confounded by anthropogenic influences (2, 3, 17, 42, 43)”.

In the *Discussion* (**lines 243-255**), we further explain that this inclusive approach is biologically justified, and indeed necessary, because human activities have profoundly altered the expression and detectability of migration (by fragmenting landscapes, modifying resource distributions, and, in many cases, suppressing migratory behavior altogether). As a result, some species that are now weakly or facultatively migratory may have been strongly migratory prior to human impacts. Defining migration in this way thus provides an **objective and evolutionarily meaningful** framework that focuses on the *capacity* for migration rather than its contemporary distribution. We also note that rare or facultative migration can still have important evolutionary consequences, particularly because it often functions as an adaptive response to environmental extremes: “Our definition of migration was intentionally inclusive, designating a species as migratory if any population, at any point in time, exhibited seasonal round-trip movements between discrete areas. We adopted this approach because human activities have profoundly altered the expression and detectability of migration by fragmenting landscapes, modifying resource distributions, and in some cases suppressing migratory behavior altogether (8–14). As a result, some species that are now weakly migratory may have been strongly migratory prior to human impacts and vice versa (3, 55). Defining migration inclusively therefore represents an objective and biologically meaningful framework for macroevolutionary comparison, capturing the evolutionary capacity for migration rather than its contemporary expression. We recognize that the occurrence of migration may be infrequent or variable in some populations—some species we coded as migratory express this behavior only rarely (17, 56). Nevertheless, rare or facultative behaviors can still influence evolutionary outcomes (56, 57), particularly when they are adaptive responses to environmental extremes (58), as migration often is (59, 60)”.

Finally, in the *Methods* (**lines 338-347**), we clarify that our decision to code migratory behavior as binary was additionally due to the fact that population-level data are not available for most taxa. While this approach may slightly overestimate migration in some cases or underestimate it for data-deficient taxa, it ensures consistency and comparability across species and time periods, which is essential for macroevolutionary comparative analyses: “Migratory behavior varies substantially within species and across populations (with some populations of a species being entirely non-migratory and others almost completely migratory; 43) but is not well-catalogued at population scale. For this reason, and because anthropogenic factors such as barriers and resource modification have altered the distribution of migratory behavior in many taxa (2, 3, 42), migratory behavior was coded as binary: a species was considered migratory if it was explicitly described as such in published literature or if any population, past or present, exhibited seasonal round-trip movements between discrete ranges. While this inclusive classification may overestimate the importance of migration for some taxa or underestimate it for data-deficient ones, it provides a consistent framework for macroevolutionary comparison across taxa and time”.

For these reasons, we respectfully disagree with this reviewer that reanalyzing the data using a narrower, more restrictive definition would be appropriate. A narrower definition would depend on somewhat subjective criteria for inclusion (criteria which are not altogether obvious to us), would in fact *fail* to account for the dynamic and context-dependent nature of migration, and could obscure important evolutionary signal in the data.

*After removing the analyses of extant taxa and their current extinction risk, much of the manuscript comes down to macroevolutionary analyses of diversification in a modest-sized clade of mammals. But it is notable that the faster speciation and extinction rates in migratory species are nearly balanced with each other, such that there is almost no difference in diversification rates between migratory and non-migratory species (the difference in rates is only 0.01 species/Myr, but is stated to be significant).*

We thank the reviewer for this comment and the opportunity to clarify this point. We agree that the difference in net diversification rate between migratory and non-migratory species is small, and we explicitly acknowledge this in the text; as we note in the *Discussion* (**lines 285-289**), this modest difference likely reflects the fact that the elevated speciation and extinction rates among migratory species have largely balanced each other over time. This outcome is consistent with theoretical expectations: if one behavioral strategy led to substantially lower diversification, it would likely be much less prevalent today. Nevertheless, the significantly higher speciation *and* extinction rates among migratory lineages remain an important finding, indicating that migration is associated with elevated lineage turnover and, consequently, higher long-term risk. We interpret this as evidence that migratory behavior, while evolutionarily dynamic, also confers inherent instability for migratory lineages.

We also wish to emphasize that the analyses of macroevolutionary rates represent only one component of the study. The manuscript additionally includes two other key and complementary analyses: (1) the reconstruction of migratory behavior in extinct ungulates, and (2) assessments of how extinctions have shaped functional richness. As we elaborate in the *Discussion*, our behavioral reconstructions provide novel insights into the prevalence of migration among extinct species, with important implications for paleoecology and for understanding the evolutionary context of migration. The functional richness analyses further reveal that the extinction of migratory taxa has reduced the functional space occupied by ungulates, suggesting that past ecosystems, with more prevalent migration and thus greater connectivity, likely operated differently than contemporary ones.

*I was also very surprised that this manuscript was not cited and discussed: Beaulieu, J.M., and B.C. O'Meara. (2023). Fossils do not substantially improve, and may even harm, estimates of diversification rate heterogeneity. Systematic Biology, 72:50-61.*

We thank the reviewer for directing us to this paper, as we were not aware of it. We have cited it where relevant (*e.g.,* **line 458**) and included some discussion of it in our current submission.

*To be clear, the manuscript has other valuable aspects, such as the analyses of extinct taxa and of functional diversity. However, I am concerned about the extrapolation of the macroevolutionary results to living taxa, when the (now deleted) analyses of living taxa do not support this extrapolation. I also remain somewhat equivocal about whether the results are exciting and novel enough for PNAS.*

We hope that our responses above clarify our decision to remove the analyses of living taxa and demonstrate why this choice better aligns the manuscript’s scope and macroevolutionary focus. We also hope our explanations have underscored the novelty and broader significance of this work, particularly in treating with migration in a macroevolutionary context, identifying historical patterns of migration, and exploring the functional consequences of losing extinct ungulates.

*Reviewer #2:*

*Statistical or Methodological Comments:*

*I'm not an expert in phylogenetic analysis, but see that the other reviewer has expertise in this area. Thus, my comments focused on migration ecology and the (now omitted) population analysis.*

*Comments on Significance Statement:*

*The significance statement is well written and accurately summarises the research.*

We thank the reviewer for this positive feedback! Note that we have removed the significance statement, as *PLOS Biology* does not allow for one.

*Comments:*

*I think the authors did a thorough job addressing my comments, and I'm happy with the current draft of this manuscript. I appreciate the authors' decision to exclude the analysis of the extant population trends, as this was a relatively weak component of the previous draft, and detracted from overall significance of the other work. The current results hold up well on their own and make a strong and convincing case for migration being a fragile evolutionary strategy. I don't have any major additional comments.*

We are very thankful to the reviewer for their positive feedback. We are heartened to hear that they found the revised manuscript convincing and that the current version addresses their earlier concerns. We appreciate their supportive assessment and hope that the additional changes made in response to other reviewer comments further strengthen the manuscript and reinforce this reviewer’s enthusiasm about its publication.

*L295 "capacitate" -- > "enable" (for simplicity)*

We have changed the word ‘capacitate’ to ‘promote’ here.

*In Fig 3, I'm surprised that the variation explained in the PCA analysis, and included in the by x-y axis titles, are the same for Africa and Global, and would like to check if this is perhaps a typo?*

We thank the reviewer for pointing out this potential source of confusion. The values are correct; the proportion of variation explained in the global and African panels is the same because both are plotted in the same overall trait space derived from the full ungulate dataset. The Africa panels simply show the subset of taxa occurring in Africa within that same trait space. To clarify this, we now state this in the figure caption, “Panels (a) and (d) share the same trait axes, as do panels (b) and (e), because African species (d-e) represent a subset of global ungulate species richness (a-b) within the same trait space”.

*Reviewer #3:*

*Comments on Significance Statement:*

*They wrote this statement well, based on their work, although I have my skepticism on some of the conclusions simply because many steps taken and hard to follow some of them given word limitations and intricacies in decisions on how to handle data and the approaches. I commend the authors for doing so much work. Although interesting, I don't see this having wide applicability to other biologists, ecologists, conservationists etc. In my opinion, it quantifies what is already known, namely that migration is more risky that sedentary populations. I think the work is commendable, but not widely relevant or moving the ball much in this migration field, but potentially the phylogenetic field. Overall, the paper is methods rich, many of which were new to me, and likely to others in this field (sort of a niche subject). I did dig into this subject more to provide this review. The methods richness in itself is not a criticism, just a reality. They do many analyses, and have to use some subjective decisions to account for limited data and some analytical steps. I recommended in comments below for more transparency in the subjective decisions and assumptions. A reader would not be able to repeat methods as such. I found the work potentially publication worthy, but in a more specialized venue.*

We thank the reviewer for these thoughtful reflections and for engaging so deeply with the manuscript. We appreciate their recognition of the scope and effort involved in this project.

We respectfully note that, although it is widely accepted that migration can be risky in the context of human impacts, it is often assumed or implied that this is a modern, Anthropocene-specific phenomenon. In contrast, our goal here (as we state explicitly in the *Introduction*; see **lines 56-71**) was to test whether elevated extinction risk is a more general feature of migration across macroevolutionary timescales, independent of human influence. We were genuinely open to the alternative possibility (which we also outline in the *Introduction;* see **lines 75-78**) that migration might instead *reduce* macroevolutionary extinction risk given its known benefits for population size, connectivity, and gene flow. Yet our analyses indicated that migration is indeed associated with higher extinction risk even in deep time, which we view as an important and non-trivial finding.

We also wish to emphasize that treating migration explicitly as an evolving trait within a macroevolutionary framework is itself relatively novel. Only a small number of studies have approached migration in this way, and we believe this perspective provides new opportunities to quantify the evolutionary as well as ecological implications of migration (see also Abraham *et al.* 2022, *Nature Ecology & Evolution*).

In light of this comment, we have revised the manuscript to more clearly highlight the novelty of our approach and conclusions, and to provide additional transparency around methodological decisions to enhance clarity and reproducibility.

*Comments:*

*I appreciate the opportunity to review the paper led by J.O. Abraham et al. titled "Migratory behavior increases extinction risk within ungulates". My review is intended to be constructive and help the authors improve upon their interesting study.*

We thank the reviewer very much for their thoughtful and constructive review. Their feedback has indeed been very helpful for identifying areas where the manuscript could be clarified or strengthened. As we detail below, we have worked to incorporate their suggestions and further elaborate on our rationale for our analytical decisions.

*Main points*

*The analytical approach is complex, because of the different analyses and approaches taken within those analyses. I recommend that the authors include a paragraph within the MS that addresses main points of subjectivity, assumptions and potential weaknesses in their methods. Methods should be written so others can follow. While we all recognize the challenge and limitations of that, given the variety of steps within this approach, and subjectivity incorporated in the steps due to lack of information or nature of some analytical approaches, I feel this step justified. The intent is to help others improve understanding of what was done, potential limitations in the work, and help others using similar analytical methods to better build from. The authors do include some of this suggestion (Lines 374 - 384, 431-433, 268-274), yet I recommend a more thorough treatment.*

We have significantly expanded the *Methods* to provide additional detail about our analytical approach and to be transparent about the rationale behind all major decisions. We now explicitly highlight points of subjectivity, key assumptions, and potential limitations, with the goal of making our analytical approach more accessible and reproducible for other researchers using similar methods.

*Since authors will confront word limitations I also suggest locations to trim text.*

We have indeed trimmed the text in several places to accommodate the expanded methodological detail.

*Introduction: Lines 87-100 can be halved, as much of the text is handled in other papers and this text is repetitive. I'd simply make the point that migration a precarious strategy, and focus on the time scale of analyses. You could also merge in lines 131-141 here, so readers know immediately what you plan to do and why.*

We thank the reviewer for this suggestion. This section establishes the key hypotheses we test in the manuscript: whether migration reduces or elevates extinction and speciation rates and how these dynamics shape lineage turnover. Because it outlines the conceptual basis for our analyses, we are hesitant to shorten it too much. That said, we have trimmed some redundancies and removed unnecessary detail to improve readability, while preserving the elements needed to orient the reader to the central questions we address in this study.

*Abstract reads very well. Nice job.*

We are grateful to the reviewer for this positive feedback!

*Minor critique: The first paragraph summarizing prior work does not suggest, in fact it makes clear that populations of migratory ungulates confront greater risks that sedentary ones. Afterall, as stated in this MS and prior work, many species have migratory and sedentary populations. Often when a migration goes extinct the population becomes sedentary in an area (if it doesn't disappear entirely), reducing it's abundance to what resources can support. Of the factors causing modern migrations, which are listed well in the first paragraph, only 2 could affect the historic ones - hunting and changing climates. I bristled at the sentence suggesting modern declines in ungulate migration could be a continuation of Anthropocene extinctions (94-95; also line 74). This makes it appear that modern extinctions are almost to be expected, when clearly this is not so, and you do a solid job of making this point elsewhere in the paper (such as line 307 - 309). I'd reword so this text isn't unintendedly used to justify contemporary extinctions of migratory populations (in other words, it's a natural process). The rate and scale are completely different as noted in the MS.*

We thank the reviewer for directing our attention to this. We most certainly did not intend to suggest that contemporary declines of migratory ungulates are to be expected or should be viewed as natural or unconcerning. We have revised the text (in this section and throughout) to make clear that while migration may inherently carry higher risk, its fragility has been greatly amplified in the Anthropocene by human activities. The reviewer is also correct that some of the pressures faced by modern migratory populations, such as fencing and anthropogenic land-use change, are unique to recent times. However, other challenges, including changing climates, shifting habitats, and overhunting, may also have affected migratory species prior to the expansion of human impacts. We now elaborate on this distinction in the *Discussion* (**lines 261-272**).

*Similarly, lines 101 - 116 can be shortened, focusing on key points, as much text of this kind found elsewhere.*

We thank the reviewer for this suggestion. While some of the information in this section of the manuscript is indeed revisited elsewhere, this text provides the reader with the first full overview of our approach, and we feel it is important for orienting the rest of the manuscript. That said, we have shortened the text where possible to reduce redundancy while retaining key details necessary for clarity.

*Lines 126 - 130 Nice text.*

Thank you!

*Methods*

*Key variables are body mass, dietary grass fraction and latitude (which explains 44% of variation if migratory or not). How does these data square with some populations of the same species being migratory and others not? For the same species, if it migrates or remains sedentary often have the same values for the 3 variables. Recommend addressing in paper.*

As we discussed above in our response to similar feedback from reviewer 1, it is indeed true that migratory behavior can vary among populations within the same species. Understanding the causes of this intraspecific variation remains an active area of research. Current evidence suggests this is driven by differences in environmental conditions (*e.g.,* populations in more seasonal environments are more likely to migrate; Singh *et al.* 2012, *Ecological Applications*) but also cultural processes (Jesmer *et al.* 2018, *Science*) and anthropogenic influences such as fencing and land-use change (Harris et al. 2009, *Endangered Species Research*).

We are transparent about this complexity in the manuscript (see examples provided below). This is why, at the outset of the *Introduction*, we adopt an inclusive definition of migration as the *capacity* to migrate. While intraspecific variation is important, it is beyond the scope of this study, which focuses on the macroevolutionary consequences of migration. Macroevolutionary analyses inherently require working at broader taxonomic scales—in this case, the species level—as this is the level at which phylogenies are resolved and at which data can feasibly incorporate extinct species. Population-level data on migration and evolutionary relatedness are not available consistently even for extant species, let alone for extinct taxa.

For these reasons, although some species include both migratory and sedentary populations, we feel strongly that our species-level approach remains the most appropriate for addressing the macroevolutionary questions posed in this study.

Examples from the text:

“Throughout, we define migration inclusively as the capacity to migrate; we consider a species migratory if any of its populations, at any point in time, exhibit seasonal round-trip movements between discrete areas. This definition captures the evolutionary potential for migration, recognizing that its expression today varies across space and time and is confounded by anthropogenic influences (2, 3, 17, 42, 43)” (**lines 113-117**).

“Our definition of migration was intentionally inclusive, designating a species as migratory if any population, at any point in time, exhibited seasonal round-trip movements between discrete areas. We adopted this approach because human activities have profoundly altered the expression and detectability of migration by fragmenting landscapes, modifying resource distributions, and in some cases suppressing migratory behavior altogether (8–14). As a result, some species that are now weakly migratory may have been strongly migratory prior to human impacts and vice versa (3, 55). Defining migration inclusively therefore represents an objective and biologically meaningful framework for macroevolutionary comparison, capturing the evolutionary capacity for migration rather than its contemporary expression. We recognize that the occurrence of migration may be infrequent or variable in some populations—some species we coded as migratory express this behavior only rarely (17, 56). Nevertheless, rare or facultative behaviors can still influence evolutionary outcomes (56, 57), particularly when they are adaptive responses to environmental extremes (58), as migration often is (59, 60)” (**lines 243-255**).

“Migratory behavior varies substantially within species and across populations (with some populations of a species being entirely non-migratory and others almost completely migratory; 43) but is not well-catalogued at population scale. For this reason, and because anthropogenic factors such as barriers and resource modification have altered the distribution of migratory behavior in many taxa (2, 3, 42), migratory behavior was coded as binary: a species was considered migratory if it was explicitly described as such in published literature or if any population, past or present, exhibited seasonal round-trip movements between discrete ranges. While this inclusive classification may overestimate the importance of migration for some taxa or underestimate it for data-deficient ones, it provides a consistent framework for macroevolutionary comparison across taxa and time” (**lines 338-347**).

*359: Edit: coded as binary.*

We have revised this to read ‘coded as binary’.

*The paper identifies that climatic conditions affect migratory behavior. Methods describe estimating latitudinal range centroids for extinct ungulates, 63-10K years ago. Earth's climate has changed dramatically from then (say Paleocene) to present. Similar latitudes likely have different climates across time. How do you account for this in your analyses (please add text to paper).*

We thank the reviewer for this insightful comment and motivating us to clarify this point. This is precisely why we consider latitude to be the most appropriate metric, rather than attempting to incorporate direct climatic data. Earth’s climate has changed substantially even within the past 63,000 years, and it is not possible to reconstruct paleoclimate at the fine spatial and temporal resolution needed to infer migratory behavior; predicting migratory behavior from climate is challenging even with fine-scale modern data. Latitude, in contrast, provides a relative and internally consistent proxy for the magnitude of seasonality experienced by species. In addition, the core of species’ ranges tends to be relatively stable over time, even as climates fluctuate, making latitudinal centroids a more reliable proxy for the environmental conditions shaping migratory behavior across evolutionary time. While this approach cannot fully capture historical climatic variation, it allows for meaningful comparison across taxa and time. We have added this explanation to the *Methods*:

“Latitude provides a relative and internally consistent proxy for the magnitude of seasonality to which species are exposed (19), particularly given that Earth’s climate has varied markedly through geological time and fine-scale paleoclimatic reconstructions sufficient to infer migratory behavior are unavailable (17, 74)” (**lines 351-354**).

*Line 394. The sample size in the analyses got confusing for me here. Please clarify how these values relate to the 51 extinct species being analyzed (line 376 and 398) . What is the total sample of extant species?*

We have clarified the sample sizes in this section (see **lines 384, 393, 394, and 396**)

*Line 434: The production of polytomous clades is likely (my assumption here) from limited data. Valid? Regardless, how concerning is this? Please address in the paper.*

You are correct that our approach does produce polytomous clades due to the uncertainty regarding some of these relationships. As we note in **lines 420-424**: “Given that many placements produced polytomous clades, we used the ‘fix.poly’ function from the ‘RRphylo**’** R package to resolve these polytomies into dichotomies by assigning arbitrarily small branch lengths (0.0001) to newly created internodes (79). This entire process was repeated across all 100 trees to propagate phylogenetic uncertainty through our subsequent analyses.” Replicating analyses across 100 different tree topologies allows us to explicitly account for this uncertainty. By assessing the stability of results across many plausible phylogenetic scenarios, we ensure that our findings are robust to these uncertainties. For added transparency and reproducibility, all 100 tree topologies used in the analyses are provided in the Supplementary Materials.

*448: One value should have an <= or > = it seems.*

Thank you for noting this. We have changed this to ‘values < 0.5 were transformed to 0s (probable non-migrant) and values ≥ 0.5 were transformed to 1s (probable migrant)’ (**lines 434-435**).

*488: These Methods confused me, as it reads like analyses are circular. I don't believe they are. More explicitly, is diversification rate the independent variable here? But in lines 463 migration is used to assess if migration affects diversification rate. Hence the potential for circularity. Although line 236 explains the results well. Please clarify.*

We have revised this section of the *Methods* to clarify the independent and dependent variables for each analysis. In all cases, rate reconstructions resulting from HiSSE were used as the dependent variables, while species characteristics (migration, but also grass dependence, latitude, and body mass) were the independent variables. We compared the fit of the various models using AIC_c_ and, in the case of migration, additionally compared the magnitude of rate differences between migratory and non-migratory lineages.

*495: If species go extinct, wouldn't one expect functional richness to decrease? Why is this analyses important / interesting?*

Not necessarily—if the species that went extinct were functionally redundant (*i.e.,* possessed trait combinations already represented by extant species), functional richness might not have decreased. Such a scenario is plausible if species were driven to extinction by competition with ecologically similar species. However, our analyses show that functional richness does decline, indicating a lack of functional redundancy and a loss of ecosystem functions associated with the unique trait combinations represented by extinct taxa. We have clarified this in the manuscript (**lines 215-220**): “Migratory species and their unique combination of traits make them functionally important to the ecosystems they inhabit (4–6, 19). Yet it is unclear whether extinct migratory ungulates were functionally unique, characterized by distinct trait combinations, or were largely redundant with extant species. Thus, to assess the potential implications of these extinctions for ecological functioning, we quantified changes in ungulate functional diversity following the extinction of these species”.

*Clearly, you have spent a lot of time on your figures. These are good figures.*

We thank the reviewer for their kind words, we are glad they enjoyed our figures. We did indeed spend a good amount of time thinking about how best to visualize these results.

*Figure 3: a,b,d,e have no yellow. Why yellow in the key and not green for overlap? Yellow in c an d obviously. I would correct the key here. The axes identify the amount of variability explained. Values for a and d are identical, same with b and e. Data a subset of all data for d and e. Please check values to be sure this is correct. Seems strange that the axes values would be identical. Most axes (except one in a and d) do not explain much variability. Seems this should be addressed in the MS. Results not super compelling because of this. And if I am missing something then please make the point in the MS.*

We thank the reviewer for these detailed observations regarding Figure 3. As noted in the figure caption, “Because extant taxa are a subset of all taxa (*i.e.*, ‘extinct’ = extinct + extant), areas of overlap appear *green*”. We feel the use of green underscores the fact that extant species were also present with extinct species, such that ‘extinct’ represents the full trait space occupied when those taxa were around.

Regarding the repeated axis values: the proportion of variation explained in the global and African panels is the same because both are plotted in the same overall trait space derived from the full ungulate dataset, as we discussed above in our response to reviewer 2. To clarify this, we have now added to the caption: “Panels (a) and (d) share the same trait axes, as do panels (b) and (e), because African species (d-e) represent a subset of global ungulate species richness (a-b) within the same trait space.” We have also double-checked the values to ensure that they are correct.

Regarding the amount of variance explained: the axes together account for all variation across the trait dataset, although in some cases each individual axis explains a modest proportion. Importantly, these PCoAs are intended solely as a visualization tool to illustrate patterns of functional space and how they relate to underlying trait data. The percentage of variance explained by each axis does not influence the analytical results or their interpretation.

We hope these changes improve clarity and address the concerns expressed by this reviewer.

*Figure 4 referenced on line 326. Something missing or needs correction here.*

We thank the reviewer for identifying this typo. We meant to refer to figure 3 here. We have corrected this in our current submission.
