# Supplementary material for: Phylogenetic evidence that migratory behavior increases ungulate extinction rates
Source: PLoS Biol. 2026 Nov 5;24(11):e3004072. doi: 10.1371/journal.pbio.3004072 (PMC13739097; doi:10.1371/journal.pbio.3004072)
Supplement: Supplementary file 3 [file pbio.3004072.s006.docx]

18 June 2026

Dr. Ankiit Ahluwalia

Associate Editor, *PLOS Biology*

Dear Dr. Ahluwalia,

We are pleased to submit a revised version of our manuscript, “**Migratory behavior increases extinction risk within ungulates**” (PBIOLOGY-D-25-03497R1), for your consideration for *PLOS Biology*.

We appreciate the genuinely thoughtful and instructive feedback we received from the two reviewers, which focused largely on enhancing the rigor of our statistical approaches and the interpretation of our results. In particular, both reviewers had recommendations for improving the suitability of our hidden state speciation and extinction (HiSSE) analyses for addressing our central question, suggesting that we employ empirically derived sampling fractions and state-dependent extinction fractions. Reviewer 2 also suggested that we include further justification for our imputation-based approach for reconstructing migratory behavior in extinct ungulates and provided concrete ways to make our methodology more rigorous, such as explicitly testing different models of trait evolution rather than assuming evolution via Brownian motion.

In response to this feedback, we have, among other revisions:

1. Revised our phylogenetic imputation framework, conducting model selection between alternative models of trait evolution and using the best-supported model for reconstructing migratory behavior;
2. Calculated sampling fractions based on empirical data on extant and extinct ungulate diversity and clarified how they were employed in our models;
3. Implemented state-dependent extinction fractions, expanding our model set to include models in which all parameters were estimated simultaneously;
4. Repeated all HiSSE and associated analyses with and without extinct taxa to evaluate the sensitivity of results to the inclusion of extinct taxa;
5. Substantially expanded our discussion of the assumptions and limitations of both our imputation procedure and the HiSSE modeling framework.

It is clear that both reviewers wrote their reviews with the intention of informing and strengthening our analyses, and we are sincerely grateful for the constructive tone and content of their feedback; we feel that our analyses and conclusions have been substantially strengthened as a result. We hope that our responses and revisions fully address the concerns raised below, and we thank the journal and the reviewers for such a productive review experience.

Sincerely,

Joel Abraham, *Princeton University*

[joeloa@princeton.edu](mailto:joeloa@princeton.edu)

**Handling Editor:**

You'll see that both reviewers are positive and find the study interesting and worth pursuing, but they also raise several points that would need to be addressed before we can consider the manuscript for publication. Reviewer #1 main suggestions are to fit the same models without the fossil species to avoid biases that the fossil species might be incorporating to the structure of the data that would artificially reinforce the patterns. There are some concerns on how “sampling fractions” are calculated and it is linked with whether fossil species are included or not (see above). There are also suggestions to fit a model with all parameters free to be estimated, and then compare values and base their conclusions on the full set of models. Reviewer #2 main concerns are related to the interpretation of the HiSSE diversification results. Major concerns include problematic interpretations with conclusions from their HiSSE analyses that migratory lineages were associated with both higher extinction and higher speciation rates than non-migratory lineages. Another concern is related to assumptions of the phylogenetic imputations and suggested authors to clarify the assumptions and discuss the implications of their chosen model.

We thank you for this succinct summary of the major concerns of the two reviewers, it was very helpful for organizing our revisions. In our comments below, we respond to each of these concerns in turn and elaborate on how we have addressed them in our revised submission. We have, in brief:

1. Replicated our HiSSE analyses without fossil taxa;

2. Calculated sampling fractions from empirical data on ungulate diversity for both extant-only and fossil-inclusive HiSSE analyses;

3. Fit HiSSE models with all parameters (including extinction fraction) free to be estimated;

4. Substantially revised our interpretations of our HiSSE results and added caveats about parameter non-identifiability;

5a. Modified our approach for phylogenetically imputing migratory behavior, explicitly testing different models of trait evolution;

5b. More fully explained our rationale for our imputation-based approach and our treatment of migration as a continuous trait (which was indeed biologically motivated, as we elaborate below);

5c. Expanded our discussion of the limitations of both our imputation approach and HiSSE modeling framework.

We hope you feel, as we do, that these changes have fully addressed the concerns of the reviewers and improved the suitability of our manuscript for publication.

**Reviewer #1:**

This work intends to assess the impact of a very important behavioral trait (migration) in the extinction rates in ungulates. They use data from both extant and extinct species to estimate diversification rates and the occupation of the morphospace, using the results of these analyses to get a deeper understanding of the effects of migration. The manuscript is well written, with good contextualization and clear logic.

Thank you for your careful reading of our manuscript, as well as this succinct summary of our approach and your kind words about some of its strengths. Your review makes it clear that you thought deeply about our statistical approach and its ability to answer our central question; we are very grateful for the time you plainly invested in improving this work. We hope that our responses below make clear that we have taken your concerns seriously and that we have attempted to strengthen our analyses per your suggestions.

I must state from the beginning that I was not an original reviewer for this manuscript, neither here nor for the previous journal (as I found out after reading the reply letter). That being said, my comments will be mostly related to the implementation of the methods and the interpretation of the results. I believe the authors have overlooked a few caveats, as well as not interpreted the results as they should, and both of these issues might undermine the ability to draw conclusions from the results. I believe these issues are important enough to deserve a careful re-examination of the results in the light of my (hopefully useful) comments below.

We thank you for your constructive, thoughtful comments on both the implementation and interpretation of our methods, your feedback was indeed very useful. We appreciate your prompts to consider more deeply the analytical choices we made, particularly those involving our HiSSE analyses. We are somewhat new to state-dependent speciation and extinction approaches such as this and so welcome this feedback about best practices and how to make our implementation and interpretation of these approaches more robust.

Major points:

Title: I tend to concur with reviewer #1 about the title, because "extinction risk" is usually used in a context of conservation, which might lead to misunderstandings (I even informally got some feedback from colleagues - not disclosing what it was for nor revealing any information about the manuscript - and the impression was unanimously identical to mine. I would change it to "extinction probabilities" or "rates" or something along those lines.

A good point! We have modified the title to “Phylogenetic evidence that migratory behavior increases ungulate extinction rates”.

Lines 130-132: This comparison is tricky: I'd agree that this difference could mean something if the fossil species were distributed evenly across the phylogeny; since it isn't properly the case, there is a chance that the sample is biased (maybe migratory species have more chances of entering the fossil record by expanding the localities in which they can be preserved, or even some body size trends - if I'm not wrong, migrants tend to be larger, and being larger helps preservation). Thus, I would be careful to interpret this at face value.

We agree that taphonomic and phylogenetic biases in the fossil record could influence this comparison, particularly if traits associated with migratory behavior (*e.g.,* larger body size or broader geographic ranges) increase the likelihood of preservation or discovery. We have revised the manuscript to explicitly acknowledge this limitation and to avoid overinterpreting the difference (**L383–388**).

At the same time, we note that the pattern is qualitatively consistent in the African subset of the data, where coverage of fossil taxa is relatively complete. While this does not eliminate the possibility of biases within our sample of fossil taxa, it suggests that the observed pattern is unlikely to be driven solely by taphonomic processes. We have clarified this point in the revised text (**L388–391**) and now present this result more cautiously as being consistent with, rather than definitive evidence for, elevated extinction among migratory taxa (**L353–356**).

More generally, our goal in combining the imputation-based reconstruction of extinct taxa with HiSSE analyses was to evaluate whether independent lines of evidence suggest similar patterns. We agree that both approaches have important limitations, and we now make these caveats explicit in the manuscript (**L338–356, L357–371, L383–391**). We thus frame the results of our two approaches more cautiously as providing complementary and mutually consistent evidence (**L353–356**), rather than treating either analysis as definitive in isolation.

Lines 440-443: Some detail about the models could help, even if in the supplementary material.

Further details from these models, including effect sizes and *p*-values, are presented in **Figure S1**. We have added a reference to **Figure S1** here, on **L579 and L581**, as well as where we present these results **(L164)**.

Line 456: It would be important to fit the same models without the fossil species. The combined approach of Rphylopars+Threshold is basically a BM+Threshold (given that as far as I know Rphylopars does not have any OU model implemented for non-ultrametric trees), which would be the "continuous-zation" (for the lack of a better word) of an Mk model that is the same used in HiSSE. This way, by not assessing the results excluding the fossil species, you could be prone to biases that the fossil species are incorporating to the structure of the data that would artificially reinforce the patterns.

My feeling is that you can be artificially inflating extinction rates related to migratory species depending on how these species are distributed across the phylogeny. Based on Fig 1a, the fossils already show some phylogenetic bias, and that could be exacerbated if the clustering of migrant/non-migrant species is also important. It might be that the results hold, but if they are not super strong, maybe this artificial reinforcement is driving it to becoming "significant".

Thank you for this great suggestion for improving the robustness of our analytical approach! We have indeed replicated our approach for reconstructing macroevolutionary parameters (*i.e.,* selecting the appropriate model structure for parameter reconstruction, reconstructing parameters, and then testing how well all four traits predict parameters) with and without fossil taxa. In doing this, we found that the estimates of evolutionary parameters resulting from our HiSSE models do depend strongly on whether or not fossil taxa are included: with fossil taxa, we find that, as before, migratory ungulates exhibit elevated speciation and extinction rates, and consequently higher turnover and net diversification rates, though effects on extinction fraction are inconsistent across trees; considering only extant taxa, we find that, while migratory species continue to exhibit elevated speciation and net diversification rates, extinction rates and extinction fractions are lower within migratory lineages, and lineage turnover rates vary inconsistently across trees. We have substantially overhauled the manuscript, entirely rewriting the relevant sections of the *Results* and *Discussion*, to reflect these changes.

Lastly, this connects to the issue of fixing extinction fractions in a way (next point).

Please see our below comment regarding extinction fractions. We have indeed made our extinction fractions state-dependent, per your advice.

Lines 457-458: I think this is actually wrong. If I am not wrong, sampling fraction is typically calculated in relation to the total number of species in the clade, and was thought as a way to help inform the models about how many splitting events might be missing. Thus, most commonly the sampling fraction is calculated in relation to the total number of extant species in a phylogeny. Therefore, adding fossils to the phylogeny will not amount to the total extant richness which would be needed to calculate sampling fraction. If the authors really wanted to calculate an equivalent metric, they should consider all species from the group that ever lived. I don't know exactly how HiSSE deals with the fossil species, but this might have implications in the results in case the authors added the fossil species when calculating sampling fraction, given that the model will be misinformed that the data is closer to the true extant diversity than it actually is.

We thank you for your instructive and thoughtful feedback about the sampling fraction we employed in our previous iteration of these analyses. We agree that our previous implementation of the sampling fraction did not adequately reflect how this parameter ought to be defined in state-dependent diversification models, and we have modified our approach accordingly.

Following your suggestion and recent guidance (*e.g.,* Mynard *et al.* 2023, *Systematic Biology*), we now employ a sampling fraction of 0.1 for HiSSE models including extinct species and a sampling fraction of 0.75 for extant-only models (which we added in response to your above feedback about the potential biases introduced by the inclusion of fossil species). We defined these sampling fractions empirically. For analyses including extinct taxa, we defined the sampling fraction based on the proportion of extinct species meeting our inclusion criteria (*i.e.,* late Quaternary ungulates within extant families) that are represented in our dataset. We used the NOW Fossil Database to do this, which contains information about Cenozoic land mammal taxa and localities. Within this database, 1923 ungulate species are recognized from 23 Ma onwards within families containing extant representatives; 258 species, or 13%, are included in our phylogeny. For extant-only analyses, we defined the sampling fraction as the proportion of extant species included in the phylogeny we used. The IUCN recognizes 259 ungulate species, of which 207 (80%) are included in our phylogeny. In both cases, we followed Mynard *et al.* (2023) in conservatively rounding our sampling fractions downward, as their results “suggest that it is better to cautiously under-estimate sampling efforts”, since that is less likely to produce false positives. We applied a single sampling fraction across observed and hidden states, since the distribution of unsampled species across states is unknown.

We have updated the *Methods* to clarify these calculations (**L604–615**) and revised our *Results* to reflect this revised parameterization.

Lines 460-462: I wonder how holding extinction fractions constant could impact your conclusion given that the central message of the paper is related to extinction rate differences. I am fully aware that estimating extinction rates from phylogenies is tricky, and I am assuming that the authors made this decision to simplify the model, but as I said, given that the core of the paper is related to extinction rates, in my opinion holding the extinction fraction constant has a direct impact in the results and conclusions. I would therefore suggest that the authors fit a model with all parameters free to be estimated, and then compare AICc, parameter values, etc., and base their conclusions on the full set of models.

We thank the reviewer for this important suggestion. We agree that fixing the extinction fraction is not appropriate given that our central question concerns differences in extinction dynamics, and we have revised our modeling framework accordingly.

In the revised analyses, we allow both turnover and extinction fraction to vary across observed and hidden states, thus expanding the set of models we tested to include models in which all key diversification parameters are estimated simultaneously. As before, we compared models using AIC_c_ and based our interpretation on the full set of supported models.

These changes are now described in the *Methods* (**L604–605**), and the resulting parameter estimates (including extinction fraction) are presented explicitly in the revised *Results* and figures. While migration continues to increase both speciation and extinction rates, at least in our fossil-inclusive HiSSE models, we find that migration has an inconsistent effect on extinction fraction, with migratory species exhibiting increased extinction fractions in some tree topologies and reduced extinction fractions in others. We now present and interpret our HiSSE results with greater caution in light of the complexities associated with estimating extinction rates from phylogenies (see also response to related concerns from reviewer 2 below).

Line 470: They are also associated to the marginal probability of being on hidden state A or B. Therefore, the authors should show and compare the distribution of rates for each of the 4 estates (MigA, MigB, NMigA, NMig B). If they lump them all together, the purpose of using HiSSE is lost and the false positives might still be taking place.

Thank you for this suggestion. We now present and compare parameter distributions between both observed and hidden states in **Figures S5 and S6**.

Lines 479-481: One or more of those traits may be acting within migratory/non-migratory species, generating rate heterogeneity within each category and even driving the diversification patterns in lieu of the mapped trait (migratory behavior). That's the purpose of the hidden state, as stated in the previous point.

Indeed, that was exactly our rationale for testing whether using migration as a predictor of evolutionary rates, rather than one of these correlated traits, resulted in a better model fit (lower AIC_c_). We have revised this section of text to clarify the rationale for this set of analyses.

Line 492: I don't get how a trait space is constructed using the distances between pairs of species. To me, the distances come exactly FROM the trait space. This seems very circular and confusing.

Thank you for pointing out that our language in this section was confusing. You are correct that it was imprecise to refer to these ordinations as multidimensional trait spaces when in fact they are simply ordinations of our Gower distance matrices, generated for visualization purposes. We have revised this section to read: “we then visualized Gower distances between species in multidimensional trait space (**Fig. 3**) using ‘wcmdscale’ in the ‘vegan’ R package [92], which conducts principal coordinates analysis (PCoA) on the Gower distance matrix” (**L647–649**).

Figure 3: I understand that the overlap between yellow and blue becomes green, but I think the color in this figure could be rethought. I would represent the polygon for the extinct species on its own, separate from the extant, instead of having it as extinct + extant. It is easy enough to visualize the sum of everything as the union of both polygons, and by splitting the categories you add some more information in terms of whether the space has shifted, expanded, etc.

This is a great suggestion for improving the instructiveness of this figure! We have done exactly as suggested, depicting separate polygons for extinct species only and extant species only. We have also revised the figure caption accordingly.

Minor point:

Line 188: You could show the distributions of rate differences to highlight how distinct are the rates

The magnitude of rate differences across trees for all five rate classes are visualized in **Figure S2**. We have included more references to this figure where relevant throughout the main text (see **L218, L228**).

**Reviewer #2**

This manuscript tackles an important topic from an original angle, and I believe it deserves to be published. The central question, whether migratory behavior is causally linked to higher extinction rates, not only for extant species under Anthropocene global change but also at macroevolutionary timescales, is timely and clearly relevant. Climatic and environmental change, whether anthropogenic or driven by natural geological events, can disrupt resource-availability pathways by reducing availability and increasing unpredictability, and these effects may extend into deep evolutionary timescales. Under such conditions, the authors conclude, migration may enhance extinction risk over evolutionary time.

The authors first address whether migratory behavior was disproportionately represented among extinct species by compiling data on ungulate species that went extinct over the last ~63,000 years and using phylogenetic imputation to infer migratory behavior in these taxa. Because migration is difficult to infer directly from the fossil record, they rely on three continuous traits previously shown to correlate with migratory behavior in extant species. A literature-validation indicates support for the inferred migratory status in approximately 20% of extinct taxa. The authors then test for correlations between migratory behavior and tip diversification rates (speciation and extinction), inferred under a HiSSE model, using phylogenetic generalized linear models.

The authors conclude that migration is an evolutionarily fragile strategy, especially as increasingly widespread and intensive anthropogenic activity disrupts resource gradients (e.g., through infrastructure development) and increases environmental unpredictability (e.g., via climate change).

Overall, the methodology is sophisticated, the data appear robust, and the study addresses a biologically important question. However, I believe that some conclusions, specifically those related to diversification dynamics, are overstretched, given the well-known difficulties in disentangling speciation and extinction rates from extant-only phylogenies over deep macroevolutionary timescales. My main concern, therefore, relates to the interpretation of the HiSSE diversification results.

To clarify, I am relatively confident in the conclusion that a high proportion of extinct species were migratory, based on the phylogenetic imputation analyses. In contrast, I am less convinced by the inference that migratory lineages experimented higher extinction rates over evolutionary time and therefore that this is a labile macroevolutionary strategy. I suspect this might be an artifact of parameter non-identifiability in the diversification analyses. I note that my concerns are not related to the parameter non-identifiability highlighted by Louca and Pennell (Science, 2020) for continuous-time varying birth-death models. SSE models like HiSSE estimate parameters using numerical integration of ODEs rather than closed-form likelihoods expressed as deterministic lineage-through-time plots, as in Louca & Pennell, so the same identifiability issues do not directly apply.

We thank the reviewer for your careful and constructive evaluation of our manuscript, and for your positive assessment of its relevance and our overall approach. It is clear that you spent a lot of time reading and reflecting on our manuscript and that you thought deeply about how it could be improved, and we are very appreciative of your thoughtful engagement with our work. We are heartened that you find the central question timely and that the logic and structure of our analyses are clear. We particularly appreciate your detailed comments on the interpretation of the HiSSE results. Your concerns regarding parameter identifiability and the potential for overinterpretation of speciation and extinction rates were especially helpful in motivating us to re-evaluate both our modeling framework and how we present and discuss these results.

In response, we have made several substantive revisions to our analytical approach. We now explicitly acknowledge the limitations associated with disentangling speciation and extinction rates from phylogenetic data (**L343–353**). We have also updated our HiSSE analyses per your suggestions and those of the other reviewer to better align with current best practices, including: (i) recalculating sampling fractions from empirical data; (ii) allowing extinction fraction to vary between states; and (iii) replicating all diversification analyses using extant species only, allowing us to assess the sensitivity of our results to the inclusion of fossil taxa. We have also revised the framing of our conclusions to more explicitly distinguish between well-supported patterns (*e.g.,* differences in speciation and net diversification) and aspects that are more sensitive to model assumptions.

Below we respond to your specific comments in turn. We thank you again for your instructive feedback and hope it is clear from our revisions and responses that we have taken seriously your concerns.

Major points:

1. Speciation-extinction non-identifiability.

The authors conclude from their HiSSE analyses that migratory lineages were associated with both higher extinction and higher speciation rates than non-migratory lineages. They attribute this pattern to reduced access to seasonal resources under climatic and geological change, which may not compensate for the substantial energetic costs of migration (leading to extinction), combined with barriers to gene flow that isolate populations and promote speciation.

I find this interpretation problematic. Speciation and extinction are "notoriously difficult" to estimate separately from extant-only phylogenies. Consequently, most diversification software, including hisse, reparameterize these rates into composite parameters that behave better in likelihood space. In hisse, the core parameters are turnover: τ = λ+μ, and extinction fraction: ε = μ / λ, from which speciation and extinction are derived analytically. Although this reparameterization reduces pathological likelihood ridges caused by strong correlations between λ and μ, it also complicates biological interpretation.

Two results call my attention. First, net diversification rates are negative in both migratory and non-migratory ungulates (i.e., extinction exceeds speciation in both cases). Second, estimated extinction and speciation rates are very similar between the two categories, differing only slightly in absolute value (migratory: λ = 2.86, μ = 1.03; non-migratory: λ = 2.84; μ = 1.01). I suspect that these nearly identical trends and values indicate that speciation and extinction cannot be really disentangled in these analyses, a pattern that is consistent with my own experience using HiSSE implementations in a Bayesian framework (using RevBayes), where likelihood ridges often lead to non-identifiability of these parameters.

If I understand the authors' interpretation correctly, they argue that "higher turnover combined with lower net diversification" in migratory lineages indicates higher extinction rates relative to non-migratory lineages. However, high turnover combined with low net diversification indicates rapid lineage replacement, as the authors themselves note, but does not by itself provide strong evidence for elevated extinction (given the limited identifiability of extinction rates from extant-only phylogenies mentioned above). Only if extinction fraction were demonstrated higher in migratory lineages could one reasonably conclude that extinction contributed disproportionately to their diversification dynamics. Using the averaged speciation and extinction rates reported in lines 185-208, I calculate extinction fractions of 1.007 for migratory lineages 1.019 for non-migratory lineages, suggesting slightly higher relative extinction in non-migratory taxa, a result that contradicts the authors' conclusions. I could not find the extinction fraction reported in the manuscript. I therefore suggest that extinction fraction is displayed directly in Figure 2, together with net diversification, as it is more informative for interpreting extinction dynamics than turnover.

Also, how should the negative net diversification rates in both categories be interpreted biologically? And can the authors explain why confidence intervals are substantially wider for migratory species (Figure 2)?

Thank you for this series of careful and technically insightful comments, which motivated us to read up more on SSE frameworks and to rethink our analytical approach. We recognize that interpreting speciation and extinction rates within SSE frameworks requires caution, particularly when model constraints or parameter correlations limit the ability to distinguish among diversification components.

We would clarify, however, that in our original analyses extinction fraction (ε) was fixed across states. This constraint was not clearly described in the manuscript and likely contributed to the patterns you highlight: namely, the very similar estimates of speciation and extinction rates between migratory and non-migratory lineages and the limited impacts of migration on net diversification. Because ε was held constant, the model was effectively restricted in how it could partition differences among λ, μ, and net diversification, which in turn limited our ability to detect differences in extinction dynamics. For the same reason, our models did not produce state-specific estimates of extinction fraction, and we therefore did not report this parameter in the original manuscript.

In response to your comment (and that of reviewer 1), we have revised our HiSSE analyses to allow extinction fraction to vary between states. This change removes the constraint described above and allows extinction dynamics to be estimated more directly. We have updated the *Methods* and *Results* to reflect this revised framework (**L604–605**). Under this updated parameterization, we find that, for our fossil-inclusive HiSSE models, migratory lineages continue to exhibit elevated speciation and extinction rates, resulting in greater lineage turnover and net diversification rates. The effect of migration on extinction fraction, however, varies across trees, ranging from significantly positive to significantly negative. In extant-only HiSSE models, migration has less consistent effects on parameter estimates in general, though we find that migratory lineages generally continue to exhibit higher speciation and net diversification rates, whereas extinction rates and extinction fractions tend to be lower for migratory lineages and lineage turnover rates vary inconsistently between migratory and non-migratory lineages. We now report extinction fraction explicitly in **Figure 2** and base our interpretation on the full set of parameters, rather than solely on speciation and extinction rates in isolation.

More generally, we have revised the manuscript to present and discuss the diversification results with greater care, avoiding overinterpreting state-dependent rate differences or treating them as definitive evidence in isolation.

With respect to net diversification being negative, this is no longer the case with our updated analytical approach: in both fossil-inclusive and extant-only analyses, net diversification is positive and is higher for migratory lineages than non-migratory lineages. We discuss these patterns in the manuscript on **L403–419**.

Finally, we have clarified patterns of uncertainty in **Figure 2**. Confidence intervals are wider for migratory lineages (**L231–233**), which we attribute to their greater sensitivity to phylogenetic and imputation uncertainty: because extinct taxa are reconstructed as disproportionately migratory, these taxa have a disproportionate influence on parameter estimates for the migratory state. They are also the only taxa for which migratory state varies between trees (because the migratory states of extant taxa are known). Accordingly, variation between trees in which extinct taxa are reconstructed as migratory likely underlies the greater variation in rate estimations for the migratory state (**L372–382**). Consistent with this interpretation, confidence intervals are narrower for migratory taxa in extant-only analyses (**L248–249**), which we have now included as a sensitivity analysis (see also **L379–382**).

2. Phylogenetic imputation.

Phylogenetic imputation relies on the R package "phylopars", which performs multiple imputation and thus incorporates imputation uncertainty, but only for continuous traits. This limitation "forces" the authors to adopt an indirect approach: migratory behavior, a binary trait, is first coded as 0/1 and treated as continuous; imputed continuous values are then converted back to binary states using a fixed threshold value (> 0.5, migratory; < 0.5, non-migratory).

Although this approach is pragmatic and it gives intuitive results, I think it relies on strong assumptions about the underlying evolutionary process and also collapses probability uncertainty into point estimates. Brownian diffusion implies continuous, symmetric change and increasing variance with time. In addition, thresholding with a fixed value converts probabilistic estimates into fixed classifications, although in this case the authors use multiple imputation to incorporate uncertainty in downstream analyses. By contrast, a CTMC model (which treats migration as an intrinsically discrete trait) allows for instantaneous transitions between binary states, asymmetric gain and loss rates, and, by bypassing thresholding, retains uncertainty throughout the inference. I am not suggesting that this invalidates the authors' imputation results, but I suggest that the authors clarify the assumptions and discuss the implications of their chosen model. For example, do you think that there is an underlying continuous liability in migratory behavior, e.g. a "degree of migratoriness"?

Moreover, alternative software exists that can perform phylogenetic imputation directly for discrete traits with non-Gaussian distributions. For example, RevBayes can estimate posterior distributions for missing tip states under binary CTMC or multistate Markov models, with uncertainty propagation handled naturally through Bayesian joint modeling. Similarly, the R package MCMCglmm can accommodate binary traits via threshold models within a Bayesian framework.

Because phylogenetic imputation results depend strongly on the assumed evolutionary model, the manuscript should state explicitly which continuous trait evolution model was used in phylopars (I assume it was a Brownian motion model), and whether alternative models were explored (e.g., OU). As implemented, I understand that your analysis assumes a constant rate of trait evolution through time, but it is conceivable that this rate might have increased, decreased, or even approached zero during particular intervals of evolutionary history, in which case time-varying BM, Early Burst, or ACDC models might be more appropriate. Additionally, imputation assumes that missingness is approximately phylogenetically random. Are particular families or genera disproportionately represented among the extinct taxa in your compiled dataset?

I hope that these comments are helpful in further strengthening an already interesting and timely manuscript.

Thank you for this thoughtful and detailed comment. As with your prior comment, it motivated us to think deeply about our approach and how to improve it. We agree that our original description did not sufficiently clarify the assumptions underlying our imputation approach, and we have revised both our methodology for conducting imputation as well as how we present it in our revised submission (**L129–135, L554–576**).

First, in response to your suggestion, we have expanded our imputation framework to incorporate model selection. Specifically, we evaluated multiple models of trait evolution (Brownian motion, λ, κ, Early Burst, and star) and selected the best-supported model based on AIC_c_ prior to conducting imputation. Across all 100 tree topologies, the λ model was consistently favored, with estimated λ values ranging from 0.880 to 0.912, indicating slightly weaker phylogenetic signal than expected under Brownian motion. We now use this model for imputation and have revised the *Methods* (**L554–576**) and *Results* (**L129–135**) accordingly. Notably, this change strengthens the observed discrepancies between extinct and extant taxa, with an even greater proportion of extinct species inferred as migratory.

More broadly, we agree that treating a binary trait as continuous for imputation introduces assumptions that warrant explicit discussion. We have therefore expanded the manuscript to explicitly discuss the limitations and to clarify the rationale for this approach (**L357–391, L563–573**). From a methodological perspective, recent simulation work (*e.g.,* Gendre *et al.* 2024, *Methods in Ecology and Evolution*) suggests that treating discrete traits as continuous for phylogenetic imputation can yield low error rates, particularly when phylogenetic structure is strong. From a biological perspective, migratory behavior is widely recognized as existing along a continuum from obligate migration to facultative migration to residency, such that a ‘degree of migratoriness’ or ‘propensity to migrate’ is indeed biologically realistic (*e.g.,* Cagnacci *et al.* 2011 *Oikos*; Martin *et al.* 2018 *Scientific Reports*; Berg *et al.* 2019 *Frontiers in Ecology and the Environment*). Our approach is consistent with this conceptualization of migration: we impute a continuous quantity, akin to migratory propensity, and then apply a threshold to match the binary resolution of the available data for extant species. We acknowledge that this thresholding step collapses uncertainty (though we try to preserve some of this uncertainty by repeating our imputation procedure and downstream analyses across all 100 tree topologies), and we now explicitly discuss this limitation (**L369–372**), as well as the rationale for assuming a continuous underlying process (**L362–367**).

Finally, we clarify additional assumptions of the imputation procedure in the revised manuscript, including the distribution of missing data across the phylogeny. Though extinct taxa are not uniformly distributed across clades, they represent most major extant lineages (see **Fig. 1**), and we now explicitly acknowledge the potential influence of non-random missingness and its implications for interpretation (**L383–391**).

We thank you again for a thorough, instructive, thought-provoking review. We sincerely appreciate your feedback and feel our study is stronger for it.
