# Supplementary material for: Phylogenetic evidence that migratory behavior increases ungulate extinction rates
Source: PLoS Biol. 2026 Nov 5;24(11):e3004072. doi: 10.1371/journal.pbio.3004072 (PMC13739097; doi:10.1371/journal.pbio.3004072)
Supplement: Supplementary file 4 [file pbio.3004072.s007.docx]

9 September 2026

Dr. Ankiit Ahluwalia

Associate Editor, *PLOS Biology*

Dear Dr. Ahluwalia,

We are pleased to submit a revised version of our manuscript, “**Phylogenetic evidence that migratory behavior increases ungulate extinction rates**” (PBIOLOGY-D-25-03497R2), for your consideration for *PLOS Biology*.

We are grateful to the academic editor for making us aware that some of the diversification parameter estimates we recovered in the prior version of our manuscript seemed implausibly low. The academic editor suggested that we probe these results further to ascertain whether they were the result of a coding error or model specification error, which we have done. We have thoroughly checked our code and conducted several sensitivity analyses to identify the root of the issue: an ambiguity in the source code of the package that we used to conduct these analyses regarding the handling of extinct taxa. In our revised submission, we have accounted for this ambiguity and have re-run all the corresponding fossil-inclusive diversification analyses. Our corrected analyses yielded much more plausible estimates of all diversification parameters. Furthermore, we found that, though quantitatively the parameter estimates differed drastically from the prior version of our analyses, qualitatively our results were essentially unchanged: migratory lineages continue to exhibit elevated extinction and speciation rates, and thus higher lineage turnover and net diversification rates, than non-migratory lineages. We have amended the manuscript to reflect these updated results and have also added caveats to the text where relevant about not over-interpreting the parameter estimates resulting from such approaches, due to their sensitivity to model parameterization and specification.

We hope that our responses below and our revised analyses and manuscript fully address the concerns raised by the academic editor, and we thank them again for bringing this matter to our attention.

Sincerely,

Joel Abraham, *Princeton University*

[joeloa@princeton.edu](mailto:joeloa@princeton.edu)

**Academic Editor:**

After reviewing the revised version, I've identified a potentially significant issue with the new analysis. One of the central conclusions is that migratory species exhibit both higher speciation and extinction rates compared to non-migratory species, primarily based on the HiSSE analysis using the fossil-included phylogeny. My concern lies in the improbability of the absolute values for these estimates, which suggests that there may be an underlying problem with the analysis. The extinction rate estimate for migratory species is 8.759×10^-9 (0.000000008759), while for non-migratory species, it is 9.288×10^-10 (0.0000000009288). The authors assert that these rates are significantly different, acknowledging their small magnitude. For reasons I explain below, those estimates seem to indicate that something went wrong with the analysis. I have never seen estimates that low…. Moreover, even if there is no problem with the analysis itself, when we assess the biological significance of such low rates, their differences do not seem meaningful. Statistically, they may differ, but biologically, both amounts are effectively zero extinction given the time span of such lineages. That in itself is already enough to question the interpretations, and would require a different narrative, but I do suspect that the analysis has some problems.

At first glance, small estimates of extinction rate might seem expected since phylogenetic estimates often yield low extinction rates, but these values are exceptionally low. Firstly, the absolute values are virtually zero, that in combination with the estimated rate of speciation would result in a astronomically high number of species if retained for the duration of the clade (assuming, of course as a simple scenario, an exponential growth); secondly, strangely, the estimates of extinction rate when incorporating fossils result in extinction rates that are significantly smaller than those based solely on extant species by several orders of magnitudes (compare those rates to the rates of 0.118 and 0.013 when using phylogeny with only extant species). This outcome appears counterintuitive. Furthermore, how is it possible to obtain such low extinction rates when we know that several species (included in the phylogeny) are extinct? This strongly suggests that there was a problem in the estimation process. Given that a substantial part of their argument relies on this analysis, in the argument that migratory and non-migratory lineages have different extinction rates, and that these values hint at a problem, it is essential for the authors to revisit these estimates (possibly correcting a small coding error) and/or provide a justification for these unusual results, as well as their biological meaning for their interpretation, before the paper can be considered for publication.

We thank the editor for drawing our attention to the extremely small absolute extinction rate estimates resulting from our fossil-inclusive analyses and prompting us to probe these values further. We wholly agree that these estimates warrant scrutiny, and we have conducted a series of additional checks and sensitivity analyses to determine whether these values resulted from an error in our implementation or model specification. In short, we found that these values resulted from an ambiguity in the source code regarding how fossil taxa are handled by the ‘hisse’ package. Due to this ambiguity, the optimization algorithm pushed the parameter estimates towards the estimation lower-bound specified by ‘hisse’ (2.06×10^-9^). We have modified our code to account for this ambiguity and have re-run all fossil-inclusive diversification analyses. This has resulted in much more plausible estimates of all five diversification parameters. We have modified the text and figures accordingly to reflect these updated values (see **L196‒246** and **Fig. 2**). We have also added caveats to both the *Results* (**L192‒195**) and the *Methods* (**L640‒645**) that the absolute values of the parameters should be interpreted with caution, due to their sensitivity to model parameterization.

Below we outline all the checks and sensitivity analyses we conducted to ascertain the cause of the improbably small extinction fraction values, to assure the editor that we have carefully probed these results and fully understand what generated this behavior.

First, we confirmed that there were no obvious errors in our code that were driving the implausibly small parameter estimates. We checked that both the fossil-inclusive and extant-only phylogenies were expressed in the same units (millions of years), and we verified that the extinction fraction and net diversification estimates reported from the model were mathematically consistent with the corresponding speciation and extinction values when we calculated them manually. Thus, the very small extinction estimates did not result from a conversion or parameter extraction issue.

Second, we examined the distribution of branch lengths in the fossil-inclusive phylogenies. Although the fossil-inclusive phylogeny contained additional short branches, inspection of the branch-length distributions revealed that it did not contain substantially more short branches than the extant-only phylogeny. Furthermore, the extremely low extinction estimates were robust to phylogenetic uncertainty: all 100 fossil-inclusive trees yielded extinction fractions on the order of 10^-9^. Thus, the pattern was not driven by a small number of anomalous tree topologies or unusually short branches.

Third, we conducted several additional model comparisons to determine whether the result was caused by how we had specified our HiSSE models. We found that the same behavior occurred when we varied the sampling fraction and when we fitted alternative diversification models using the same fossil-inclusive tree. For a representative fossil-inclusive tree, we increased the sampling fractions from 0.1 to 0.75 (to match the value used for the extant-only analyses), but this did not appreciably change the result: extinction fraction estimates remained on the order of 10^-9^. Similarly, fossil-inclusive CID-2, BiSSE, and HiSSE models all produced extinction fractions on the order of 10^-9^: CID-2 yielded extinction fractions of 2.92×10^-9^ and 4.30×10^-9^, BiSSE yielded 2.06×10^-9^ for both states, and HiSSE yielded 2.06‒2.10×10^-9^ across its four states. This indicated that the extremely small extinction fractions were not a consequence of the particular HiSSE parameterization we had employed.

We also directly compared turnover and extinction fraction estimates for the same tree when we specified that it either did or did not include fossils (‘includes.fossils = TRUE/FALSE’). Revealingly, we found that turnover estimates were nearly identical between the two approaches, whereas extinction fractions differed by several orders of magnitude. When we specified that the tree contained fossils, turnover was approximately 0.293 and 0.378 for the two observed states, while extinction fractions were approximately 2×10^-9^ for both states. When we specified that the tree did **not** contain fossils, turnover was unchanged (0.293 and 0.378), but extinction fractions increased to 0.068 and 0.662. Thus, specifying that fossils were included in the phylogeny resulted in very different estimations of extinction fractions, with the fossil-inclusive models recovering extinction fractions very close to zero.

In inspecting the ‘hisse’ (version 2.1.12) source code to understand the cause of this behavior, we found that the package optimizes diversification parameters on a log-scale with a lower bound of -20. This lower bound corresponds to parameter estimates of 2.06×10^-9^, very similar to the extinction fraction estimates recovered in our analyses. When we relaxed this lower bound to -30 for a representative tree, the estimated extinction fraction declined to ~10^-14^, demonstrating that values we recovered were indeed arbitrary, corresponding to the boundary specified within ‘hisse’: essentially, the likelihood algorithm was trying to fit an extinction fraction of zero but could not assign values lower than 2.06×10^-9^. Thus, the originally reported extinction fraction values were effectively boundary estimates and not biologically meaningful, as the academic editor suspected.

By further inspecting the source code, we ascertained that ‘hisse’ was not simply treating extinct taxa as additional tips (as we had assumed), but was rather invoking a separate fossil-specific likelihood framework which included an additional fossilization/sampling parameter. We determined that ‘hisse’ uses this additional parameter to estimate the extinction module of diversification dynamics using records of fossil occurrences (a ‘k.samples table’). Because we did not have such a record to provide to the model, our fossil-inclusive models were essentially estimating no extinction whatsoever, having no records of extinction events from which to estimate the extinction module of the diversification dynamics. This is why the extinction fraction estimates in our fossil-inclusive analyses were pushed towards the lower bound allowed by ‘hisse’, that of *~*10^-9^.

We have thus changed how we specified all our fossil-inclusive models (CID-2, BiSSE, and HiSSE) so that extinct taxa are handled appropriately by the ‘hisse’ package. This resulted in much more plausible estimates of diversification parameters. Importantly, our central findings were robust to these changes: we found that HiSSE models were still generally preferred for most tree topologies (93/100), and we found that migration continued to predict parameter variation better than other characteristics in general (in 492/500 cases). We also found that migratory lineages continued to exhibit elevated speciation and extinction rates, and consequently elevated lineage turnover and net diversification rates, relative to non-migratory lineages, though the effects of migration on extinction fraction still varied across trees. These results, particularly those regarding elevated extinction and speciation rates within migratory lineages, have been stable to all the different approaches and implementations we have tried, both in our troubleshooting here as well as the other analytical modifications we made during the previous round of revision. We view this as compelling evidence that these patterns are genuine and not an artifact of model specification or parameterization choices.

That said, we agree with the underlying concern advanced by the editor, that the absolute magnitude of these extinction rate estimates should not be over-interpreted. In the revised manuscript, we have thus been careful to anchor our interpretation not in the absolute magnitude of these parameters, but rather on the direction and consistency of differences between migratory and non-migratory lineages across phylogenetic trees and analytical approaches. We have revised the manuscript to de-emphasize the numerical diversification parameter estimates and to more explicitly acknowledge the issue of parameter non-identifiability inherent to such state-dependent diversification approaches.

In addition to these revisions, you may need to complete some formatting changes, which you will receive in a follow up email. A member of our team will be in touch with a set of requests shortly. If you do not receive a separate email within a few days, please assume that checks have been completed, and no additional changes are required.

We were notified in a follow-up email that “unfortunately [the map graphics that we employed in our figures, which were Adobe Stock images, were] not compatible with *PLOS*’s copyright requirements as we require a CCBY 4.0. The ‘Education license’ is still too restrictive”. We have therefore substituted these graphics for equivalent ones generated using the R package ‘maps’, an approach which we confirmed with the *PLOS Biology* Editorial Office was acceptable. We have also corrected the figure captions accordingly.
