# Supplementary material for: Phylogenetic evidence that migratory behavior increases ungulate extinction rates
Source: PLoS Biol. 2026 Nov 5;24(11):e3004072. doi: 10.1371/journal.pbio.3004072 (PMC13739097; doi:10.1371/journal.pbio.3004072)
Supplement: Supplementary file 5 [file pbio.3004072.s008.docx]

25 September 2026

Dr. Ankiit Ahluwalia

Associate Editor, *PLOS Biology*

Dear Dr. Ahluwalia,

We are pleased to submit a revised version of our manuscript, “**Phylogenetic evidence that migratory behavior increases ungulate extinction rates**” (PBIOLOGY-D-25-03497R3), for your consideration for *PLOS Biology*.

We are grateful to the Academic Editor for identifying this outstanding imprecision in our description of our results. We have modified the relevant sections of text to more accurately communicate our findings. We have also made all of the outstanding changes requested by the *PLOS Biology* editorial team, including depositing all the data underlying the analyses and figures into a Dryad Data Repository and noting in the figure captions where the data can be found. The temporary link to the Dryad repository while it is under curation is: http://datadryad.org/share/LINK_NOT_FOR_PUBLICATION/r6cIJR7JM8GJl8aBUubCTwI9R2JB1Nu_x9o735hG_GI

The permanent link, for use after the repository is released from curation and thus included in the manuscript files, is: https://doi.org/10.5061/dryad.jm63xsjsp

We hope that the changes we have made fully address the concern raised by the Academic Editor, and we thank them again for their careful reading of our manuscript.

Sincerely,

Joel Abraham, *Princeton University*

[joeloa@princeton.edu](mailto:joeloa@princeton.edu)

***PLOS Biology* Editors:**

a) Overall, the Academic Editor is largely satisfied and has provided some comments for your consideration (below).

We thank the Academic Editor for noting this outstanding inaccuracy in the text. We have edited the relevant sections of text to more precisely communicate our findings (please see response to the Academic Editor below for the specific changes made).

b) Please address my Data Policy requests below; specifically, we need you to supply the numerical values underlying Fig. 1a-c, Fig. 2a-e, Fig. 3a-f, Fig. S1a-f, Fig. S2a-o, Fig. S3a-e, Fig. S4a-o, Fig. S5a-e, Fig. S6a-e.

We have deposited files containing all the data underlying all of the figures in this paper into the associated Dryad Data Repository. For added transparency and reproducibility, we have noted in the file descriptions included in the README file in the repository which files were used to generate which figures.

The temporary link (for use while the dataset is in curation) to the Dryad repository is: http://datadryad.org/share/LINK_NOT_FOR_PUBLICATION/r6cIJR7JM8GJl8aBUubCTwI9R2JB1Nu_x9o735hG_GI

The permanent link (once the dataset is published) to the Dryad repository is: https://doi.org/10.5061/dryad.jm63xsjsp

c) Please make the data deposited in Dryad publicly available (with a DOI) before the manuscript enters Production.

We have deposited all data underlying the analyses and the figures in the aforementioned Dryad Data Repository (https://doi.org/10.5061/dryad.jm63xsjsp), along with the code used to conduct the analyses and generate the figures.

d) Please clearly cite the location of the data in all relevant main and supplementary figure legends, for example: “The data underlying this figure can be found at doi.org/10.xxx/dryad/zenodo.xxxxx.” We understand that this may appear repetitive, but it helps make the figures and their legends more self contained.

We have specified in all figure captions of both the main text and supporting information figures where the data underlying each of the figures can be found. As noted above, all the data used to generate the figures are available in the Dryad Data Repository (<https://doi.org/10.5061/dryad.jm63xsjsp>) as well as in the supporting information (**S1 Data** and **S2 Data**).

e) Please make any custom code available either part of your data deposition or as a supplementary file.

The code used to conduct all of the analyses in the paper is likewise available in the Dryad Data Repository (https://doi.org/10.5061/dryad.jm63xsjsp)

f). Please include the URLs of your funders in the Financial Disclosure statement.

We have added the URLs of all the funding organizations to the Financial Disclosure statement.

**Academic Editor:**

The paper is almost ready for publication but there is one point that needs to be addressed before we accept it. In two passages of the paper the results are slightly mis-interpreted, or at least used a non-adequate choice of words. On lines 196 to 200 it is written: “When extinct taxa were included, HiSSE models were preferred (i.e., lower AICc) to BiSSE (Binary State Speciation and Extinction; no hidden states) and CID-2 (character-independent diversification; only hidden states) models for 93 of 100 tree conformations (Table S3), indicating that migratory behavior generally explained parameter heterogeneity better than models lacking hidden states or trait dependence;” This sentence might give the impression that it is implied that migration is the sole more important factor, while it is not. If migration was the sole most important factor then the BiSSE model would be preferred. In that sense, it is weird to say that “migratory behavior generally explained parameter heterogeneity better than models lacking hidden states” because the model lacking hidden state would be the sole migration effect. Finding that HiSSE model is the best model implies that migration has some effect but there are other factors that are also relevant to explain the rate heterogeneity. The wording needs to be fixed here to make explicit that the HiSSE model suggests that migration has some effect but there are also other factors that affect/explain speciation and extinction dynamics. The same applies to the text on lines 314 to 319 in the first paragraph in the discussion.

We thank the Academic Editor for noting this outstanding imprecision in our description of our results. We agree that our original wording could be interpreted as suggesting that migration is the sole driver of macroevolutionary rate heterogeneity, whereas our model comparisons indicate that additional, unmeasured factors also contribute to variation in diversification dynamics. We have therefore revised the *Results* (**L196-203**) to clarify that our model comparisons support a role for migration in explaining macroevolutionary rate variation alongside additional, unmeasured factors. The quoted text now reads: “When extinct taxa were included, HiSSE models were preferred (*i.e.,* lower AIC_c_) to BiSSE (Binary State Speciation and Extinction; no hidden states) and CID-2 (character-independent diversification; only hidden states) models for 93 of 100 tree conformations (Table S3), indicating that models featuring migration-dependent diversification generally explained parameter heterogeneity better than models lacking trait dependence, though the inclusion of hidden states suggested that additional factors contributed to rate heterogeneity as well; for the remaining seven trees, BiSSE models were preferred in two cases and CID-2 models were preferred in five cases (Table S3)”.

We have also revised the concluding sentence of the paragraph (**L208-211**) to further emphasize that other factors contribute to structuring parameter heterogeneity: “Together, these findings suggest that, while other factors contribute to macroevolutionary parameter heterogeneity across lineages, migration is a stronger predictor of this heterogeneity than these correlated traits and plays a foremost role in structuring ungulate macroevolutionary dynamics”.

Regarding the passage in the *Discussion* (**L316-321**), our intention was to summarize the differences in reconstructed macroevolutionary parameters between migratory and non-migratory lineages, rather than to suggest that migration alone was responsible for those differences. However, we recognize that this point could be made more explicit. We have therefore revised this passage to acknowledge that, although migratory lineages exhibited elevated rates in our fossil-inclusive analyses, our model comparison results also indicated that additional, unmeasured factors contribute to macroevolutionary parameter heterogeneity as well. The text now reads: “Then, using Hidden State Speciation and Extinction (HiSSE) modeling to reconstruct macroevolutionary parameters, we found: (2) fossil-inclusive macroevolutionary parameter reconstructions indicated that migratory lineages experienced elevated speciation and extinction rates, as well as elevated lineage turnover and net diversification (Fig. 2), though the inclusion of hidden states suggested that other unaccounted-for factors also contributed to parameter heterogeneity (Table S3)”.

We believe these revisions more precisely communicate our results, and we thank the Academic Editor again for drawing our attention to these sections of text.
