# Supplementary material for: Phylogenetic evidence that migratory behavior increases ungulate extinction rates
Source: PLoS Biol. 2026 Nov 5;24(11):e3004072. doi: 10.1371/journal.pbio.3004072 (PMC13739097; doi:10.1371/journal.pbio.3004072)
Supplement: Peer review history

# Editor report: Decision Letter 0

**DOI:** 10.1371/journal.pbio.3004072.r001

**Authors:** Ankiit Ahluwalia

6 Nov 2025

Dear Dr Abraham,

Thank you for submitting your manuscript entitled "Migratory behavior increases extinction risk within ungulate" for consideration as a Research Article by PLOS Biology.

Your manuscript has now been evaluated by the PLOS Biology editorial staff [as well as by an academic editor with relevant expertise] and I am writing to let you know that we would like to send your submission out for external peer review.

We think that your manuscript would be better considered as a Short Report. No re-formatting is needed at this stage, but please select "Short Reports" as the article type when you upload your additional metadata (see next paragraph).

However, before we can send your manuscript to reviewers, we need you to complete your submission by providing the metadata that is required for full assessment. To this end, please login to Editorial Manager where you will find the paper in the 'Submissions Needing Revisions' folder on your homepage. Please click 'Revise Submission' from the Action Links and complete all additional questions in the submission questionnaire.

Once your full submission is complete, your paper will undergo a series of checks in preparation for peer review. After your manuscript has passed the checks it will be sent out for review. To provide the metadata for your submission, please Login to Editorial Manager (https://www.editorialmanager.com/pbiology) within two working days, i.e. by Nov 10 2025 11:59PM.

If your manuscript has been previously peer-reviewed at another journal, PLOS Biology is willing to work with those reviews in order to avoid re-starting the process. Submission of the previous reviews is entirely optional and our ability to use them effectively will depend on the willingness of the previous journal to confirm the content of the reports and share the reviewer identities. Please note that we reserve the right to invite additional reviewers if we consider that additional/independent reviewers are needed, although we aim to avoid this as far as possible. In our experience, working with previous reviews does save time.

If you would like us to consider previous reviewer reports, please edit your cover letter to let us know and include the name of the journal where the work was previously considered and the manuscript ID it was given. In addition, please upload a response to the reviews as a 'Prior Peer Review' file type, which should include the reports in full and a point-by-point reply detailing how you have or plan to address the reviewers' concerns.

During the process of completing your manuscript submission, you will be invited to opt-in to posting your pre-review manuscript as a bioRxiv preprint. Visit http://journals.plos.org/plosbiology/s/preprints for full details. If you consent to posting your current manuscript as a preprint, please upload a single Preprint PDF.

Feel free to email us at plosbiology@plos.org if you have any queries relating to your submission.

Kind regards,

Ankiit Ahluwalia,

PLOS Biology

aahluwalia@plos.org

# Author comment: Author response to Decision Letter 1

**DOI:** 10.1371/journal.pbio.3004072.r002

10 Nov 2025

Attachment Submitted filename: Abraham et al._PLOS Bio_response to reviewers.docx

# Peer review: Decision Letter 1

**DOI:** 10.1371/journal.pbio.3004072.r003

**Authors:** Ankiit Ahluwalia

27 Jan 2026

Dear Dr Abraham,

Thank you for your patience while your revised manuscript "Migratory behavior increases extinction risk within ungulates" was peer-reviewed at PLOS Biology. It has now been evaluated by the PLOS Biology editors, an Academic Editor with relevant expertise, and by two independent reviewers.

In light of the reviews, which you will find at the end of this email, we would like to invite you to revise the work to thoroughly address the reviewers' reports.

IMPORTANT:

a) Although we made use of your previous reviews, we were not able to obtain the reviewer identities from the upstream journal. As a result, with advice from our Academic Editor, we therefore sought opinions from two new reviewers, asking them to assess the manuscript itself and your responses to the previous reviews.

b) Unfortunately we were not able to obtain additional advice on the decision from our Academic Editor, who is currently travelling. As a result, it is possible that they may have further requests or guidance for you; if they send us such advice, we will forward it to you.

You'll see that both reviewers are positive and find the study interesting and worth pursuing, but they also raise several points that would need to be addressed before we can consider the manuscript for publication. Reviewer #1 main suggestions are to fit the same models without the fossil species to avoid biases that the fossil species might be incorporating to the structure of the data that would artificially reinforce the patterns. There are some concerns on how “sampling fractions” are calculated and it is linked with whether fossil species are included or not (see above). There are also suggestions to fit a model with all parameters free to be estimated, and then compare values and base their conclusions on the full set of models. Reviewer #2 main concerns are related to the interpretation of the HiSSE diversification results. Major concerns include problematic interpretations with conclusions from their HiSSE analyses that migratory lineages were associated with both higher extinction and higher speciation rates than non-migratory lineages. Another concern is related to assumptions of the phylogenetic imputations and suggested authors to clarify the assumptions and discuss the implications of their chosen model.

Given the extent of revision needed, we cannot make a decision about publication until we have seen the revised manuscript and your response to the reviewers' comments. Your revised manuscript is likely to be sent for further evaluation by all or a subset of the reviewers.

In addition to these revisions, you will need to complete some formatting changes, which you will receive in a follow up email. A member of our team will be in touch with a set of requests shortly.

We expect to receive your revised manuscript within 3 months. Please email us (plosbiology@plos.org) if you have any questions or concerns, or would like to request an extension.

At this stage, your manuscript remains formally under active consideration at our journal; please notify us by email if you do not intend to submit a revision so that we may withdraw it.

**IMPORTANT - SUBMITTING YOUR REVISION**

Your revisions should address the specific points made by each reviewer. Please submit the following files along with your revised manuscript:

1. A 'Response to Reviewers' file - this should detail your responses to the editorial requests, present a point-by-point response to all of the reviewers' comments, and indicate the changes made to the manuscript.

*NOTE: In your point-by-point response to the reviewers, please provide the full context of each review. Do not selectively quote paragraphs or sentences to reply to. The entire set of reviewer comments should be present in full and each specific point should be responded to individually, point by point.

You should also cite any additional relevant literature that has been published since the original submission and mention any additional citations in your response.

2. In addition to a clean copy of the manuscript, please also upload a 'track-changes' version of your manuscript that specifies the edits made. This should be uploaded as a "Revised Article with Changes Highlighted" file type.

*Re-submission Checklist*

When you are ready to resubmit your revised manuscript, please refer to this re-submission checklist: https://plos.io/Biology_Checklist

To submit a revised version of your manuscript, please go to https://www.editorialmanager.com/pbiology/ and log in as an Author. Click the link labelled 'Submissions Needing Revision' where you will find your submission record.

Please make sure to read the following important policies and guidelines while preparing your revision:

*Published Peer Review*

Please note while forming your response, if your article is accepted, you may have the opportunity to make the peer review history publicly available. The record will include editor decision letters (with reviews) and your responses to reviewer comments. If eligible, we will contact you to opt in or out. Please see here for more details:

https://blogs.plos.org/plos/2019/05/plos-journals-now-open-for-published-peer-review/

*PLOS Data Policy*

Please note that as a condition of publication PLOS' data policy (http://journals.plos.org/plosbiology/s/data-availability) requires that you make available all data used to draw the conclusions arrived at in your manuscript. If you have not already done so, you must include any data used in your manuscript either in appropriate repositories, within the body of the manuscript, or as supporting information (N.B. this includes any numerical values that were used to generate graphs, histograms etc.). For an example see here: http://www.plosbiology.org/article/info%3Adoi%2F10.1371%2Fjournal.pbio.1001908#s5

*Blot and Gel Data Policy*

We require the original, uncropped and minimally adjusted images supporting all blot and gel results reported in an article's figures or Supporting Information files. We will require these files before a manuscript can be accepted so please prepare them now, if you have not already uploaded them. Please carefully read our guidelines for how to prepare and upload this data: https://journals.plos.org/plosbiology/s/figures#loc-blot-and-gel-reporting-requirements

*Protocols deposition*

To enhance the reproducibility of your results, we recommend that if applicable you deposit your laboratory protocols in protocols.io, where a protocol can be assigned its own identifier (DOI) such that it can be cited independently in the future. Additionally, PLOS ONE offers an option for publishing peer-reviewed Lab Protocol articles, which describe protocols hosted on protocols.io. Read more information on sharing protocols at https://plos.org/protocols?utm_medium=editorial-email&utm_source=authorletters&utm_campaign=protocols

Thank you again for your submission to our journal. We hope that our editorial process has been constructive thus far, and we welcome your feedback at any time. Please don't hesitate to contact us if you have any questions or comments.

Sincerely,

Ankiit

Ankiit Ahluwalia,

PLOS Biology

aahluwalia@plos.org

------------------------------------

REVIEWS:

Reviewer #1: This work intends to assess the impact of a very important behavioral trait (migration) in the extinction rates in ungulates. They use data from both extant and extinct species to estimate diversification rates and the occupation of the morphospace, using the results of these analyses to get a deeper understanding of the effects of migration. The manuscript is well written, with good contextualization and clear logic.

I must state from the beginning that I was not an original reviewer for this manuscript, neither here nor for the previous journal (as I found out after reading the reply letter). That being said, my comments will be mostly related to the implementation of the methods and the interpretation of the results. I believe the authors have overlooked a few caveats, as well as not interpreted the results as they should, and both of these issues might undermine the ability to draw conclusions from the results. I believe these issues are important enough to deserve a careful re-examination of the results in the light of my (hopefully useful) comments below.

Major points:

Title: I tend to concur with reviewer #1 about the title, because "extinction risk" is usually used in a context of conservation, which might lead to misunderstandings (I even informally got some feedback from colleagues - not disclosing what it was for nor revealing any information about the manuscript - and the impression was unanimously identical to mine. I would change it to "extinction probabilities" or "rates" or something along those lines.

Lines 130-132: This comparison is tricky: I'd agree that this difference could mean something if the fossil species were distributed evenly across the phylogeny; since it isn't properly the case, there is a chance that the sample is biased (maybe migratory species have more chances of entering the fossil record by expanding the localities in which they can be preserved, or even some body size trends - if I'm not wrong, migrants tend to be larger, and being larger helps preservation). Thus, I would be careful to interpret this at face value.

Lines 440-443: Some detail about the models could help, even if in the supplementary material.

Line 456: It would be important to fit the same models without the fossil species. The combined approach of Rphylopars+Threshold is basically a BM+Threshold (given that as far as I know Rphylopars does not have any OU model implemented for non-ultrametric trees), which would be the "continuous-zation" (for the lack of a better word) of an Mk model that is the same used in HiSSE. This way, by not assessing the results excluding the fossl species, you could be prone to biases that the fossil species are incorporating to the structure of the data that would artificially reinforce the patterns.

My feeling is that you can be artificially inflating extinction rates related to migratory species depending on how these species are distributed across the phylogeny. Based on Fig 1a, the fossils already show some phylogenetic bias, and that could be exacerbated if the clustering of migrant/non-migrant species is also important. It might be that the results hold, but if they are not super strong, maybe this artificial reinforcement is driving it to becoming "significant".

Lastly, this connects to the issue of fixing extinction fractions in a way (next point).

Lines 457-458: I think this is actually wrong. If I am not wrong, sampling fraction is typically calculated in relation to the total number of species in the clade, and was thought as a way to help inform the models about how many splitting events might be missing. Thus, most commonly the sampling fraction is calculated in relation to the total number of extant species in a phylogeny. Therefore, adding fossils to the phylogeny will not amount to the total extant richness which would needed to calculate sampling fraction. If the authors really wanted to calculate an equivalent metric, they should consider all species from the group that ever lived. I don't know exactly how HiSSE deals with the fossil species, but this might have implications in the results in case the authors added the fossil species when calculating sampling fraction, given that the model will be misinformed that the data is closer to the true extant diversity than it actually is.

Lines 460-462: I wonder how holding extinction fractions constant could impact your conclusion given that the central message of the paper is related to extinction rate differences. I am fully aware that estimating extinction rates from phylogenies is tricky, and I am assuming that the authors made this decision to simplify the model, but as I said, given that the core of the paper is related to extinction rates, in my opinion holding the extinction fraction constant has a direct impact in the results and conclusions. I would therefore suggest that the authors fit a model with all parameters free to be estimated, and then compare AICc, parameter values, etc., and base their conclusions on the full set of models.

Line 470: They are also associated to the marginal probability of being on hidden state A or B. Therefore, the authors should show and compare the distribution of rates for each of the 4 estates (MigA, MigB, NMigA, NMig B). If they lump them all together, the purpose of using HiSSE is lost and the false positives might still be taking place.

Lines 479-481: One or more of those traits may be acting within migratory/non-migratory species, generating rate heterogeneity within each category and even driving the diversification patterns in lieu of the mapped trait (migratory behavior). That's the purpose of the hidden state, as stated in the previous point.

Line 492: I don't get how a trait space is constructed using the distances between pairs of species. To me, the distances come exactly FROM the trait space. This seems very circular and confusing.

Figure 3: I understand that the overlap between yellow and blue becomes green, but I think the color in this figure could be rethought. I would represent the polygon for the extinct species on its own, separate from the extant, instead of having it as extinct + extant. It is easy enough to visualize the sum of everything as the union of both polygons, and by splitting the categories you add some more information in terms of whether the space has shifted, expanded, etc.

Minor point:

Line 188: You could show the distributions of rate differences to highlight how distinct are the rates

Reviewer #2: This manuscript tackles an important topic from an original angle, and I believe it deserves to be published. The central question, whether migratory behavior is causally linked to higher extinction rates, not only for extant species under Anthropocene global change but also at macroevolutionary timescales, is timely and clearly relevant. Climatic and environmental change, whether anthropogenic or driven by natural geological events, can disrupt resource-availability pathways by reducing availability and increasing unpredictability, and these effects may extend into deep evolutionary timescales. Under such conditions, the authors conclude, migration may enhance extinction risk over evolutionary time.

The authors first address whether migratory behavior was disproportionately represented among extinct species by compiling data on ungulate species that went extinct over the last ~63,000 years and using phylogenetic imputation to infer migratory behavior in these taxa. Because migration is difficult to infer directly from the fossil record, they rely on three continuous traits previously shown to correlate with migratory behavior in extant species. A literature-validation indicates support for the inferred migratory status in approximately 20% of extinct taxa. The authors then test for correlations between migratory behavior and tip diversification rates (speciation and extinction), inferred under a HiSSE model, using phylogenetic generalized linear models.

The authors conclude that migration is an evolutionarily fragile strategy, especially as increasingly widespread and intensive anthropogenic activity disrupts resource gradients (e.g., through infrastructure development) and increases environmental unpredictability (e.g., via climate change).

Overall, the methodology is sophisticated, the data appear robust, and the study addresses a biologically important question. However, I believe that some conclusions, specifically those related to diversification dynamics, are overstretched, given the well-known difficulties in disentangling speciation and extinction rates from extant-only phylogenies over deep macroevolutionary timescales. My main concern, therefore, relates to the interpretation of the HiSSE diversification results.

To clarify, I am relatively confident in the conclusion that a high proportion of extinct species were migratory, based on the phylogenetic imputation analyses. In contrast, I am less convinced by the inference that migratory lineages experimented higher extinction rates over evolutionary time and therefore that this is a labile macroevolutionary strategy. I suspect this might be an artifact of parameter non-identifiability in the diversification analyses. I note that my concerns are not related to the parameter non-identifiability highlighted by Louca and Pennell (Science, 2020) for continuous-time varying birth-death models. SSE models like HiSSE estimate parameters using numerical integration of ODEs rather than closed-form likelihoods expressed as deterministic lineage-through-time plots, as in Louca & Pennell, so the same identifiability issues do not directly apply.

Major points:

1. Speciation-extinction non-identifiability.

The authors conclude from their HiSSE analyses that migratory lineages were associated with both higher extinction and higher speciation rates than non-migratory lineages. They attribute this pattern to reduced access to seasonal resources under climatic and geological change, which may not compensate for the substantial energetic costs of migration (leading to extinction), combined with barriers to gene flow that isolate populations and promote speciation.

I find this interpretation problematic. Speciation and extinction are "notoriously difficult" to estimate separately from extant-only phylogenies. Consequently, most diversification software, including hisse, reparameterize these rates into composite parameters that behave better in likelihood space. In hisse, the core parameters are turnover: τ = λ+μ, and extinction fraction: ε = μ / λ, from which speciation and extinction are derived analytically. Although this reparameterization reduces pathological likelihood ridges caused by strong correlations between λ and μ, it also complicates biological interpretation.

Two results call my attention. First, net diversification rates are negative in both migratory and non-migratory ungulates (i.e., extinction exceeds speciation in both cases). Second, estimated extinction and speciation rates are very similar between the two categories, differing only slightly in absolute value (migratory: λ = 2.86, μ = 1.03; non-migratory: λ = 2.84; μ = 1.01).

I suspect that these nearly identical trends and values indicate that speciation and extinction cannot be really disentangled in these analyses, a pattern that is consistent with my own experience using HiSSE implementations in a Bayesian framework (using RevBayes), where likelihood ridges often lead to non-identifiability of these parameters.

If I understand the authors' interpretation correctly, they argue that "higher turnover combined with lower net diversification" in migratory lineages indicates higher extinction rates relative to non-migratory lineages. However, high turnover combined with low net diversification indicates rapid lineage replacement, as the authors themselves note, but does not by itself provide strong evidence for elevated extinction (given the limited identifiability of extinction rates from extant-only phylogenies mentioned above). Only if extinction fraction were demonstrated higher in migratory lineages could one reasonably conclude that extinction contributed disproportionately to their diversification dynamics. Using the averaged speciation and extinction rates reported in lines 185-208, I calculate extinction fractions of 1.007 for migratory lineages 1.019 for non-migratory lineages, suggesting slightly higher relative extinction in non-migratory taxa, a result that contradicts the authors' conclusions. I could not find the extinction fraction reported in the manuscript. I therefore suggest that extinction fraction is displayed directly in Figure 2, together with net diversification, as it is more informative for interpreting extinction dynamics than turnover.

Also, how should the negative net diversification rates in both categories be interpreted biologically? And can the authors explain why confidence intervals are substantially wider for migratory species (Figure 2)?

2. Phylogenetic imputation.

Phylogenetic imputation relies on the R package "phylopars", which performs multiple imputation and thus incorporates imputation uncertainty, but only for continuous traits. This limitation "forces" the authors to adopt an indirect approach: migratory behavior, a binary trait, is first coded as 0/1 and treated as continuous; imputed continuous values are then converted back to binary states using a fixed threshold value (> 0.5, migratory; < 0.5, non-migratory).

Although this approach is pragmatic and it gives intuitive results, I think it relies on strong assumptions about the underlying evolutionary process and also collapses probability uncertainty into point estimates. Brownian diffusion implies continuous, symmetric change and increasing variance with time. In addition, thresholding with a fixed value converts probabilistic estimates into fixed classifications, although in this case the authors use multiple imputation to incorporate uncertainty in downstream analyses. By contrast, a CTMC model (which treats migration as an intrinsically discrete trait) allows for instantaneous transitions between binary states, asymmetric gain and loss rates, and, by bypassing thresholding, retains uncertainty throughout the inference. I am not suggesting that this invalidates the authors' imputation results, but I suggest that the authors clarify the assumptions and discuss the implications of their chosen model. For example, do you think that there is an underlying continuous liability in migratory behavior, e.g. a "degree of migratoriness"?

Moreover, alternative software exists that can perform phylogenetic imputation directly for discrete traits with non-Gaussian distributions. For example, RevBayes can estimate posterior distributions for missing tip states under binary CTMC or multistate Markov models, with uncertainty propagation handled naturally through Bayesian joint modeling. Similarly, the R package MCMCglmm can accommodate binary traits via threshold models within a Bayesian framework.

Because phylogenetic imputation results depend strongly on the assumed evolutionary model, the manuscript should state explicitly which continuous trait evolution model was used in phylopars (I assume it was a Brownian motion model), and whether alternative models were explored (e.g., OU). As implemented, I understand that your analysis assumes a constant rate of trait evolution through time, but it is conceivable that this rate might have increased, decreased, or even approached zero during particular intervals of evolutionary history, in which case time-varying BM, Early Burst, or ACDC models might be more appropriate. Additionally, imputation assumes that missingness is approximately phylogenetically random. Are particular families or genera disproportionately represented among the extinct taxa in your compiled dataset?

I hope that these comments are helpful in further strengthening an already interesting and timely manuscript.

# Author comment: Author response to Decision Letter 2

**DOI:** 10.1371/journal.pbio.3004072.r004

19 Jun 2026

Attachment Submitted filename: PLOS Bio_response to reviewers.docx

# Peer review: Decision Letter 2

**DOI:** 10.1371/journal.pbio.3004072.r005

**Authors:** Ankiit Ahluwalia

12 Aug 2026

Dear Dr Abraham,

Thank you for your patience while we considered your revised manuscript "Phylogenetic evidence that migratory behavior increases ungulate extinction rates" for consideration as a Short Reports at PLOS Biology. Your revised study has now been evaluated by the PLOS Biology editors, the Academic Editor and the original reviewers.

Although the reviewers were satisfied, our Academic Editor (AE) has identified a further concern that will need to be addressed (see below). I am sorry that because of travel commitments, the AE had not be able to look at the revised manuscript earlier. The AE has discussed these concerns with one of the reviewers, who agreed with them. We will then assess your revised manuscript with our Academic Editor aiming to avoid further rounds of peer-review, although we might need to consult with the reviewers, depending on the nature of the revisions.

"COMMENTS FROM THE ACADEMIC EDITOR:"

After reviewing the revised version, I've identified a potentially significant issue with the new analysis. One of the central conclusions is that migratory species exhibit both higher speciation and extinction rates compared to non-migratory species, primarily based on the HiSSE analysis using the fossil-included phylogeny. My concern lies in the improbability of the absolute values for these estimates, which suggests that there may be an underlying problem with the analysis. The extinction rate estimate for migratory species is 8.759×10^-9 (0.000000008759), while for non-migratory species, it is 9.288×10^-10 (0.0000000009288). The authors assert that these rates are significantly different, acknowledging their small magnitude. For reasons I explain below, those estimates seem to indicate that something went wrong with the analysis. I have never seen estimates that low…. Moreover, even if there is no problem with the analysis itself, when we assess the biological significance of such low rates, their difference do not seem meaningful. Statistically, they may differ, but biologically, both amounts are effectively zero extinction given the time span of such lineages. That on itself is already enough to question the interpretations, and would require a different narrative, but I do suspect that the analysis has some problems.

At first glance, small estimates of extinction rate might seem expected since phylogenetic estimates often yield low extinction rates, but these values are exceptionally low. Firstly, the absolute values are virtually zero, that in combination with the estimated rate of speciation would result in a astronimically high number of species if retained for the duration of the clade (assuming, of course as a simple scenario, an exponential growth); secondly, strangely, the estimates of extinction rate when incorporating fossils result in extinction rates that are significantly smaller than those based solely on extant species by several orders of magnitudes (compare those rares to the rates of 0.118 and 0.013 when using phylogeny with only extant species). This outcome appears counterintuitive. Furthermore, how is it possible to obtain such low extinction rates when we know that several species (included in the phylogeny) are extinct? This strongly suggests that there was a problem in the estimation process. Given that a substantial part of their argument relies on this analysis, in the argument that migratory and non-migratory lineages have different extinction rates, and that these values hint at a problem, it is essential for the authors to revisit these estimates (possibly correcting a small coding error) and/or provide a justification for these unusual results, as well as their biological meaning for their interpretation, before the paper can be considered for publication.

In addition to these revisions, you may need to complete some formatting changes, which you will receive in a follow up email. A member of our team will be in touch with a set of requests shortly. If you do not receive a separate email within a few days, please assume that checks have been completed, and no additional changes are required.

We expect to receive your revised manuscript within 1 month. Please email us (plosbiology@plos.org) if you have any questions or concerns, or would like to request an extension.

At this stage, your manuscript remains formally under active consideration at our journal; please notify us by email if you do not intend to submit a revision so that we withdraw the manuscript.

**IMPORTANT - SUBMITTING YOUR REVISION**

Your revisions should address the specific points made by each reviewer. Please submit the following files along with your revised manuscript:

1. A 'Response to Reviewers' file - this should detail your responses to the editorial requests, present a point-by-point response to all of the reviewers' comments, and indicate the changes made to the manuscript.

*NOTE: In your point-by-point response to the reviewers, please provide the full context of each review. Do not selectively quote paragraphs or sentences to reply to. The entire set of reviewer comments should be present in full and each specific point should be responded to individually.

You should also cite any additional relevant literature that has been published since the original submission and mention any additional citations in your response.

2. In addition to a clean copy of the manuscript, please also upload a 'track-changes' version of your manuscript that specifies the edits made. This should be uploaded as a "Revised Article with Changes Highlighted " file type.

*Resubmission Checklist*

When you are ready to resubmit your revised manuscript, please refer to this resubmission checklist: https://plos.io/Biology_Checklist

To submit a revised version of your manuscript, please go to https://www.editorialmanager.com/pbiology/ and log in as an Author. Click the link labelled 'Submissions Needing Revision' where you will find your submission record.

Please make sure to read the following important policies and guidelines while preparing your revision:

*Published Peer Review*

Please note while forming your response, if your article is accepted, you may have the opportunity to make the peer review history publicly available. The record will include editor decision letters (with reviews) and your responses to reviewer comments. If eligible, we will contact you to opt in or out. Please see here for more details:

https://blogs.plos.org/plos/2019/05/plos-journals-now-open-for-published-peer-review/

*PLOS Data Policy*

Please note that as a condition of publication PLOS' data policy (http://journals.plos.org/plosbiology/s/data-availability) requires that you make available all data used to draw the conclusions arrived at in your manuscript. If you have not already done so, you must include any data used in your manuscript either in appropriate repositories, within the body of the manuscript, or as supporting information (N.B. this includes any numerical values that were used to generate graphs, histograms etc.). For an example see here: http://www.plosbiology.org/article/info%3Adoi%2F10.1371%2Fjournal.pbio.1001908#s5

*Blot and Gel Data Policy*

We require the original, uncropped and minimally adjusted images supporting all blot and gel results reported in an article's figures or Supporting Information files. We will require these files before a manuscript can be accepted so please prepare them now, if you have not already uploaded them. Please carefully read our guidelines for how to prepare and upload this data: https://journals.plos.org/plosbiology/s/figures#loc-blot-and-gel-reporting-requirements

*Protocols deposition*

To enhance the reproducibility of your results, we recommend that if applicable you deposit your laboratory protocols in protocols.io, where a protocol can be assigned its own identifier (DOI) such that it can be cited independently in the future. Additionally, PLOS ONE offers an option for publishing peer-reviewed Lab Protocol articles, which describe protocols hosted on protocols.io. Read more information on sharing protocols at https://plos.org/protocols?utm_medium=editorial-email&utm_source=authorletters&utm_campaign=protocols

Thank you again for your submission to our journal. We hope that our editorial process has been constructive thus far, and we welcome your feedback at any time. Please don't hesitate to contact us if you have any questions or comments.

Sincerely,

Ankiit

Ankiit Ahluwalia,

PLOS Biology

aahluwalia@plos.org

---------------------------------------------------------------

REVIEWS:

Reviewer #1: The authors have thoroughly and satisfactorily addressed all my comments and suggestions (as they did for the other reviewer too). I commend them for the careful revision, and I believe the manuscript is now much improved and consists of a very interesting piece for the macroevolution community

Reviewer #2: I thank the authors for their thorough and constructive revision of the manuscript. They have responded comprehensively to all of my previous comments and, I believe, those of reviewer #1. These revisions have strengthened the main conclusions. I have no further suggestions and fully support publication of the manuscript in its present form.

# Author comment: Author response to Decision Letter 3

**DOI:** 10.1371/journal.pbio.3004072.r006

9 Sep 2026

Attachment Submitted filename: PLOS_Bio_response_to_reviewers_auresp_3.docx

# Editor report: Decision Letter 3

**DOI:** 10.1371/journal.pbio.3004072.r007

**Authors:** Ankiit Ahluwalia

23 Sep 2026

Dear Dr Abraham,

Thank you for your patience while we considered your revised manuscript "Phylogenetic evidence that migratory behavior increases ungulate extinction rates" for publication as a Short Reports at PLOS Biology. This revised version of your manuscript has been evaluated by the PLOS Biology editors and the Academic Editor.

Based on our Academic Editor's assessment of your revision, we are likely to accept this manuscript for publication, provided you satisfactorily address the remaining points raised by the Academic Editor. Please also make sure to address the following data and other policy-related requests.

IMPORTANT - please attend to the following:

a) Overall, the Academic Editor is largely satisfied and has provided some comments for your consideration (below).

b) Please address my Data Policy requests below; specifically, we need you to supply the numerical values underlying Fig. 1a-c, Fig. 2a-e, Fig. 3a-f, Fig. S1a-f, Fig. S2a-o, Fig. S3a-e, Fig. S4a-o, Fig. S5a-e, Fig. S6a-e.

c) Please make the data deposited in Dryad publicly available (with a DOI) before the manuscript enters Production.

d) Please clearly cite the location of the data in all relevant main and supplementary figure legends, for example: “The data underlying this figure can be found at doi.org/10.xxx/dryad/zenodo.xxxxx.” We understand that this may appear repetitive, but it helps make the figures and their legends more self contained.

e) Please make any custom code available either part of your data deposition or as a supplementary file.

f). Please include the URLs of your funders in the Financial Disclosure statement.

**COMMENTS FROM THE ACADEMIC EDITOR – LIGHTLY EDITED**

The paper is almost ready for publication but there is one point that needs to be addressed before we accept it. In two passages of the paper the results are slightly mis-interpreted, or at least used a non-adequate choice of words. On lines 196 to 200 it is written: “When extinct taxa were included, HiSSE models were preferred (i.e., lower AICc) to BiSSE (Binary State Speciation and Extinction; no hidden states) and CID-2 (character-independent diversification; only hidden states) models for 93 of 100 tree conformations (TableS3), indicating that migratory behavior generally explained parameter heterogeneity better than models lacking hidden states or trait dependence;” This sentence might give the impression that it is implied that migration is the sole more important factor, while it is not. If migration was the sole most important factor then the BiSSE model would be preferred. In that sense, it is weird to say that “ migratory behavior generally explained parameter heterogeneity better than models lacking hidden states” because the model lacking hidden state would be the sole migration effect. Finding that HiSSE model is the best model implies that migration has some effect but there are other factors that are also relevant to explain the rate heterogeneity. The wording needs to be fixed here to make explicit that the HiSSE model suggests that migration has some effect but there are also other factors that affect/explain speciation and extinction dynamics. The same applies to the text on lines 314 t o319 in the first paragraph in the discussion.

As you address these items, please take this last chance to review your reference list to ensure that it is complete and correct. If you have cited papers that have been retracted, please include the rationale for doing so in the manuscript text, or remove these references and replace them with relevant current references. Any changes to the reference list should be mentioned in the cover letter that accompanies your revised manuscript.

In addition to these revisions, you may need to complete some formatting changes, which you will receive in a follow up email. A member of our team will be in touch with a set of requests shortly. If you do not receive a separate email within a few days, please assume that checks have been completed, and no additional changes are required.

We expect to receive your revised manuscript within two weeks.

To submit your revision, please go to https://www.editorialmanager.com/pbiology/ and log in as an Author. Click the link labelled 'Submissions Needing Revision' to find your submission record. Your revised submission must include the following:

- a cover letter that should detail your responses to any editorial requests, if applicable, and whether changes have been made to the reference list

- a Response to Reviewers file that provides a detailed response to the reviewers' comments (if applicable, if not applicable please do not delete your existing 'Response to Reviewers' file.)

- a track-changes file indicating any changes that you have made to the manuscript.

NOTE: If Supporting Information files are included with your article, note that these are not copyedited and will be published as they are submitted. Please ensure that these files are legible and of high quality (at least 300 dpi) in an easily accessible file format. For this reason, please be aware that any references listed in an SI file will not be indexed. For more information, see our Supporting Information guidelines:

https://journals.plos.org/plosbiology/s/supporting-information

*Published Peer Review History*

Please note that you may have the opportunity to make the peer review history publicly available. The record will include editor decision letters (with reviews) and your responses to reviewer comments. If eligible, we will contact you to opt in or out. Please see here for more details:

https://plos.org/published-peer-review-history/

*Press*

Should you, your institution's press office or the journal office choose to press release your paper, please ensure you have opted out of Early Article Posting on the submission form. We ask that you notify us as soon as possible if you or your institution is planning to press release the article.

*Protocols deposition*

To enhance the reproducibility of your results, we recommend that if applicable you deposit your laboratory protocols in protocols.io, where a protocol can be assigned its own identifier (DOI) such that it can be cited independently in the future. Additionally, PLOS ONE offers an option for publishing peer-reviewed Lab Protocol articles, which describe protocols hosted on protocols.io. Read more information on sharing protocols at https://plos.org/protocols?utm_medium=editorial-email&utm_source=authorletters&utm_campaign=protocols

Please do not hesitate to contact me should you have any questions.

Sincerely,

Ankiit

Ankiit Ahluwalia,

aahluwalia@plos.org

PLOS Biology

------------------------------------------------------------------------

****[DELETE AS APPROPRIATE]****

ETHICS STATEMENT:

-- Please include the full name of the IACUC/ethics committee that reviewed and approved the animal care and use protocol/permit/project license. Please also include an approval number.

-- Please include the specific national or international regulations/guidelines to which your animal care and use protocol adhered. Please note that institutional or accreditation organization guidelines (such as AAALAC) do not meet this requirement.

-- Please include information about the form of consent (written/oral) given for research involving human participants. All research involving human participants must have been approved by the authors' Institutional Review Board (IRB) or an equivalent committee, and must have been conducted according to the principles expressed in the Declaration of Helsinki.

------------------------------------------------------------------------

DATA POLICY:

You may be aware of the PLOS Data Policy, which requires that all data be made available without restriction: http://journals.plos.org/plosbiology/s/data-availability. For more information, please also see this editorial: http://dx.doi.org/10.1371/journal.pbio.1001797

Note that we do not require all raw data. Rather, we ask that all individual quantitative observations that underlie the data summarized in the figures and results of your paper be made available in one of the following forms:

1) Supplementary files (e.g., excel). Please ensure that all data files are uploaded as 'Supporting Information' and are invariably referred to (in the manuscript, figure legends, and the Description field when uploading your files) using the following format verbatim: S1 Data, S2 Data, etc. Multiple panels of a single or even several figures can be included as multiple sheets in one excel file that is saved using exactly the following convention: S1_Data.xlsx (using an underscore).

2) Deposition in a publicly available repository. Please also provide the accession code or a reviewer link so that we may view your data before publication.

Regardless of the method selected, please ensure that you provide the individual numerical values that underlie the summary data displayed in the following figure panels as they are essential for readers to assess your analysis and to reproduce it:

[Figs….]

NOTE: the numerical data provided should include all replicates AND the way in which the plotted mean and errors were derived (it should not present only the mean/average values).

Please also ensure that figure legends in your manuscript include information on where the underlying data can be found, and ensure your supplemental data file/s has a legend.

Please ensure that your Data Statement in the submission system accurately describes where your data can be found.

------------------------------------------------------------------------

APPROPRIATE DATA PRESENTATION?

(If you think the paper is not using best practice, add the following)

- Please ensure that you are using best practice for statistical reporting and data presentation. These are our guidelines https://journals.plos.org/plosbiology/s/best-practices-in-research-reporting#loc-statistical-reporting and a useful resource on data presentation https://journals.plos.org/plosbiology/article?id=10.1371/journal.pbio.1002128

- If you are reporting experiments where n ≤ 5, please plot each individual data point.

------------------------------------------------------------------------

CODE POLICY

Per journal policy, if you have generated any custom code during the course of this investigation, please make it available without restrictions. Please ensure that the code is sufficiently well documented and reusable, and that your Data Statement in the Editorial Manager submission system accurately describes where your code can be found. More information on our Code Policy, what and how to share can be found here: https://journals.plos.org/plosbiology/s/code-availability

Please note that we cannot accept sole deposition of code in GitHub, as this could be changed after publication. However, you can archive this version of your publicly available GitHub code to Zenodo. Once you do this, it will generate a DOI number, which you will need to provide in the Data Accessibility Statement (you are welcome to also provide the GitHub access information). See the process for doing this here: https://docs.github.com/en/repositories/archiving-a-github-repository/referencing-and-citing-content

------------------------------------------------------------------------

SPECIES INDICATED IN THE ABSTRACT?

- Please note that per journal policy, the model system/species studied should be clearly stated in the abstract of your manuscript.

**EDITOR, CONSIDER ALSO INCLUDING ORGANISM IN THE TITLE, IF SENSICAL/RELEVANT AND/OR TO AVOID BEING MISLEADING/OVERREACHING (not always preferred)**

------------------------------------------------------------------------

BLOT AND GEL REPORTING REQUIREMENTS:

We require the original, uncropped and minimally adjusted images supporting all blot and gel results reported in an article's figures or Supporting Information files. We will require these files before a manuscript can be accepted so please prepare and upload them now. Please carefully read our guidelines for how to prepare and upload this data: https://journals.plos.org/plosbiology/s/figures#loc-blot-and-gel-reporting-requirements

------------------------------------------------------------------------

DATA NOT SHOWN?

- Please note that per journal policy, we do not allow the mention of "data not shown", "personal communication", "manuscript in preparation" or other references to data that is not publicly available or contained within this manuscript. Please either remove mention of these data or provide figures presenting the results and the data underlying the figure(s).

------------------------------------------------------------------------

Reviewer remarks:

# Author comment: Author response to Decision Letter 4

**DOI:** 10.1371/journal.pbio.3004072.r008

1 Oct 2026

Attachment Submitted filename: PLOS_Bio_response_to_reviewers_auresp_4.docx

# Editor report: Decision Letter 4

**DOI:** 10.1371/journal.pbio.3004072.r009

**Authors:** Ankiit Ahluwalia

8 Oct 2026

Dear Dr Abraham,

Thank you for the submission of your revised Short Reports "Phylogenetic evidence that migratory behavior increases ungulate extinction rates" for publication in PLOS Biology. On behalf of my colleagues and the Academic Editor, Tiago Quental, I am pleased to say that we can in principle accept your manuscript for publication, provided you address any remaining formatting and reporting issues. These will be detailed in an email you should receive within 2-3 business days from our colleagues in the journal operations team; no action is required from you until then. Please note that we will not be able to formally accept your manuscript and schedule it for publication until you have completed any requested changes.

Please take a minute to log into Editorial Manager at http://www.editorialmanager.com/pbiology/, click the "Update My Information" link at the top of the page, and update your user information to ensure an efficient production process.

PRESS

We frequently collaborate with press offices. If your institution or institutions have a press office, please notify them about your upcoming paper at this point, to enable them to help maximise its impact. If the press office is planning to promote your findings, we would be grateful if they could coordinate with biologypress@plos.org. If you have previously opted in to the early version process, we ask that you notify us immediately of any press plans so that we may opt out on your behalf.

We also ask that you take this opportunity to read our Embargo Policy regarding the discussion, promotion and media coverage of work that is yet to be published by PLOS. As your manuscript is not yet published, it is bound by the conditions of our Embargo Policy. Please be aware that this policy is in place both to ensure that any press coverage of your article is fully substantiated and to provide a direct link between such coverage and the published work. For full details of our Embargo Policy, please visit http://www.plos.org/about/media-inquiries/embargo-policy/.

Thank you again for choosing PLOS Biology for publication and supporting Open Access publishing. We look forward to publishing your study.

Sincerely,

Ankiit Ahluwalia,

PLOS Biology

aahluwalia@plos.org
